# Post-synthetic modification of a macrocyclic receptor *via* regioselective imidazolium ring-opening†

**DOI:** 10.1039/c5sc04860e

**Published:** 2016-03-09

**Authors:** Jia Shang, Brett M. Rambo, Xiang Hao, Jun-Feng Xiang, Han-Yuan Gong, Jonathan L. Sessler

**Affiliations:** a College of Chemistry, Beijing Normal University Xinjiekouwaidajie 19 Beijing 100875 P. R. China hanyuangong@bnu.edu.cn; b Department of Chemistry, The University of Texas at Austin 105 East 24th Street, Stop A5300 Austin Texas 78712-1224 USA sessler@cm.utexas.edu; c Institute of Chemistry, Chinese Academy of Sciences Zhongguancunbeiyijie 2 Beijing 100190 P. R. China; d Department of Chemistry, Shanghai University Shanghai 200444 China

## Abstract

A facile post-synthetic modification of a tetracationic tetraimidazolium macrocycle, 1^4+^ (*i.e.*, the “Texas-sized” molecular box (cyclo[2](2,6-di(1*H*-imidazol-1-yl)pyridine)[2](1,4-dimethylenebenzene)), is described. Under mild basic conditions, ring-opening of the imidazolium moieties occurs. This results in two new isomeric dicationic macrocycles. This simple yet efficient modification serves to alter the size of the molecular cavity, the charge of the macromolecular receptor, and the manner whereby it interacts with dianionic guest molecules. The isomeric mixture of imidazolium ring opened macrocycles can be synthesized in relatively high overall yield (86–93%). The reaction shows regioselectivity and the ratio of major to minor (*i.e.*, *trans* : *cis* ring-opened products) was determined to be *ca.* 3 : 1 *via*^1^H NMR spectroscopy. The major isomer, *trans*-cyclo[2]((*Z*)-*N*-(2-((6-(1*H*-imidazol-1-yl)pyridin-2-yl)amino)vinyl)formamide)[2](1,4-bismethylbenzene) hexafluorophosphate (2^2+^·2PF_6_^−^), was isolated in its pure form in 42% yield *via* recrystallization. The molecular recognition properties of 2^2+^ were investigated using a series of dianionic guests (*i.e.*, 2,6-naphthalenedicarboxylate (4), 2,6-naphthalenedisulfonate (5), and 1,5-naphthalenedisulfonate (6)) whose binding interactions with 1^4+^ have been previously reported. This allowed us to evaluate how imidazolium ring-opening affects the inherent host/guest interactions of 1^4+^. On the basis of solution spectroscopic studies (*e.g.*, ^1^H NMR, ^1^H–^1^H COSY NMR, DOSY NMR, and NOESY NMR), in tandem with mass spectrometric analyses (ESI-MS) and single-crystal X-ray diffraction studies, we conclude that opening up the macrocyclic structure (*i.e.*, converting 1^4+^ to 2^2+^) leads to considerable changes in the recognition behavior, with so-called outside binding or weak ion pair interactions, rather than pseudorotaxane formation, being favored both in solution and the solid-state. We postulate that methodologies such as those described herein could provide a means to control the molecular interactions of both free-standing macrocycles and those used to construct mechanically-interlocked molecules. Indeed, the application of hydroxide anion under the present conditions not only serves to effect the ring-opening of 1^4+^, but also pseudorotaxane structures, such as, *e.g.*, [1^4+^·4] or [1^4+^·5] derived there from.

## Abstract 2

Shown in graphic form is the use of mild basic conditions to effect the post-synthetic, ring-opening based modification of a tetraimidazolium macrocycle known as the “Texas box”. This ring opening modulates the intrinsic binding characteristics of the system.

## Graphical abstract

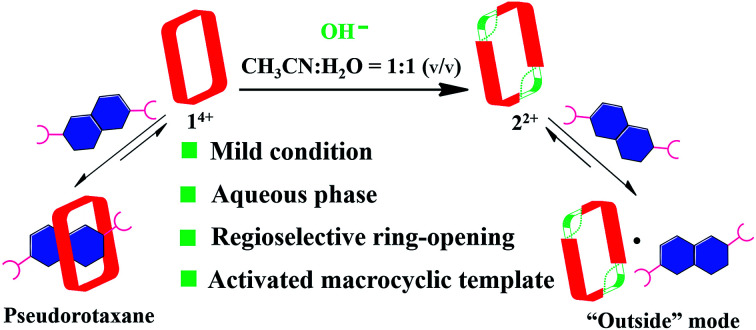

## Introduction

Macrocyclic compounds play central roles in organic, inorganic, and supramolecular chemistry. To date, macrocycles have found application in areas as diverse as host : guest chemistry, self-assembly, and the preparation of mechanically-interlocked molecules (MIMs).^1^ Tailor-made macrocycles can act as selective hosts for target analytes, as well as building blocks for complex supramolecular architectures and advanced materials.^1*c*–*f*,2^ There is thus an ongoing need to prepare new macrocycles, as well as to create analogues of existing structures with recognized utility. We believe that post-synthetic modification (PSM) strategies may have a role to play in these efforts. The appeal of post-synthetic strategies is that once a synthetic route is established, modification of the core structure may be used to access new analogues without a need to rework the synthesis. PSM may also provide a means of modulating the intrinsic binding properties of the core system in question. In the case of macrocycle-based MIMs, for instance, the ability to control recognition *via* PSM could find application in the areas of molecular switching^1*g*,*h*,2*f*,3^ and shuttling.^1*h*,3*a*,4^ While functionalized cyclodextrins,^5^ (hetero)calix[*n*]aromatics,^6,7^ and pillar[*n*]arene,^8^ have been made in this manner and the resulting systems support MIM formation, examples of PSM being used to change the macrocyclic core itself are, to our knowledge, lacking.

There are a variety of ways to prepare macrocyclic receptors. One common approach involves the use of a template, typically a loosely bound species that favors formation of a single product.^2*e*,9,10^ Many systems have been prepared using template-based approaches. In contrast, relatively few new macrocyclic systems have been prepared *via* post-synthetic modification (PSM) of known macrocyclic precursors. However, the PSM approach is attractive because it can reduce the overall synthetic burden while providing a means to alter the recognition features of the initial macrocyclic system. In an elegant example of PSM, Kohnke *et al.* used calix[6]furan as a precursor to generate efficiently calix[6]pyrrole, as well as a series of calix[*m*]furan[*n*]pyrroles (*m* = 2 or 3, *n* = 2 or 4).^11*a*^ Prior to that report, our group prepared several calix[4]pyridine and calix[*m*]pyridine[*n*]pyrroles (*m* + *n* = 4) derivatives by treating calix[4]pyrrole with a carbene to induce an insertion-based pyrrole-to-pyridine transformation.^11*b*^ Recently, Wang and colleagues reported converting a tetrazine-containing macrocycle into a novel macrocyclic structure with diaziene fragments.^11*c*–*f*^

PSM reactions involving macrocycle-directed transformations are even less common and, in the case of charged macrocyclic species are, in fact, all but unknown. One important exception involves Lehn's polyamonium macrocycles, which acted as catalysts for phosphoryl transfer in ATP hydrolysis. In this case, transient modification of the macrocycle is thought to play a role in directing the overall transformation.^12^ Other charged macromolecular receptors, including Stoddart's “blue box” (*i.e.*, cyclobis(paraquat-*p*-phenylene) or CBPQT^4+^)^13^ and the imidazolium receptors developed by Yoon, K. S. Kim and others,^14–18^ and triazole or triazolium receptors gained by our group,^19*a*^ Flood,^19*b*–*d*^ and Beer,^20^ have not yet, apparently, been subject to post-synthetic modification. On the other hand, PSM has been widely applied in the field of metal–organic frameworks as a method of functionalizing the basic system after it has been synthesized.^9*v*,21^

**Scheme 1:**
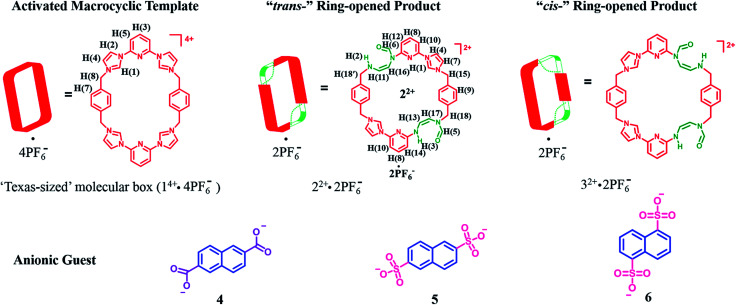
Structures of the “Texas-sized” molecular box (1^4+^·4PF_6_^−^) and its “*trans*-” and “*cis*-” imidazolium ring-opened products. Also shown are the dianionic guest molecules used in this study.

MIMs constitute another set of well-defined constructs where post-synthetic modification could be useful in terms of modulating the inherent properties. As a test of this latter hypothesis, we sought to apply a PSM approach to the modification of macrocycles that are predisposed to MIM formation. As detailed below we have found that the tetracationic tetraimidazolium macrocycle, known as the “Texas-sized box” (*i.e.*, 1^4+^·4PF_6_^−^),^22*a*,*b*^ undergoes PSM under mild basic conditions to produce isomeric dicationic products wherein two of the four imidazolium subunits have undergone ring opening to produce a pair of “*cis*-” (2^2+^) and “*trans*-” (3^2+^) constitutional isomers (Scheme 1). One of these products, 2^2+^, could be obtained in decent yield in purified form as its bis-PF_6_^−^ salt. On the basis of solution phase spectroscopic studies and X-ray diffraction analyses, it was inferred that 2^2+^ is much more flexible that its precursor 1^4+^. This structural change is reflected in the binding properties. Specifically, for dianionic guests whose binding interactions with 1^4+^ have been previously reported (*i.e.*, 2,6-naphthalenedicarboxylate (4), 2,6-naphthalenedisulfonate (5), and 1,5-naphthalenedisulfonate (6); *cf.*Scheme 1),^22*b*,*c*^ so-called outside binding, involving weak electrostatic ion pairing, rather than pseudorotaxane formation, is favored after imidazolium ring opening. Furthermore, it was found that hydroxide anion-induced imidazolium ring-opening causes irreversible disassembly of the original pseudorotaxane structures (*e.g.*, [1^4+^·4] or [1^4+^·5]). Previously, we showed proton-induced dethreading of [1^4+^·4].^22*b*^ The susceptibility to effective pH seen for [1^4+^·4] under acidic and now basic conditions mimics in a rudimentary fashion many biological interpenetrated structures, which only maintain physiological activity over a tightly regulated pH range.

## Results and discussion

The starting material for the present PSM study was 1^4+^·4PF_6_^−^ (*i.e.*, the hexafluorophosphate salt of cyclo[2](2,6-di(1*H*-imidazol-1-yl)pyridine)[2](1,4-dimethylenebenzene); Scheme 1). This tetracationic macrocycle is readily obtained *via* a two step procedure. It acts as an effective receptor for dicarboxylate and disulfonate guests, as reported recently.^22*b*,*c*^

**Scheme 2:**
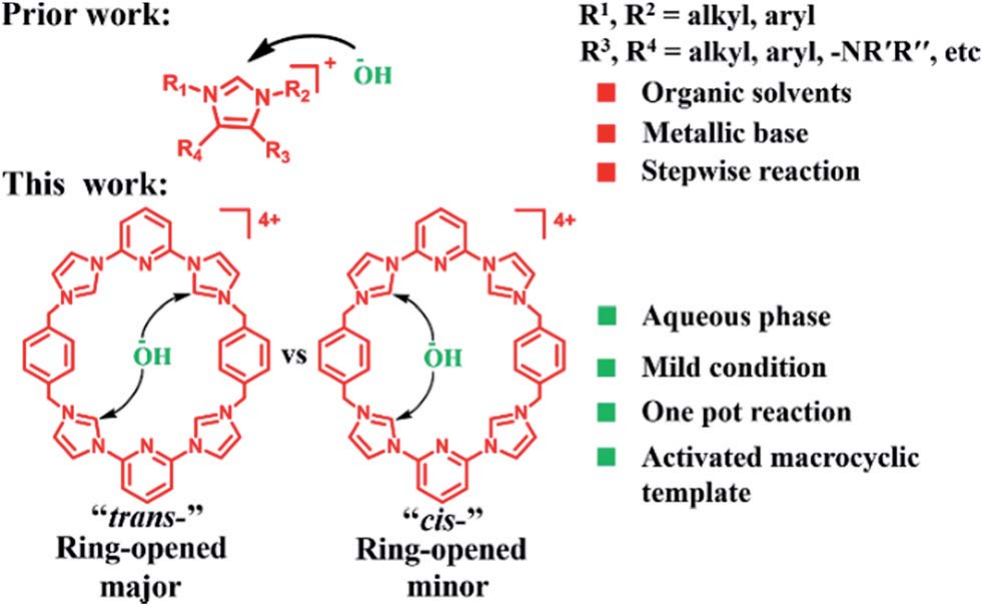
Graphical comparison of this study to prior work involving non-macrocyclic imidazolium substrates.

In the case of more highly basic guests, evidence of receptor decomposition was seen. We considered that this could reflect ring-opening reaction at the imidazolium sites present in 1^4+^. While not hitherto exploited for the purposes of post-synthetic receptor modification, ring-opening reactions involving imidazolium species (*cf.*Scheme 2) have been known for at least 25 years.^23–26^ However, typically hydrolysis of common imidazole-2-ylidenes requires strongly basic aqueous media.^26^ We were thus keen to explore whether the unique macrocyclic nature of 1^4+^·4PF_6_^−^ would allow viable PSM imidazolium ring-opening conversions to be carried out under milder and hence more accessible conditions.

Initial experiments in pursuit of this goal were carried out in a 1 : 1 mixture of acetonitrile and an aqueous medium, which consisted of water containing varying concentrations of NH_3_·H_2_O (25–28% by mass). The effects of base concentration, temperature, and reaction time on the ring-opening reaction of 1^4+^·4PF_6_^−^ were then explored. It was found that macrocycle 1^4+^ underwent essentially complete conversion in 45 h to a mixture of isomeric imidazolium ring-opened products. This occurred even when the pH of the initial solvent system was as low as 11 at 298 K (*cf.* ESI†). This facility stands in contrast to what is seen in the case of the non-macrocyclic control, imidazole-2-ylidene, where even at a pH as high as 13, no appreciable hydrolysis was reported to take place at room temperature.^26^ For preparative PSM conversions involving 1^4+^, a pH of 11.6 at 313 K and a reaction time of 24 h were employed.^27^

The preparative reactions were worked up easily by using compressed air to blow off the residual acetonitrile and other volatiles. The light yellow precipitate obtained from the remaining aqueous layer was then collected and analyzed. ^1^H NMR spectroscopic analyses led us to conclude that the precipitate obtained in this way consisted of a mixture of related products (*cf.* ESI† for spectra). An effort was made to purify and characterize these products as described below.

To determine the structures of the ring-opened products, ^1^H NMR and diffusion ordered spectroscopic (DOSY) analyses were carried out on the crude products obtained from the preparative-scale reaction outlined above. It was found that the proton signals of the products had essentially the same diffusion coefficients as the starting tetracationic macrocycle (*D* = 1.1 × 10^−10^ m^2^ s^−1^ in both cases). On this basis, we conclude that the crude product or products obtained after treatment of 1^4+^·4PF_6_^−^ with aqueous base and the starting macrocycle (*i.e.*, 1^4+^) have very similar molecular weights, shapes, and polarities.

**Scheme 3:**
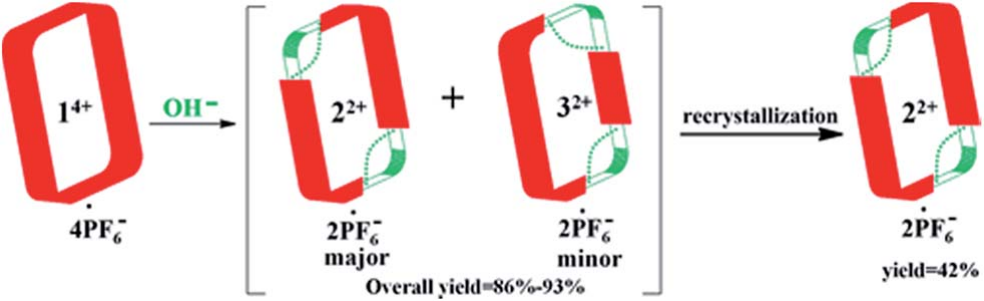
Schematic representation of the imidazolium ring-opening reaction that allows for PSM of macrocycle 1^4+^. Also shown are the major and minor regioisomeric products (2^2+^ and 3^2+^, respectively) that result from this process.

In an effort to purify the crude material, it was subjected to recrystallization using a mixture of acetonitrile and dioxane (2 : 1, v/v). This allowed isolation of pure *trans*-cyclo[2]((*Z*)-*N*-(2-((6-(1*H*-imidazol-1-yl)pyridin-2-yl)amino)vinyl)formamide)[2](1,4-bi-methylbenzene) hexafluorophosphate (*i.e.*, 2^2+^·2PF_6_^−^), as inferred from ^1^H NMR spectroscopic analysis (*cf.*Scheme 3 and ESI†). This particular imidazolium ring-opened product was isolated in an overall yield of 42%. Further support for the formation of 2^2+^ as its PF_6_^−^ salt came from mass spectrometric studies using ESI-MS. These analyses revealed a peak corresponding to [2^2+^·PF_6_^−^]^+^ (*m*/*z* = 809.2657) in the gas phase.

Analysis of the residual material *via*^1^H NMR and DOSY spectroscopy led to the conclusion that there was a second minor product present in the original crude product. This minor product was identified as 3^2+^·2PF_6_^−^ on the basis of a single crystal X-ray diffraction analysis and ESI-MS studies wherein a peak corresponding to [3^2+^·PF_6_^−^]^+^ (*m*/*z* = 809.2670) was seen. The overall yield of crude product (mixture of 2^2+^·2PF_6_^−^ and 3^2+^·2PF_6_^−^) was 86–93%, with the ratio of major to minor isomer being roughly 3 : 1 based on ^1^H NMR spectral integrations. Unfortunately, it proved difficult to obtain significant quantities of pure 3^2+^·2PF_6_^−^*via* recrystallization or high performance liquid chromatography (HPLC).^28^ This difficulty is ascribed to the fact that 3^2+^·2PF_6_^−^ has physical properties that are similar to those of the other regioisomer (*i.e.*, 2^2+^·2PF_6_^−^).

**Scheme 4:**
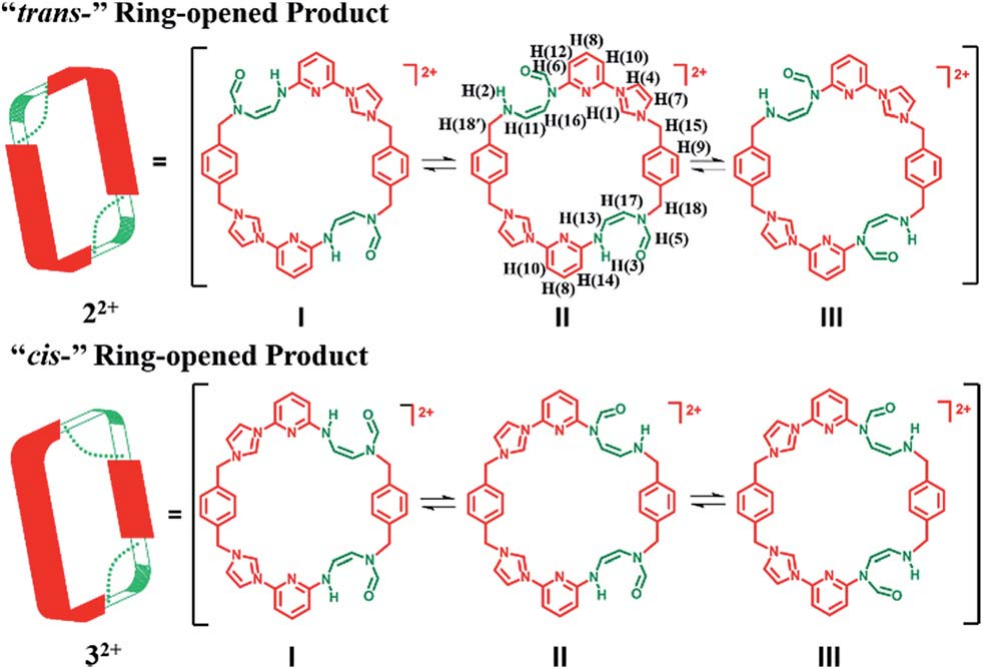
Schematic representation of three interconvertible isomers of 2^2+^ and 3^2+^, respectively. In DMSO-*d*_6_ solution at 300 K, two of these isomers (I and III) dominate in the case of both ring-opened species 2^2+^ and 3^2+^ and are found in a *ca.* 1 : 1 ratio. In DMF-*d*_7_ solution at 278 K, all three isomers coexist and in a *ca.* 1 : 2 : 1 ratio (I : II : III).

Initial characterization of 2^2+^·2PF_6_^−^ was carried out in DMSO-*d*_6_ using a variety of techniques, including ^1^H NMR, ^13^C NMR, heteronuclear singular quantum correlation (HSQC), 2D correlation (COSY), one-dimensional and two-dimensional nuclear Överhauser effect (1D NOE and NOESY) spectroscopies. The ^1^H NMR spectrum proved complex, leading us to suggest that 2^2+^ is highly flexible in solution (as inferred from the observation of correlations between H(1), H(4) and H(10)) and exist in several different inter converting configurational forms. Further evidence of this proposed intramolecular conversion came from NOESY and 1D NOE spectral studies. Here, evidence of rapid exchange between the signals of H(5) and H(6), namely the proton signals on the formoxyl group, was observed. On this basis we propose that upon ring opening, macrocycle 2^2+^ undergoes rapidly structural interconversion wherein the formoxyl group and the neighbouring amino moiety exchange, as shown in Scheme 4. Additional insights into this proposed intramolecular conversion came from temperature dependent ^1^H NMR and ^13^C NMR spectroscopic analyses carried out at 278 K and 373 K, HSQC analyses, as well as ^1^H,^15^N-heteronuclear multiple bond correlation (^1^H,^15^N-HMBC), COSY, and NOESY studies, all of which were carried out in DMF-*d*_7_ (*cf.* ESI†). In this solvent system the signal ascribed to H(1) on 2^2+^ is split into four peaks at 278 K, a finding that is consistent with the intramolecular conversion shown in Scheme 4. Additional evidence in support of this conclusion came from a ^13^C NMR spectroscopic study carried out at 278 K, wherein more than 50 signals were observed. The ^1^H,^15^N-HMBC spectrum was also recorded. Here, 7 distinct ^15^N signals were seen, a finding that provides additional support for the proposed intramolecular conversion of 2^2+^. Furthermore, the ^1^H and ^13^C signals ascribed to the differing isomers of 2^2+^ become degenerate at 373 K (*cf.* ESI†). This is consistent with higher temperatures serving to accelerate the intramolecular conversions involving the various isomeric forms of 2^2+^.

Support for the above conversion process involving an exchange between isomers, rather than an equilibrium between two or more chemically distinct products came from electrospray ionization mass spectrometry (ESI-MS), which revealed a signal with a *m*/*z* = 663.2934 ([(2^2+^ − H)]^+^˙) without evidence of interfering species (*cf.* ESI†). A single crystal X-ray diffraction analysis of 2^2+^·2PF_6_^−^ was also carried out, as discussed below.

In the case of the minor product, 3^2+^·2PF_6_^−^, it was not possible to isolate enough material to successfully conduct ^1^H NMR spectroscopic analysis. However using the crude material, and accounting for the signals for 2^2+^·2PF_6_^−^, it was inferred that 3^2+^ displays at least 13 signals in the ^1^H NMR spectrum recorded in DMSO-*d*_6_ at 300 K (*cf.* ESI†). This leads us to suggest that interconversion between the formoxyl group isomers also takes place within macrocycle 3^2+^, as illustrated in Scheme 4.

**Scheme 5:**
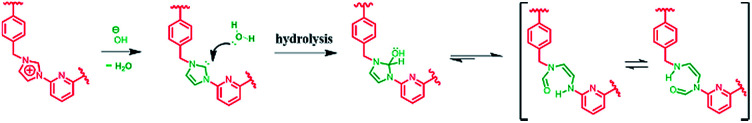
Schematic representation of the hydrolysis of the imidazole-2-ylidene moieties in 1^4+^ that occurs in mild basic aqueous solution (pH = 11.6).

As a control study, 1,3-dimethyl-1*H*-imidazolium (studied as its respective PF_6_^−^ salt) was tested under the same reaction conditions used for the preparative-scale PSM of 1^4+^·4PF_6_^−^. In this case, no evidence of imidazolium ring opened products was observed (*cf.* ESI†). This result, which is in accord with what was expected based on the literature,^26^ leads us to suggest that the macrocyclic structure of 1^4+^ with its relatively enhanced anion binding properties serves to activate the constituent imidazolium moieties and increase their propensity to undergo hydrolysis (*cf.*Scheme 5).

**Fig. 1:**
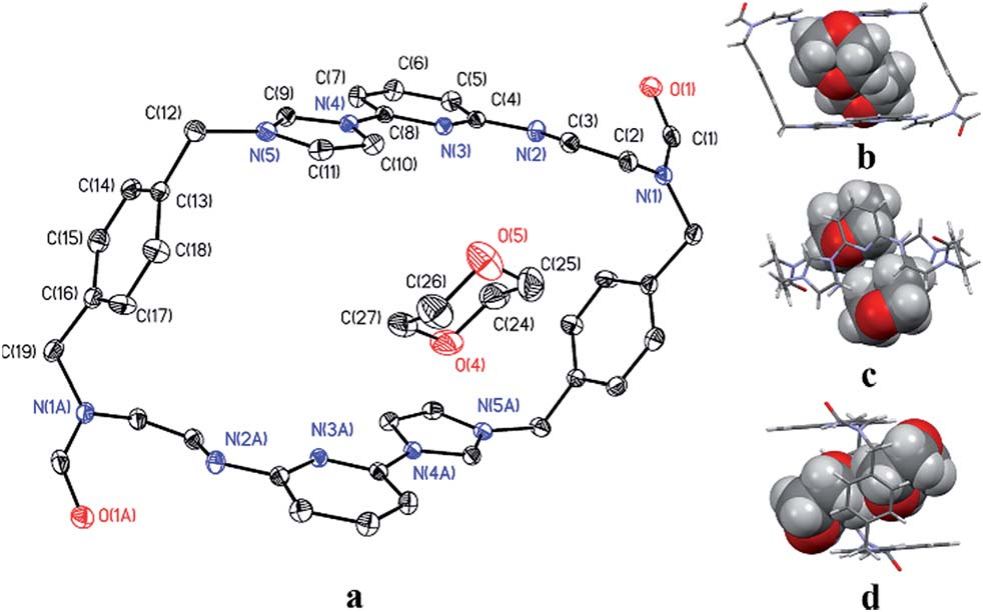
“Box-like” structure of 2^2+^ as seen in the single crystal X-ray diffraction analysis of [2^2+^·2PF_6_^−^·4dioxane] showing (a) one of the four dioxane molecules present in the resulting cavity. Views of the structure showing the macrocycle framework and the dioxane cosolvent in space-filling form as seen from the top (b), side (c) and front (d). Some of the counter ions and solvent molecules have been omitted for clarity.

Single crystals of 2^2+^·2PF_6_^−^ suitable for X-ray diffraction analysis were grown from two different solvent systems, namely (1) acetonitrile/dixoane (2 : 1, v/v) and (2) a mixed solution of water/acetonitrile (1 : 1, v/v) *via* slow evaporation. This yielded two independent sets of single crystals. Analysis of the single crystals obtained from the first crystallization method revealed a structure wherein macrocycle 2^2+^ is arranged in a “box-like” conformation around molecules of dioxane (*i.e.*, [2^2+^·2PF_6_^−^·4dioxane]; *cf.*Fig. 1). The four bound dioxane cosolvent molecules appear to be stabilized by weak non-covalent O⋯π interactions, as inferred from the metric parameters (*cf.* ESI†). The width of the box was determined to be 5.814 Å with a length of 11.425 Å. The length of 2^2+^ in this structure is larger than seen in the structure of free 1^4+^ (7.699 or 10.136 Å in “complete chair” or “partial chair” conformation, respectively);^22*a*,*b*^ however, the two macrocycles have approximately the same width (*cf.*Fig. 1 and the ESI†).

**Fig. 2:**
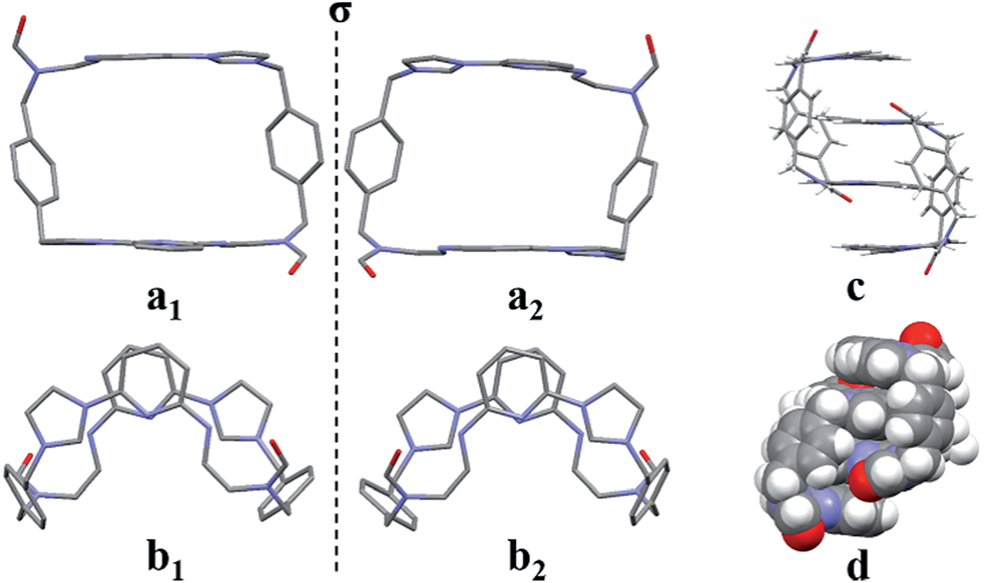
Top (a_1_ and a_2_) and side (b_1_ and b_2_) views of the single crystal X-ray structure of [2^2+^·2PF_6_^−^·CH_3_CN·H_2_O] showing the enantiomeric pair present in the crystal. Stick (c) and space-filling (d) views of these clip-like enantiomeric pairs. Counter ions and solvent molecules have been omitted for clarity.

The second single crystal of macrocycle 2^2+^ isolated and used for analysis contained a single acetonitrile cosolvent molecule per macrocycle, as well as two hexafluorophosphate counter anions (*i.e.*, [2^2+^·2PF_6_^−^·CH_3_CN·H_2_O]). In the resulting structure, macrocycle 2^2+^ is seen to adopt a “clip-like” conformation around the acetonitrile cosolvent (*cf.*Fig. 2 and ESI†). It exists in the form of two enantiomeric conformations that are paired in the overall structure. As part of this pairing, two neighbouring molecules of 2^2+^ are bound to each other *via* presumed π⋯π donor–acceptor interactions involving the stacked aromatic planes of ((*Z*)-*N*-(2-((6-(1*H*-imidazol-1-yl)pyridin-2-yl)amino)vinyl)formamide). The result is the formation of head-to-tail racemic dimer (*i.e.*, 2_2_), which contains a centre of symmetry (*cf.*Fig. 2). This second structure of 2^2+^ was also found to differ significantly from that of 1^4+^.^22*a*^ Specifically, the width of the top of 2^2+^ in its clip conformation is 7.485 Å, which is significantly smaller than what was observed in the case of 1^4+^ in a structure wherein it also adopts a clip-like conformation (9.370 Å; *cf.* ESI†).

The two different conformations observed in the single crystal structures of 2^2+^ were taken as evidence that this imidazolium ring-opened system is relatively flexible. The flexibility of 2^2+^, which was originally inferred from the spectroscopic analyses discussed above, is reminiscent of Alcalde's dicationic imidazolium-based cyclophanes.^29^ In this structure, the carbonyl groups are located on the nitrogen atoms of the two ring-opened segments, which are linked *via* methylene units.

Even though it proved challenging to isolate preparative quantities of 3^2+^·2PF_6_^−^, single crystals of [3^2+^·2PF_6_^−^·CH_3_CN] suitable for X-ray diffraction analysis could be isolated after the bulk of the 2^2+^·2PF_6_^−^ product was removed by recrystallization. The single crystal used for structural analysis was obtained by running the ring opening reaction under the preparative conditions described above, subjecting the resulting crude product to recrystallization from dioxane/acetonitrile, collecting the solid material that consisted predominantly of 2^2+^·2PF_6_^−^, removing the solvent, redissolving the remaining oil in a mixture of acetonitrile/water (1 : 1, v/v), and then subjecting the resulting solution to slow evaporation. X-ray diffraction structural analysis revealed that 3^2+^ was the *cis*-isomer of 2^2+^.

**Fig. 3:**
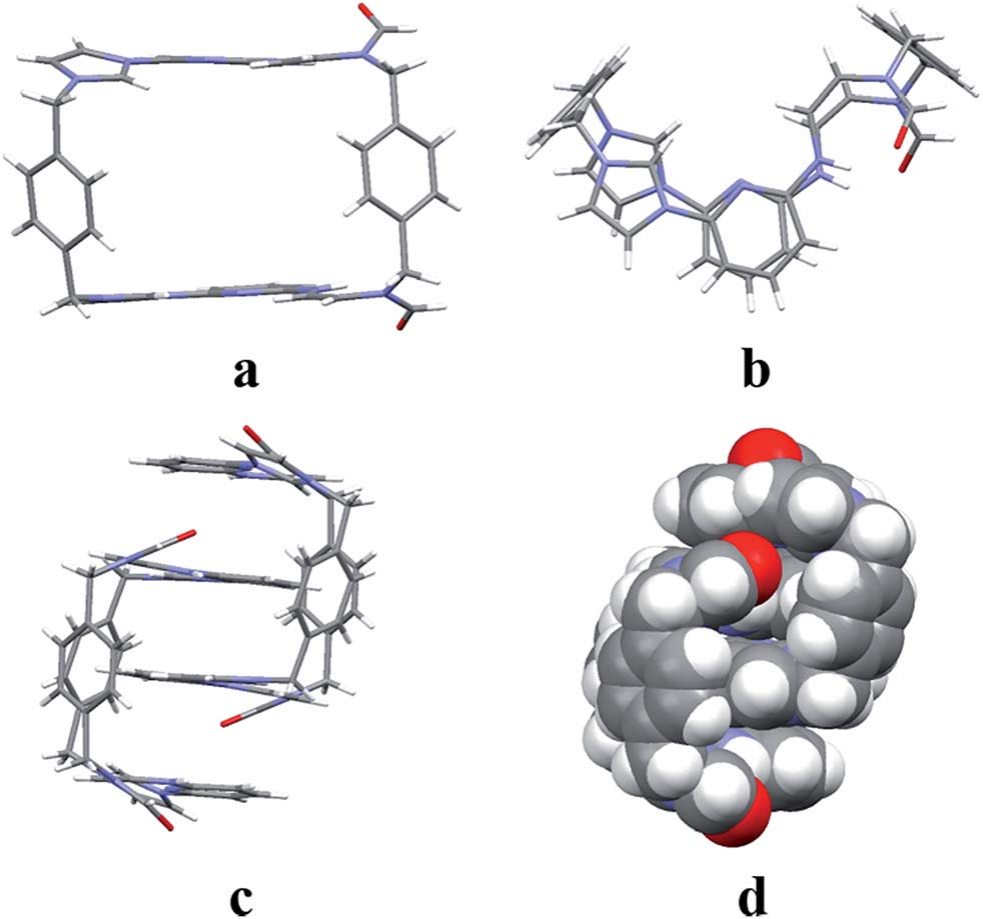
Single crystal X-ray structure of [3^2+^·2PF_6_^−^·CH_3_CN] as viewed from the top (a) and side (b), as well as the clip-like dimer which is shown in stick (c) and space-filling forms (d). Some of the counter ions and solvent molecules have been omitted for clarity.

In analogy to what was seen for [2^2+^·2PF_6_^−^·CH_3_CN·H_2_O], it was found that in the solid state the dicationic macrocycle, 3^2+^, adopts a “clip” conformation containing a plane of symmetry. As was true for the acetonitrile adduct of 2^2+^, a head-to-tail dimer arrangement was seen (*i.e.*3_2_). This dimer is presumably stabilized by multiple π⋯π donor–acceptor interactions as reflected in the short distances (≤3.5 Å) between the neighbouring planes (*cf.*Fig. 3 and ESI†).

The above studies provide support for the suggestion that the imidazolium ring-opening PSM reaction that serves to convert macrocycle 1^4+^ into 2^2+^ also serves to alter the structure of the host. It was expected that this would translate into a difference in molecular recognition properties. To test this hypothesis, we chose for study anionic guests that had been previously investigated with 1^4+^, namely 2,6-naphthalenedicarboxylate (4), 2,6-naphthalenedisulfonate (5), and 1,5-naphthalenedisulfonate (6).^22*b*,*c*^ As was true for this earlier work, the anions were studied in the form of their tetramethylammonium (TMA^+^) salts and all titrations were carried out in DMSO-*d*_6_.

Initially, NMR spectroscopic analyses were used to investigate the host–guest interactions between 2^2+^ and 4. Upon titration of 2^2+^ with 4, distinct shifts in the ^1^H NMR spectral features associated with both the host and the guest species were observed. The greatest changes were seen for the imidazole C–H and N–H resonances. Job plots were constructed and revealed a maximum value of 0.5 (for the ratio [H]/([H] + [G])), as would be expected for a 1 : 1 (H : G) binding stoichiometry. Fitting the ^1^H NMR spectral titration data to a 1 : 1 binding profile, allowed association constants of *K*_a_ = (2.0 ± 0.1) × 10^2^ M^−1^ to be calculated for the formation of a formally neutral host–guest complex (*i.e.*, [2^2+^·4]).

**Scheme 6:**
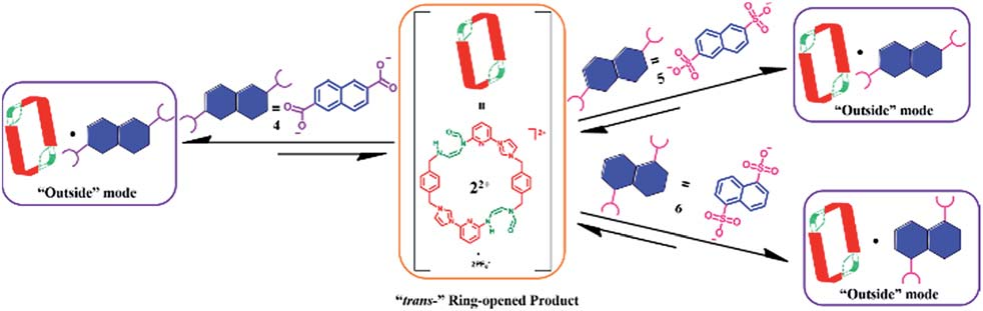
Schematic representation of the interactions between 2^2+^ and guest species 4, 5, and 6, as inferred from ^1^H NMR spectroscopic analyses carried out in DMSO-*d*_6_.

To understand better the nature of this presumed 1 : 1 complex, 1D-NOE and NOESY NMR spectroscopic studies were carried out. These analyses revealed no correlations between the protons on 2^2+^ and those on 4. This finding is consistent with guest anion 4 being bound outside the central cavity of macrocycle 2^2+^ (*cf.*Scheme 6). Further support for this 1 : 1 binding mode came from ESI-MS studies, wherein a peak corresponding to [2^2+^ + 4 + H]^+^˙ (*m*/*z* = 879.3366) was observed (*cf.* ESI†). The proposed outside binding mode in [2^2+^·4] is distinctly different from the interpenetrated structure observed when 1^4+^ is exposed to 4.^22*b*^ This difference highlights the effect that the present PSM can have on the host–guest recognition properties of ostensibly related cationic receptor systems.

The molecular recognition features of 2^2+^ were explored further using 5 as a guest (*cf.*Scheme 6). As in the case of 4, direct titration of 2^2+^ with 5 resulted in chemical shifts in the ^1^H NMR spectral features associated with both the host and the guest. As was seen with 4, a Job-plot analysis of the ^1^H NMR spectral data revealed a maximum at *ca.* 0.5 ([H]/([H] + [G])). Such a finding is consistent with a 1 : 1 (H : G) binding stoichiometry. More detailed analysis of the ^1^H NMR spectral data, led to the conclusion that a 1 : 1 ([2^2+^·5]) complex was being formed during the course of the titration. A binding constant of *K*_a_ = (1.7 ± 0.2) × 10^2^ M^−1^ was calculated for the formation of [2^2+^·5]. Separate NOESY and 1D NOE spectroscopic analyses provided support for the conclusion that in this 1 : 1 complex guest molecule 5 is located outside of the macrocyclic core (*cf.* ESI†). ESI-MS analyses revealed *inter alia* peaks corresponding to [2^2+^ + 5 − 6H]^+^˙ (*m*/*z* = 944.2182) in the gas phase. Again, this binding behaviour was very different from that seen when 1^4+^ was used as a receptor. In this case 1  : 1 pseudorotaxane complexes are seen with 5 in DMSO-*d*_6_.^22*c*^

The interactions between 2^2+^ and 6 were also investigated. Studies analogous to those performed in the case of 4 and 5 revealed the formation of a 1 : 1 (H : G) complex (*cf.* ESI†). Again, 1D-NOE and NOESY analyses revealed no correlation between protons on host 2^2+^ and guest 6, leading us to conclude that guest binding takes place outside of the central cavity of 2^2+^ (*cf.*Scheme 6). In this case, and in contrast to what is observed for 4 and 5 there is direct analogy to the binding behavior seen with 1^4+^, which also interacts with 6*via* an outside binding mode. However, as might be expected given the charge disparity, complex [1^4+^·6]^2+^ displayed greater thermodynamic stability than [2^2+^·6] as inferred from the associated *K*_a_ values ((1.0 ± 0.1) × 10^3^ M ^−1^ for [1^4+^·6]^2+^ as noted previously,^22*c*^ and (4.0 ± 0.5) × 10^2^ M^−1^ for [2^2+^·6]; *cf.* ESI†). A diffusion ordered spectroscopic (DOSY) study (DMSO-*d*_6_) revealed that all of the proton signals located on 2^2+^ and 6 have similar diffusion rates in the representative solution state mixtures (*cf.* ESI†). Evidence for the formation of the 1 : 1 species [2^2+^·6] was again found in the gas phase *via* ESI-MS analysis. Specifically, a peak corresponding to [2^2+^ + 6 + Na]^+^˙ (*m*/*z* = 973.2519) was observed (Table 1).

**Table 1:** Summary of the complexes formed between 2^2+^·2PF_6_^−^, 1^4+^·4PF_6_^−^ and dianions (4, 5, and 6)

Guest	1^4+^			2^2+^		
	Binding mode	Stoichiometric ratio/(H : G)	Association constants (*K*_a_)/M^−1^	Binding mode	Stoichiometric ratio/(H : G)	Association constants (*K*_a_)/M^−1^
2,6-Naphthalene dicarboxylate (4)	Pseudo rotaxane	1 : 1	(3.5 ± 0.2) × 10^4^	“Outside”	1 : 1	(2.0 ± 0.1) × 10^2^
2,6-Naphthalene disulfonate (5)	Pseudo rotaxane	1 : 1	(1.6 ± 0.1) × 10^3^	“Outside”	1 : 1	(1.7 ± 0.2) × 10^2^
1,5-Naphthalene disulfonate (6)	“Outside”	1 : 1	(1.0 ± 0.1) × 10^3^	“Outside”	1 : 1	(4.0 ± 0.5) × 10^2^

Based on the above studies, we conclude that the recognition features of 1^4+^ and its PSM derivative 2^2+^ differ in (1) the basic binding mode and (2) the binding affinities, with those of macrocycle 2^2+^ being weaker. We postulate that these differences result directly from the structural changes induced *via* imidazolium ring-opening, which results in a larger cavity, lower net positive charge, and weaker hydrogen bonding interactions in the case of 2^2+^ as compared to 1^4+^.

To provide further support for these hypotheses, we set out to isolate single crystals of the molecular complexes produced from 2^2+^ and the test anions. Single crystals suitable for X-ray diffraction analysis were obtained in the case of [2^2+^·5·5H_2_O] and [2^2+^·6·2.5H_2_O]. The crystals were obtained *via* slow diffusion using a three-layer solution setup. Specifically, 1.0 molar equiv. of 5 or 6, and 2.0 molar equiv. of TMA^+^·OH^−^ (based on 2^2+^·2PF_6_^−^) were dissolved in water and placed in a small tube. A mixture of DMF and water (1 : 1, v/v) was added as the second layer, and 2^2+^·2PF_6_^−^ (1.0 molar equiv.) dissolved in CH_3_CN, DMF, and water (1 : 1 : 1, v/v/v) was then added as the third layer.

**Fig. 4:**
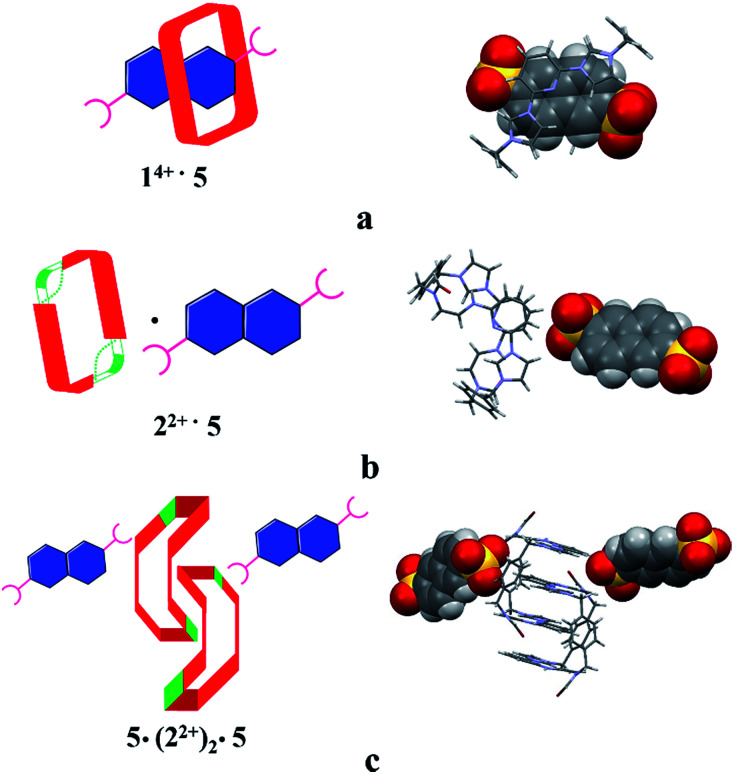
Schematic views and single crystal X-ray structures of (a) [1^4+^·5_2_·7.5H_2_O],^22*c*^ (b) [2^2+^·5·5H_2_O], and (c) 5·(2^2+^)_2_·5. Most counter ions and solvent molecules have been omitted for clarity.

Structural analysis in the case of [2^2+^·5·5H_2_O] (*cf.*Fig. 4), revealed that dianion 5 binds to the outside of macrocycle 2^2+^ in the solid state, as was inferred from the solution NMR spectroscopic analyses discussed above. Host 2^2+^ and guest 5 are bound primarily *via* intermolecular C–H⋯O hydrogen bonds characterized by relatively short C–O separations (≤3.63 Å). In the solid state, the complex exists as a dimeric species 5·(2^2+^)_2_·5 stabilized *via* presumed π⋯π donor–acceptor interactions (*cf.*Fig. 4 and the ESI†). This solid state binding behaviour is distinctly different from what is seen in the case of 1^4+^ and 5 which aggregate to form pseudorotaxane units (*i.e.*, [1^4+^·5_2_·7.5H_2_O]), as noted in previous studies.^22*c*^

**Fig. 5:**
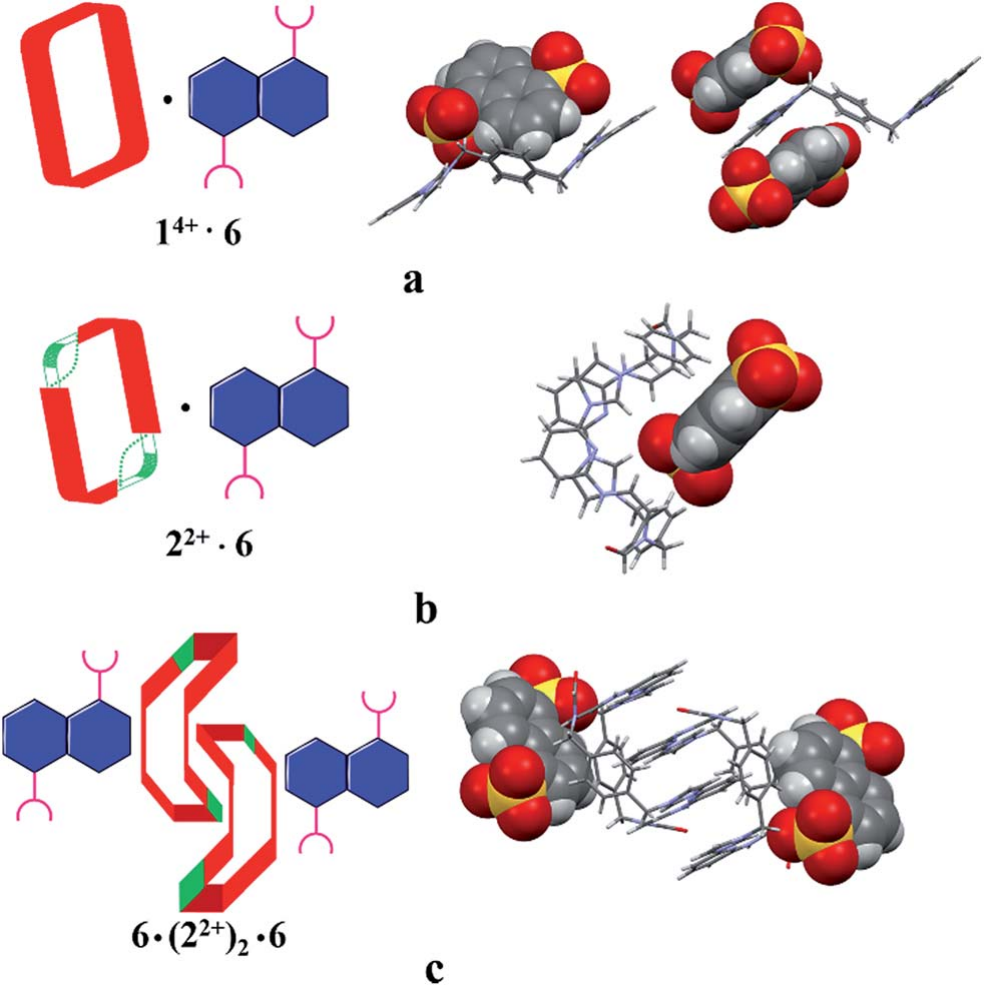
Schematic views and single crystal X-ray structure of (a) [1^4+^·6_2_·6H_2_O], [1^4+^·6_2_·14H_2_O],^22*c*^ (b) [2^2+^·6·2.5H_2_O] and (c) 6·(2^2+^)_2_·6. Some of the counter ions and solvent molecules have been omitted for clarity.

The single-crystal X-ray structure of [2^2+^·6·2.5H_2_O] (*cf.*Fig. 5) also served to confirm the inference drawn from the solution phase analyses (*vide supra*), namely that the dianionic guest species is bound to the periphery of macrocycle 2^2+^. In this case the binding mode is similar to what was observed previously in the case of 1^4+^, where outside binding is also seen (*i.e.*, [1^4+^·6]^2+^ in [1^4+^·6_2_·6H_2_O] or [1^4+^·6_2_·14H_2_O]) (*cf.*Fig. 5). However, different conformations are seen for the cationic receptors. In the case of [2^2+^·6·2.5H_2_O], the macrocycle adopts a “clip” conformation where as a “chair” mode was seen in the case of [1^4+^·6]^2+^ in [1^4+^·6_2_·6H_2_O] or [1^4+^·6_2_·14H_2_O] (*cf.*Fig. 5). The primary stabilizing interactions between 2^2+^ and 6 are intermolecular C–H⋯O hydrogen bonds, as inferred from the short C–O distances (≤3.60 Å). Also present are π⋯π donor–acceptor interactions characterized by the short separation (≤3.8 Å) between the naphthalene ring on 6 and the neighbor benzene plane on 2^2+^ (*cf.* ESI†). An overall head-to-tail racemic dimer (*i.e.*2_2_) with a center of symmetry is seen in the solid state structure of [2^2+^·6·2.5H_2_O] (*cf.*Fig. 5 and ESI† for further details). This mirrors what was seen for [2^2+^·5·5H_2_O] as noted above.

To test whether the present OH^−^ anion-promoted ring-opening of 1^4+^ would complement the previously observed acid-induced dethreading seen for pseudorotaxanes built up from this core, both [1^4+^·4] and [1^4+^·5] (obtained from a mixture of 1^4+^·4PF_6_^−^ and 1 molar equiv. of either 4 or 5*in situ*) were subject to treatment with OH^−^. The correlation between protons on the macrocycle as well as the guest species were monitored *via* 1D-NOE while irradiating the protons on anionic guest 4 or 5. It was found that over the course of the ring-opening process the correlated signals disappear. This was taken as evidence that the pseudorotaxne structures, *i.e.*, [1^4+^·4] or [1^4+^·5], underwent disassembly under the aforementioned reaction conditions. Further proof of the proposed dethreading of anion 5 came from a single-crystal diffraction study involving [2^2+^·5·5H_2_O] (*cf.*Fig. 4b). The single crystals used for this analysis were obtained from the contents of an NMR tube that was left standing for 12 h after the initial reaction between [1^4+^·5] and OH^−^. Identical crystals were obtained when 2^2+^ was mixed with 5 and subject to slow diffusion crystallization as detailed above.

As noted above, we previously reported that treatment of [1^4+^·4] with a proton source leads to formation of 1^4+^ and 2H^+^·4. Taken in conjunction with the previous findings, the present results serve to highlight that the fact that pseudorotaxane [1^4+^·4] is only stable as a mechanically interlocked species over a limited effective pH range; thus mimicking many biological systems, which only maintain their unique structures and bioactivity at certain well-defined pH values.

## Conclusions

In summary, a new post-synthetic modification strategy is described that may provide a facile means for modulating the properties of cationic macrocycles. This strategy has been applied to the tetraimidazolium Texas-sized box, 1^4+^ to give two regioisomeric imidazolim ring-opened products, 2^2+^·2PF_6_^−^ and 3^2+^·2PF_6_^−^. The *trans* isomer (*i.e.*, 2^2+^·2PF_6_^−^) is the dominant product and could be obtained on a preparative scale. It displayed outside binding or only weak ion pairing when tested with the dianionic substrates 4 and 5. Its recognition behaviour thus proved to be distinct from its macrocyclic precursor (1^4+^), a tetracationic systems that supports the formation of interpenetrated pseudorotaxane complexes with these same anionic guests. Moreover, the OH^−^ ring-opening process can be used to irreversibly disassemble the interpenetrated structures [1^4+^·4] and [1^4+^·5].

**Fig. 6:**
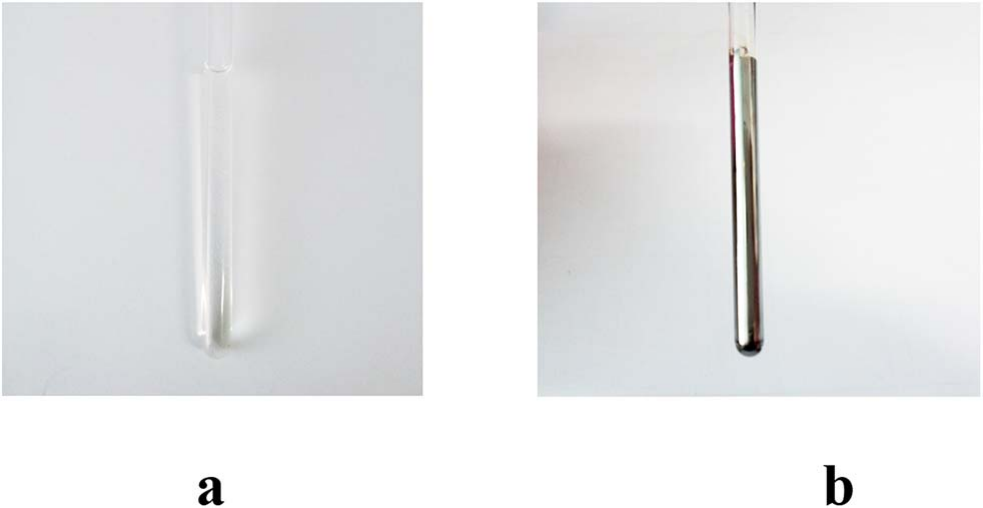
Photos of 2^2+^·2PF_6_^−^ (4.5 mM L^−1^) taken 10 min (a) and 10 h (b) after treatment with 4 molar equiv. of AgNO_3_ in a mixture of CD_3_CN, D_2_O, and ND_3_·D_2_O (25–28% by mass) (1/0.4/0.007, v/v/v) at 323 K.

The unique nature of the PSM reaction reported here, which includes its relatively high yield, the mild reaction conditions it requires, and an ability to obtain differing regioisomers leads us to suggest that this simple yet versatile approach could prove to be of general utility and readily applied to other imidazolium-based receptor systems. More broadly, new methods that allow for the post-synthetic modification of known receptor systems are likely to allow for the exploration of new recognition and reactivity chemistry (*e.g.*, the reaction of the –NH and/or –CHO), just as it has in the parallel area of MOFs. Indeed, in preliminary work, we have found that treatment of 2^2+^·2PF_6_ with AgNO_3_ leads to formation of a sliver mirror (*cf.*Fig. 6).^30^

## Supplementary Material

SC-007-C5SC04860E-s001

SC-007-C5SC04860E-s002
