# Supplementary material for: Post-synthetic modification of a macrocyclic receptor via regioselective imidazolium ring-opening
Source: Chem Sci. 2016 Mar 9;7(7):4148–57. doi: 10.1039/c5sc04860e (PMC6014093; doi:10.1039/c5sc04860e)
Supplement: SC-007-C5SC04860E-s001 [file SC-007-C5SC04860E-s001.pdf]

Electronic Supplementary Material (ESI) for Chemical Science.
This journal is © The Royal Society of Chemistry 2016

Supporting Information For:

Post-synthetic Modification of a Macrocyclic Receptor via Regioselective Imidazolium Ring-opening

Jia Shang,[a] Brett M. Rambo,[b] Xiang Hao,[c] Jun-Feng Xiang,[c] Han-Yuan Gong,[*,a] and Jonathan L. Sessler[*,b,d]

[a] College of Chemistry, Beijing Normal University, Xinjiekouwaidajie 19, Beijing 100875, P. R. China;

[b] Department of Chemistry, The University of Texas at Austin, 105 East 24th Street, Stop A5300, Austin, Texas 78712-1224, United States;

[c] Institute of Chemistry, Chinese Academy of Sciences, Zhongguancunbeiyijie 2, Beijing 100190, P. R. China;

[d] Department of Chemistry, Shanghai University, Shanghai 200444, China

*e-mail: hanyuangong@bnu.edu.cn; sessler@cm.utexas.edu

Materials and Methods

Section S1: *General considerations (pp. S3-S4)*

Section S2: *Synthesis, characterization, and conformational study of [**2**$^{2+}$·2PF$_6^-$] and [**3**$^{2+}$·2PF$_6^-$] in solution (pp. S5-S33)*

Section S3: *Single crystal X-ray diffraction analysis of [**2**$^{2+}$·2PF$_6^-$·4dioxane], [**2**$^{2+}$·2PF$_6^-$·CH$_3$CN·H$_2$O] and [**3**$^{2+}$·2PF$_6^-$·CH$_3$CN] (pp. S34-S39)*

Section S4: *Solution binding studies and characterization of the host-guest complexes formed between [**2**$^{2+}$·2PF$_6^-$] and dianionic guests 2,6-naphthalenedicarboxylate (**4**), 2,6-naphthalenedisulfonate (**5**) or 1,5-naphthalenedisulfonate (**6**) (pp. S40-S54)*

Section S5: *Single crystal X-ray analysis of the complexes formed between **2**$^{2+}$ and dianionic guest **5** or **6** (pp. S55-S59)*

Section S6: *Mass spectrometric analysis of the complexes formed between **2**$^{2+}$ and dianionic guest **4**, **5** or **6** (p. S60-S63)*

Section S7: *Solution binding studies and characterization of the OH$^-$ ring-opening process leading to the irreversible disassembly of the interpenetrated structures [**1**$^{4+}$·**4**] and [**1**$^{4+}$·**5**] (pp. S64-S69)*

Section S7: *Comparative analysis of the conformation of [**2**$^{2+}$·2PF$_6^-$] and [**1**$^{4+}$·4PF$_6^-$], and the associated complexes formed with anionic guest **4**, **5**, or **6** (pp. S70-S72)*

Section S8: *References (pp. S73)*

Section S1: *General considerations*

All solvents were dried before use according to standard protocols.[1] For this study, all reagents were purchased commercially (Aldrich, Acros, or Fisher) and used without further purification. Deuterated solvents were purchased from Cambridge Isotope Laboratory (Andover, MA). NMR spectra were recorded on Bruker AVANCE III 400, AVANCE III 500WB or AVANCE 600 spectrometers. The ^{1}H and ^{13}C NMR chemical shifts are reported relative to residual solvent signals (^{1}H: DMSO-d_6 at 2.50 ppm).[2] ESI-MS data was acquired by infusion on a Thermo LTQ-XL linear ion trap mass spectrometer operating in positive ion mode.

All single crystals used to obtain the X-ray diffraction structures grew as colorless prisms or needles, or yellow prisms. The .cif documents are available as separate supporting information files, which provide details regarding the specific crystals used for the analysis, along with the structure in question. Diffraction grade crystals were obtained *via* slow evaporation or liquid-liquid diffusion from solution in a mixture of water/acetonitrile/DMF or water/acetonitrile or dioxane/acetonitrile as described below.

The specific liquid-liquid diffusion conditions for crystal cultivation are shown below: a solvent mixture (DMF/water, 1/1, v/v; 2 mL), used as the second layer, was added to a first layer of guest **5** or **6** in water (0.400 mL of a 0.01 M solution). Then, 1 mL of $\mathbf{2}^{2+}$ (0.05 M) in a mixture of DMF/acetonitrile/water (1/1/1, v/v/v; 1 mL) was added as the third layer to the clear solution. The anion guest was produced from $2H^{+}$·**5** or $2H^{+}$·**6** in situ *via* the addition of two molar equiv. of tetramethylammonium hydroxide ($TMA^{+}\cdot OH^{-}$) such that tetramethylammonium was the counter cation.

The crystals used for single crystal analyses were cut from clusters of the corresponding crystals and had the approximate dimensions given in the .cif documents. The data were collected on a D8 Venture, Rigaku Mercury2 (2x2 bin mode) or Saturn724+ (2x2 bin mode) CCD diffractometers using a graphite monochromator with *MoKα* radiation (λ = 0.71073 Å). Data reduction was performed using CrystalClear or APEX II. The structures were solved by

direct methods using SIR97[3] and refined by full-matrix least-squares on F^2 with anisotropic displacement parameters for the non-H atoms using SHELXL-97.[4] The hydrogen atoms were calculated in idealized positions with isotropic displacement parameters set to 1.2 x Ueq of the attached atom (1.5 x Ueq for methyl hydrogen atoms). The utility ROTAX[5] in the program WinGX[6] was used to look for possible twins. R(F), Rw(F2) and the goodness of fit, S, are given below and in the .cif documents. All ellipsoid figures were generated using SHELXTL/PC.[7] Tables of positional and thermal parameters, bond lengths and angles, torsion angles, figures and lists of observed and calculated structure factors are located in the .cif documents available from the Cambridge Crystallographic Centre and may be obtained by quoting the CCDC ref. numbers 1433722, 1433723, 1433724, 1433725 and 1433726. The documents also contain details of the crystal data, data collection, and structure refinement for each structure.

Section S2: *Synthesis, characterization, and conformational study of [$\mathbf{2}^{2+}\cdot 2PF_6^-$] and [$\mathbf{3}^{2+}\cdot 2PF_6^-$] in solution*

General procedure for the synthesis of [$2^{2+}\cdot 2PF_6^-$] and [$3^{2+}\cdot 2PF_6^-$]

In a typical procedure, a 25ml small vial was charged with 50 mg (0.041 mmol) $\mathbf{1}^{4+}\cdot 4PF_6^-$ ($\mathbf{1}^{4+}$: cyclo[2](2,6-di(1H-imidazol-1-yl)pyridine)[2](1,4-dimethylenebenzene)) in a mixture containing 1 mL aqueous ammonia (between 25-28% by mass), 3.5 mL water and 4.5 mL acetonitrile. This solution was stirred hermetically at 313K for 24h. The reaction was then cooled to room temperature and acetonitrile was removed *via* blowing with compressed air, which gave rise to a light yellow precipitate (the crude product). The product was then collected via filtration and washing with 2 ml of water. Herein, reference to %$NH_3\cdot H_2O$ is meant to represent the volume fraction of $NH_3\cdot H_2O$ (25-28% by mass) used in the final solution. The reaction solvent consists of acetonitrile (4.5 mL) and dilutes aqueous ammonia (4.5 mL). The latter was obtained by mixing concentrated aqueous ammonia (25-28% by weight) with water to give the final stated concentration of ammonia. The ratio of the initial organic and aqueous phases was 1:1 in all experiments. It is suggested that the crude product mainly contains $\mathbf{1}^{4+}\cdot 4PF_6^-$, the single ring-opened product $\mathbf{I}^{3+}\cdot 3PF_6^-$, the two ring opened product $\mathbf{II}^{2+}\cdot 2\ PF_6^-$ (*i.e.*, $\mathbf{2}^{2+}\cdot 2PF_6^-$ and $\mathbf{3}^{2+}\cdot 2PF_6^-$) (cf. Figure S2 and S3).

^{1}H NMR and ESI mass spectrometric analysis of the synthesis of [2^{2+}·2PF_6^-] and [3^{2+}·2PF_6^-] under various reaction conditions

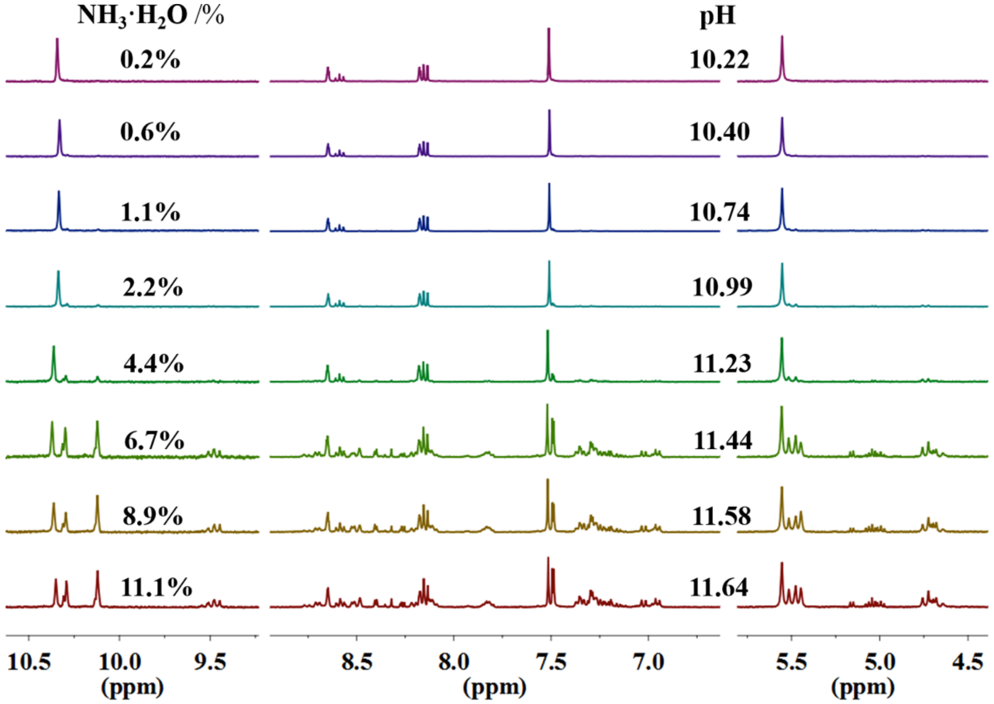

Figure S1. ^{1}H NMR spectra recorded in DMSO-d_6 (400 MHz, 300K) representing the crude products obtained from the ring-opening reaction of **1**$^{4+}$·4PF_6^- using different concentrations of NH_3·H_2O at 288K for 45h. Here NH_3·H_2O% represents the volume fraction of NH_3·H_2O (25-28% by weight) in the final solution. The pH value shown is that of the initial solvent system.

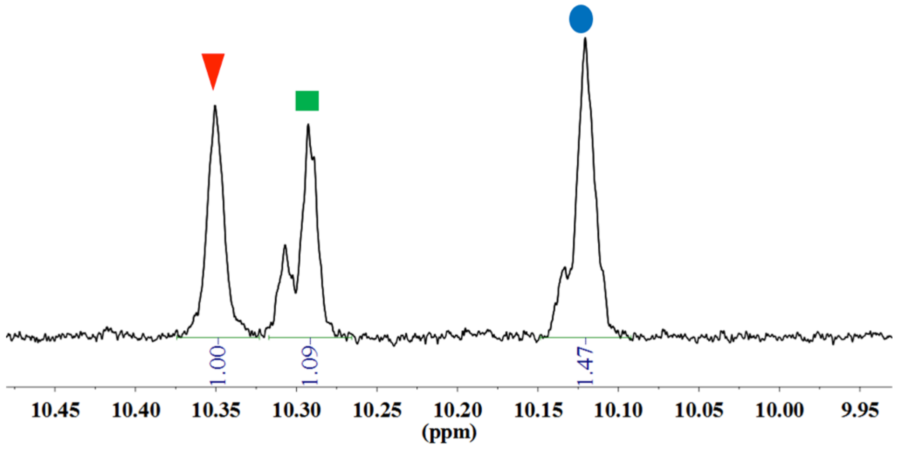

Figure S2. The expanded ^{1}H NMR spectrum recorded in DMSO-d_6 (400 MHz, 300K) of the crude product obtained from the reaction wherein the initial solvent pH was 11.64 and the reaction was run at 288K for 45 h. The proton signals of the C-H protons between the two nitrogen atoms on the imidazolium rings of **1**$^{4+}$ (labeled as "▼"), **I**$^{3+}$ (labeled as "■") and **II**$^{2+}$ (labeled as "●"). The relative amounts of each independent component in the crude product were obtained from the integrated areas.

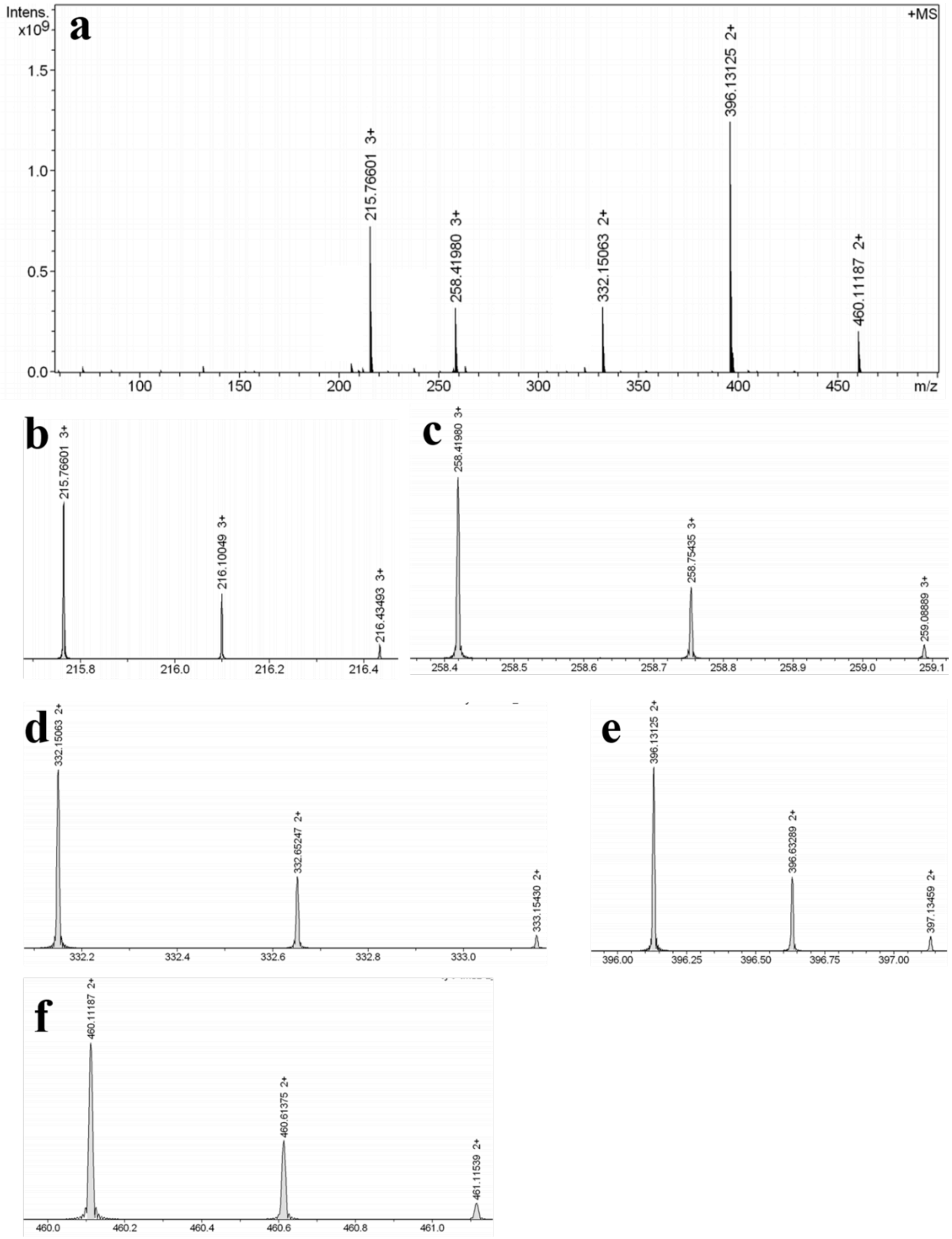

Figure S3. The full view (a) and expanded view (b) of the ESI high resolution mass spectrum of the crude product obtained from the ring-opening reaction of $\mathbf{1}^{4+}\cdot 4PF_6^-$ which was conducted at 288K for 45h at an initial pH of 11.64. It is implied that the crude product contained at least three components $\mathbf{1}^{4+}$, $\mathbf{I}^{3+}$ and $\mathbf{II}^{2+}$: calcd. for $C_{38}H_{34}N_{10}PF_6$ ($[\mathbf{1}^{4+}\cdot PF_6^-]^{3+}$) 258.4203. Found: 258.4198; calcd. for ($[\mathbf{1}^{4+}\cdot 2PF_6^-]^{2+}$) 460.1126. Found: 460.1119; calcd. for $C_{38}H_{35}N_{10}O$ ($[\mathbf{I}^{3+}]$) 215.7665. Found: 215.7660; calcd. for $C_{38}H_{36}N_{10}O_2$ ($[\mathbf{II}^{2+}]$) 332.1512. Found: 332.1506; calcd. for $C_{36}H_{36}N_{10}PF_6K$ ($[\mathbf{II}^{2+}\text{-}2CHO+K+2H+PF_6^-]^{2+\bullet}$) 396.1202. Found: 396.1313.

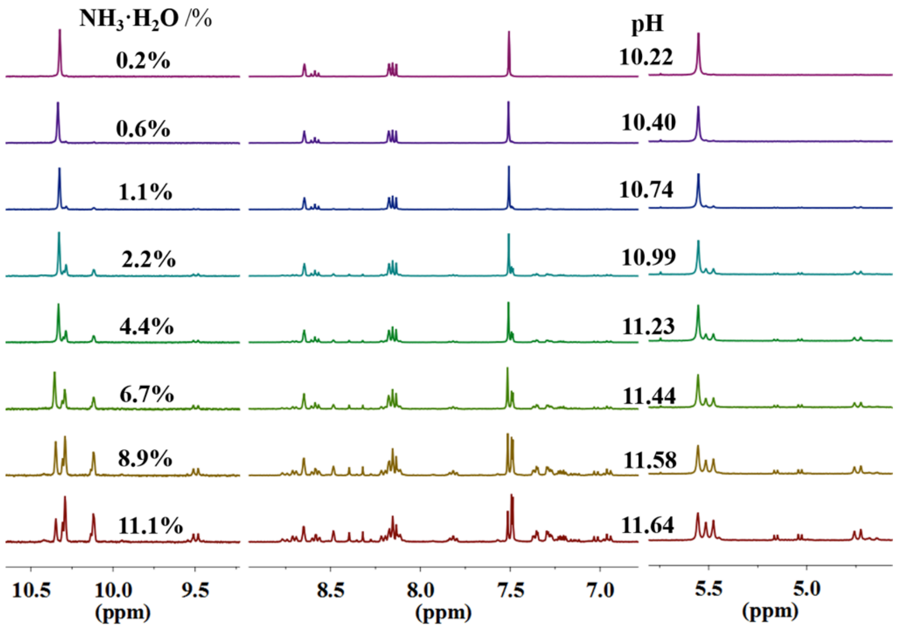

Figure S4. ^{1}H NMR spectra recorded in DMSO-d_6 (400 MHz, 300K) representing the crude products obtained from the ring-opening reaction of $\mathbf{1}^{4+}\cdot 4PF_6^-$ using different concentrations of $NH_3\cdot H_2O$ at 288K for 96 h. Here, $NH_3\cdot H_2O$% represents the volume fraction of $NH_3\cdot H_2O$ (25-28% by weight) in the final solution. The pH value shown is that of the initial solvent system.

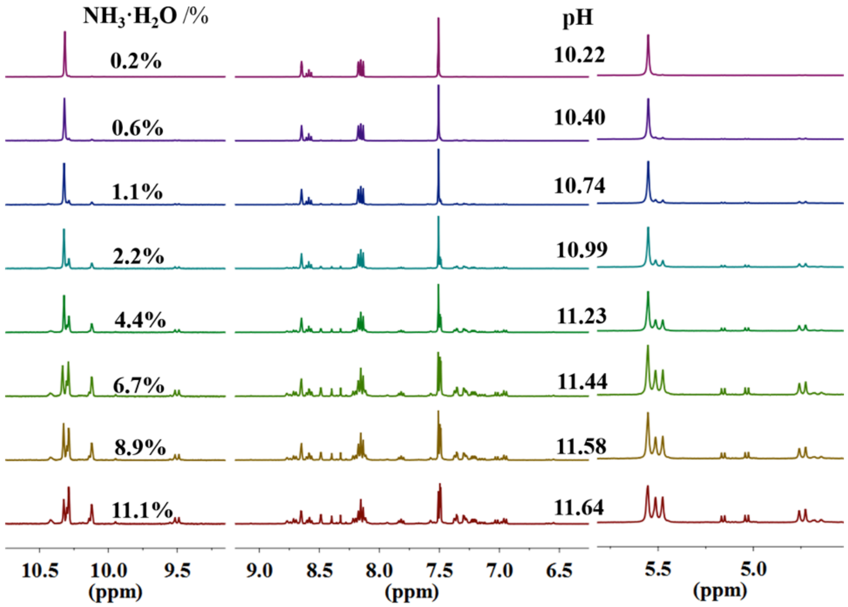

Figure S5. ^{1}H NMR spectra recorded in DMSO-d_6 (400 MHz, 300K) representing the crude products obtained from the ring-opening reaction of $\mathbf{1}^{4+}\cdot 4PF_6^-$ using different concentrations of $NH_3\cdot H_2O$ at 288K for 120h. Here $NH_3\cdot H_2O$% represents the volume fraction of $NH_3\cdot H_2O$ (25-28% by weight) in final solution. The pH value shown is that of the initial solvent system.

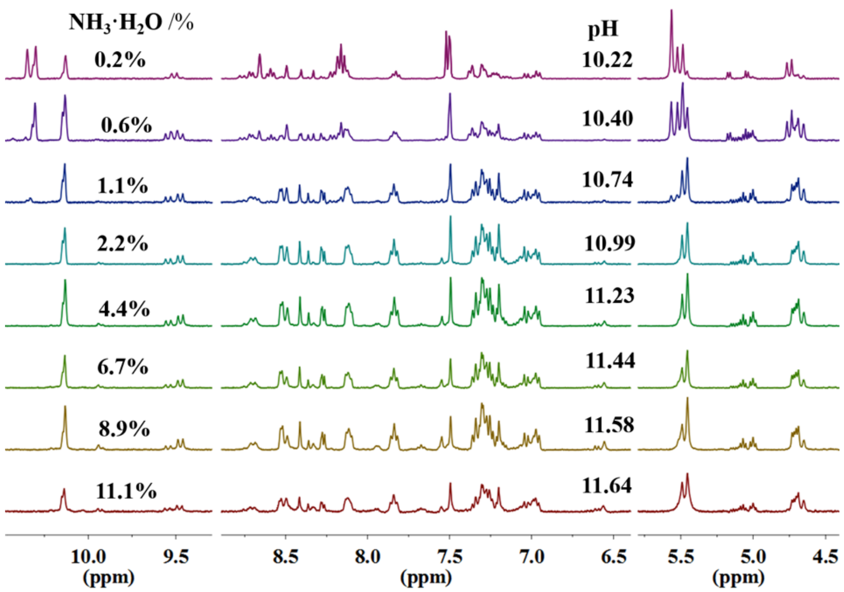

Figure S6. ^{1}H NMR spectra recorded in DMSO-d_6 (400 MHz, 300K) representing the crude products obtained from the ring-opening reaction of $\mathbf{1}^{4+}\cdot 4PF_6^-$ using different concentrations of $NH_3\cdot H_2O$ at 298K for 45 h. Here $NH_3\cdot H_2O$% represents the volume fraction of $NH_3\cdot H_2O$ (25-28% by weight) in the final solution. The pH value shown is that of the initial solvent system.

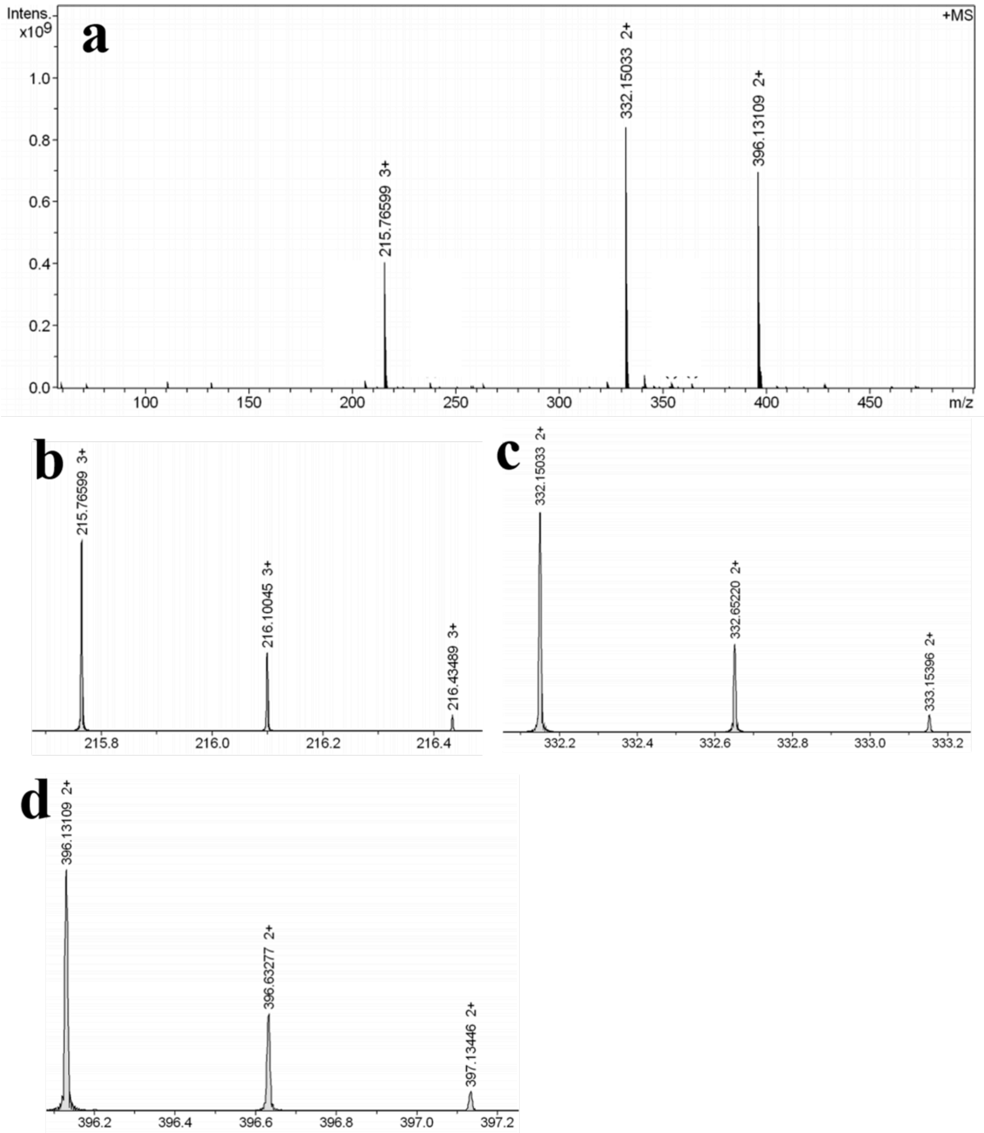

Figure S7. Full view (a) and expanded view (b) of the ESI high resolution mass spectrum of the crude product obtained from the ring-opening reaction of $\mathbf{1}^{4+}$·4PF_6^-, which was conducted at 298K for 45 h at an initial pH of 10.40. It was found that signals for $\mathbf{1}^{4+}$ disappeared in the crude product, and only the signals related to component $\mathbf{I}^{3+}$ and $\mathbf{II}^{2+}$ could be detected as follows: calcd. for $C_{38}H_{35}N_{10}O$ ([$\mathbf{I}^{3+}$]) 215.7665. Found: 215.7660; calcd. for $C_{38}H_{36}N_{10}O_2$ ([$\mathbf{II}^{2+}$]) 332.1512. Found: 332.1503; calcd. for $C_{36}H_{36}N_{10}PF_6K$ ([$\mathbf{II}^{2+}$-2CHO+K+2H+PF_6^-]$^{2+\bullet}$) 396.1202. Found: 396.1311.

Table S1 below compiles and summarizes the above results.

Table S1. Optimization of the ring-opening reaction conditions for **1**$^{4+}$.

Entry	$NH_3·H_2O$ [a]/%	pH$_a$ [b]	Total percent conversion/% (288 K, 45h)	Total percent conversion/% (288 K,96h)	Total percent conversion/%(288 K, 120h)	Total percent conversion/% (298 K, 45h)
1	0.2	10.22	$\leq$ 4.0	$\leq$ 4.0	$\leq$ 4.0	79
2	0.6	10.40	11	9	11	98
3	1.1	10.74	12	20	26	99
4	2.2	10.99	19	46	46	100
5	4.4	11.23	33	49	60	100
6	6.7	11.44	78	62	77	100
7	8.9	11.58	78	78	75	100
8	11.1	11.64	81	87	85	100

[a] Volume fraction of $NH_3·H_2O$ used in the final solution. The mixture of reaction solvents consisted of acetonitrile (4.5 mL) and dilute aqueous ammonia (4.5 mL). The latter was obtained by mixing concentrated aqueous ammonia (25-28% by weight) with water to give the final stated concentration of ammonia. The ratio of the initial organic and aqueous phases was 1:1 in all experiments. [b] pH of the initial solvent system.

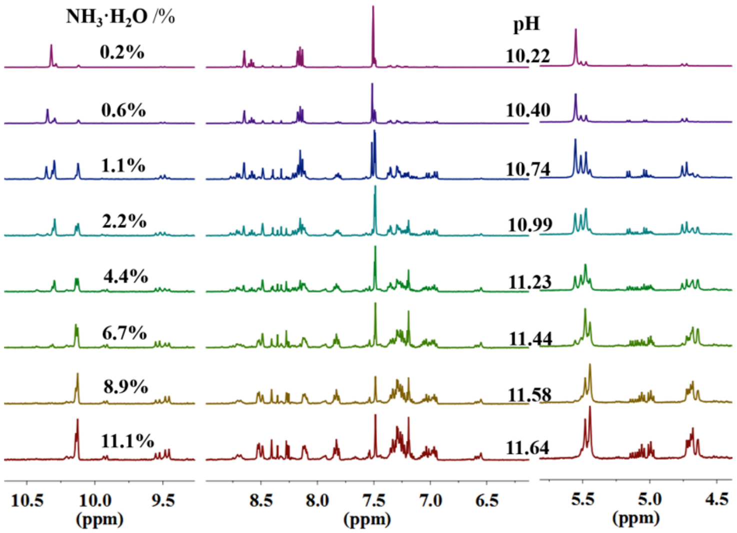

Figure S8. ^{1}H NMR spectra recorded in DMSO-d_6 (400 MHz, 300K) representing the crude products obtained from the ring-opening reaction of **1**$^{4+}$·$4PF_6^-$ using different concentrations of $NH_3·H_2O$ at 333K for 2 h. Here $NH_3·H_2O$% represents the volume fraction of $NH_3·H_2O$ (25-28% by weight) in final solution. The pH value shown is that of the initial solvent system.

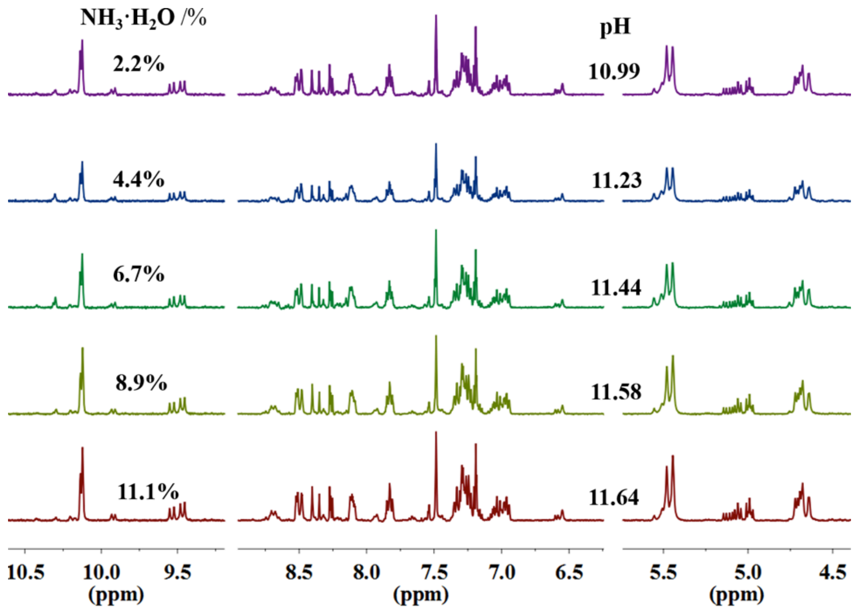

Figure S9. ^{1}H NMR spectra recorded in DMSO-d_6 (400 MHz, 300K) representing the crude products obtained from the ring-opening reaction of **1**$^{4+}$·4PF_6^- using different concentrations of NH_3·H_2O at 313K for 24h. Here NH_3·H_2O% represents the volume fraction of NH_3·H_2O (25-28% by weight) in the final solution. The pH value shown is that of the initial solvent system.

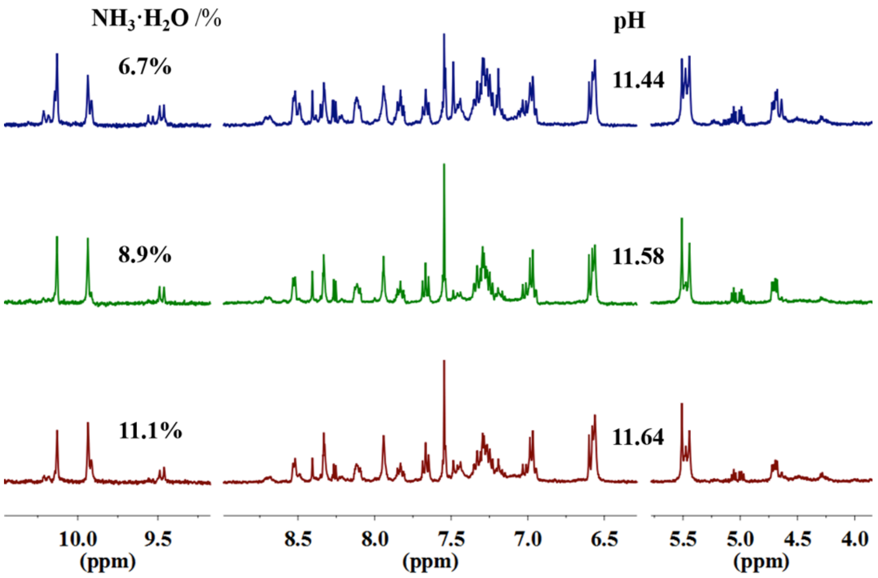

Figure S10. ^{1}H NMR spectra recorded in DMSO-d_6 (400 MHz, 300K) representing the crude products obtained from the ring-opening reaction of **1**$^{4+}$·4PF_6^- using different concentrations of NH_3·H_2O at 313K for 48h. Here NH_3·H_2O% represents the volume fraction of NH_3·H_2O (25-28% by weight) in the final solution. The pH value shown is that of the initial solvent system.

Simulated ratio of 2^{2+}·$2PF_6^-$ and 3^{2+}·$2PF_6^-$ in the mixture

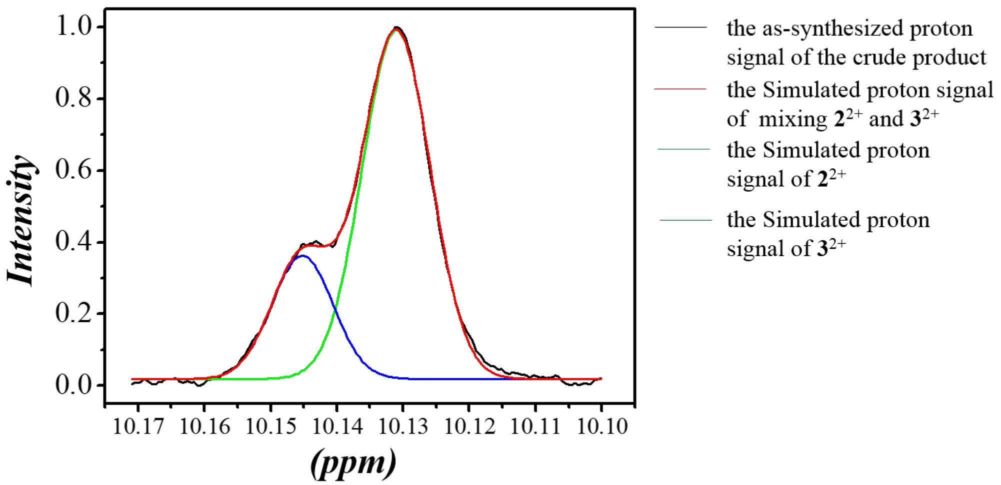

Figure S11. Expanded view of signals for H(1) (the proton signals with most lowest chemical shift on **2**$^{2+}$·2PF_6^- and **3**$^{2+}$·2PF_6^-) in the crude product (i.e., the mixture of **2**$^{2+}$ and **3**$^{2+}$ obtained via the general reaction conditions) in DMSO-d_6 at 300K (400 MHz). The black curve is the as-synthesized peak data for H(1) in **2**$^{2+}$ and **3**$^{2+}$. The red curve is the simulated peak data for H(1) in the mixture of **2**$^{2+}$ and **3**$^{2+}$. The green curves is the simulated peak data for the H(1) signals in **2**$^{2+}$. The blue curves is the simulated peak data for the H(1) signals of **3**$^{2+}$. The mathematical area ratio, consistent with the molar ratio of **2**$^{2+}$·2PF_6^- and **3**$^{2+}$·2PF_6^- in the crude product, is approximately 3:1.

Synthesis of *trans*-cyclo[2]((Z)-*N*-(2-((6-(1*H*-imidazol-1-yl)pyridin-2-yl)amino)vinyl)formamide)[2](1,4-bismethylbenzene)hexafluorophosphate [2^{2+}·$2PF_6^-$]:

Using the aforementioned procedure, a 25 ml small vial was charged with 50.0 mg (0.041 mmol) of **1**$^{4+}$·4PF_6^- in aqueous ammonia (between 25-28% by mass), water, and acetonitrile (v/v/v, 1/3.5/4.5, 9 mL). The pH of the initial solvent system was 11.64. The solution was stirred in the absence of air at 313 K for 24 h. The reaction mixture was then cooled to room temperature and the solvent was subsequently removed by blowing the sample with compressed air. Subsequently, a light yellow precipitate (86%-93% crude yield of **2**$^{2+}$·2PF_6^- plus **3**$^{2+}$·2PF_6^-) was collected and then washed with 2 mL water. This crude product was recrystallized from a

mixture of acetonitrile and dioxane (2:1, v/v), resulting in isolation of the pure product $\mathbf{2}^{2+}\cdot 2PF_6^-$ as a light yellow solid in 42% overall yield (37 mg); ^{1}H NMR (600MHz, DMSO-d_6, 300K) δ 10.13 (br, 2H, imidazolium ring –N=C**H**-N-), 9.47 (d, *J* = 11.1 Hz, 1H, ring-opened imidazolium –CH=CH-N**H**-CH_2-), 8.67 (m, 1H, ring-opened imidazolium –CH=CH-N**H**-Py), 8.51（d，2H, imidazolium ring –C**H**=CH-N-CH_2-）8.42 (s, 1H, -CH=CH-N(C**H**O)-CH_2- on ring-opened imidazolium group), 8.28（d, *J* = 8.5 Hz, 1H, -CH=CH-N(C**H**O)-Py on ring-opened imidazolium group）,8.11 (t, 2H, imidazolium ring –CH=C**H**-N-CH_2-), 7.84 (m, 2H, H_{γ}-Py), 7.33 (m, 8H, ArH), 7.25 (m, 2H, H_{β}-Py-N(imidazolium ring)), 7.19 (m, 1H, -C**H**=CH-N(CHO)-Py on ring-opened imidazolium group), 7.04 (d, *J* = 8.2 Hz, 1H, $H_{\beta 1}$-Py-N-CHO), 6.99 (m, 1H, -C**H**=CH-N(CHO)-CH_2- on ring-opened imidazolium group), 6.98 (m, 1H, $H_{\beta 2}$-Py-NH), 5.46 (s, 4H, -C-C**H**$_2$-N(imidazolium ring)), 5.08 (m, 1H, -CH=C**H**-N(CHO)-Py on ring-opened imidazolium group), 5.02 (m, 1H, -CH=C**H**-N(CHO)-CH_2- on ring-opened imidazolium group), 4.72 (m, 4H, -C-C**H**$_2$-N(ring-opened imidazolium group)); ^{13}C NMR (150 MHz, DMSO-d_6, 300K) δ 163.3, 162.5, 153.3, 152.9, 144.5, 144.4, 141.0, 137.7, 137.3, 134.8, 134.2, 134.1, 134.0, 133.9, 128.4, 128.0, 127.7, 127.6, 127.5, 127.4, 124.0, 123.9, 123.8, 123.7, 121.5, 121.2, 119.4, 118.8, 118.6, 111.1, 110.9, 104.9, 104.1, 102.9, 102.9, 66.3, 52.1, 51.1, 45.5, 45.4; FT-ICRMS: calcd. for $C_{38}H_{36}N_{10}O_2PF_6$ ([$\mathbf{2}^{2+}\cdot PF_6^-$]$^+$) 809.2665. Found: 809.2657; calcd. for $C_{38}H_{35}N_{10}O_2$ ([($\mathbf{2}^{2+}$-H)]$^{+\bullet}$) 663.2944. Found: 663.2934.

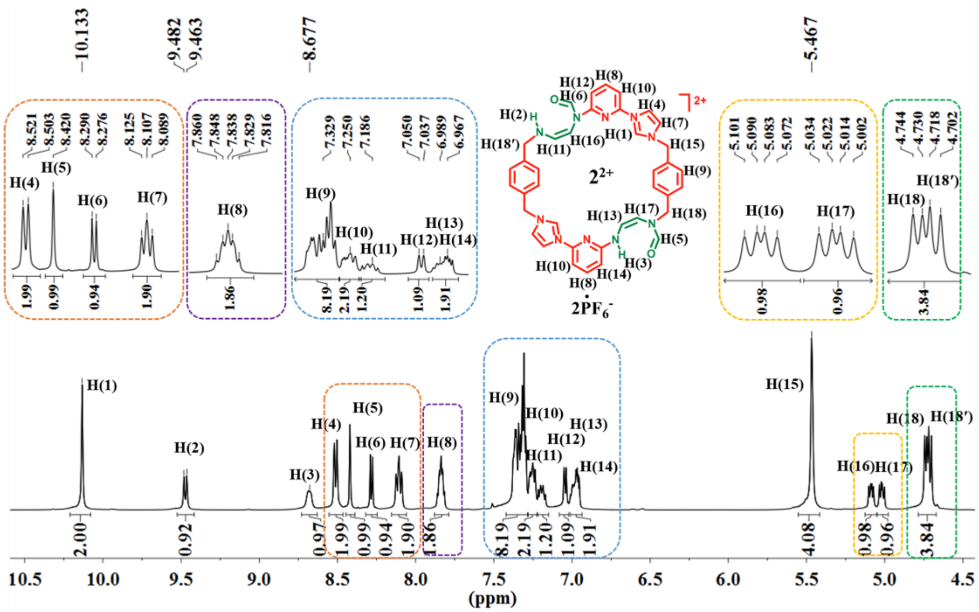

Figure S12. ^{1}H NMR spectrum of $\mathbf{2}^{2+}\cdot 2PF_6^-$ (6.0×10^{-2} M) collected in DMSO-d_6 at 300K (600 MHz).

a

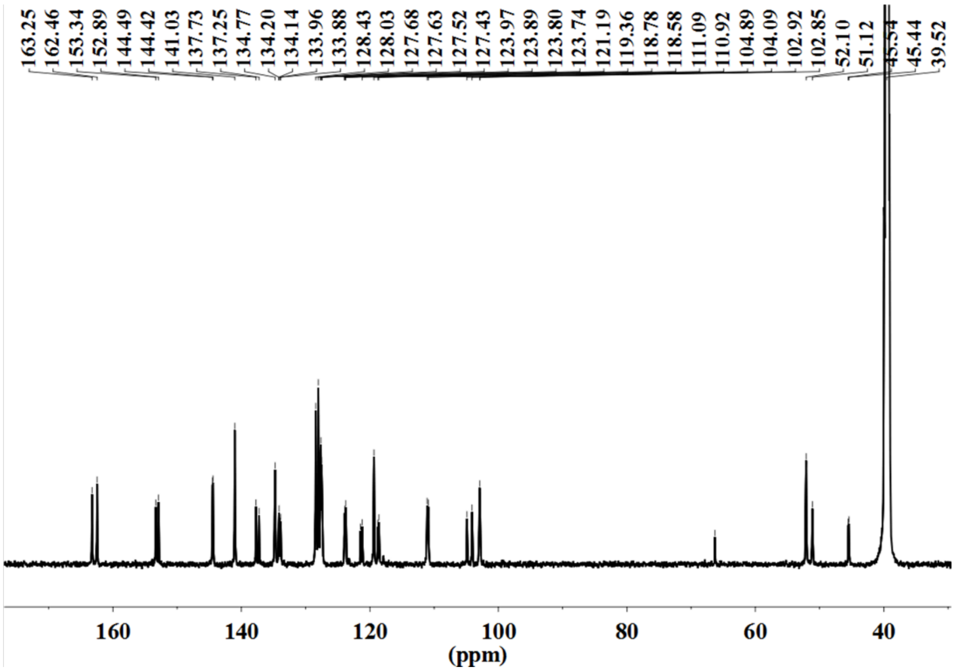

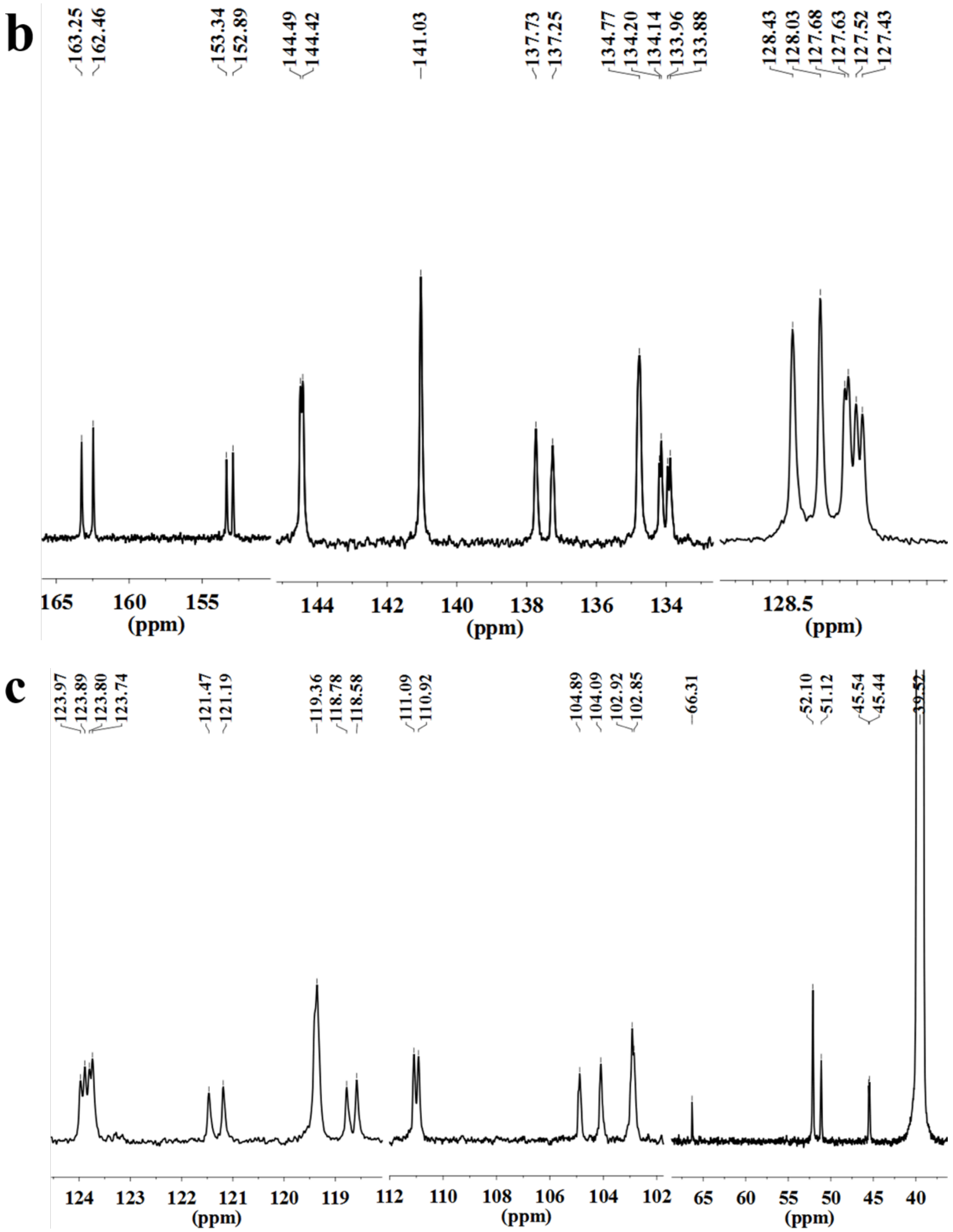

Figure S13. Full view (a) and expanded views (b), (c) of the ^{13}C NMR spectrum of $\mathbf{2}^{2+}\cdot 2PF_6^-$ (6.0×10^{-2} M) collected in DMSO-d_6 at 300K (150 MHz).

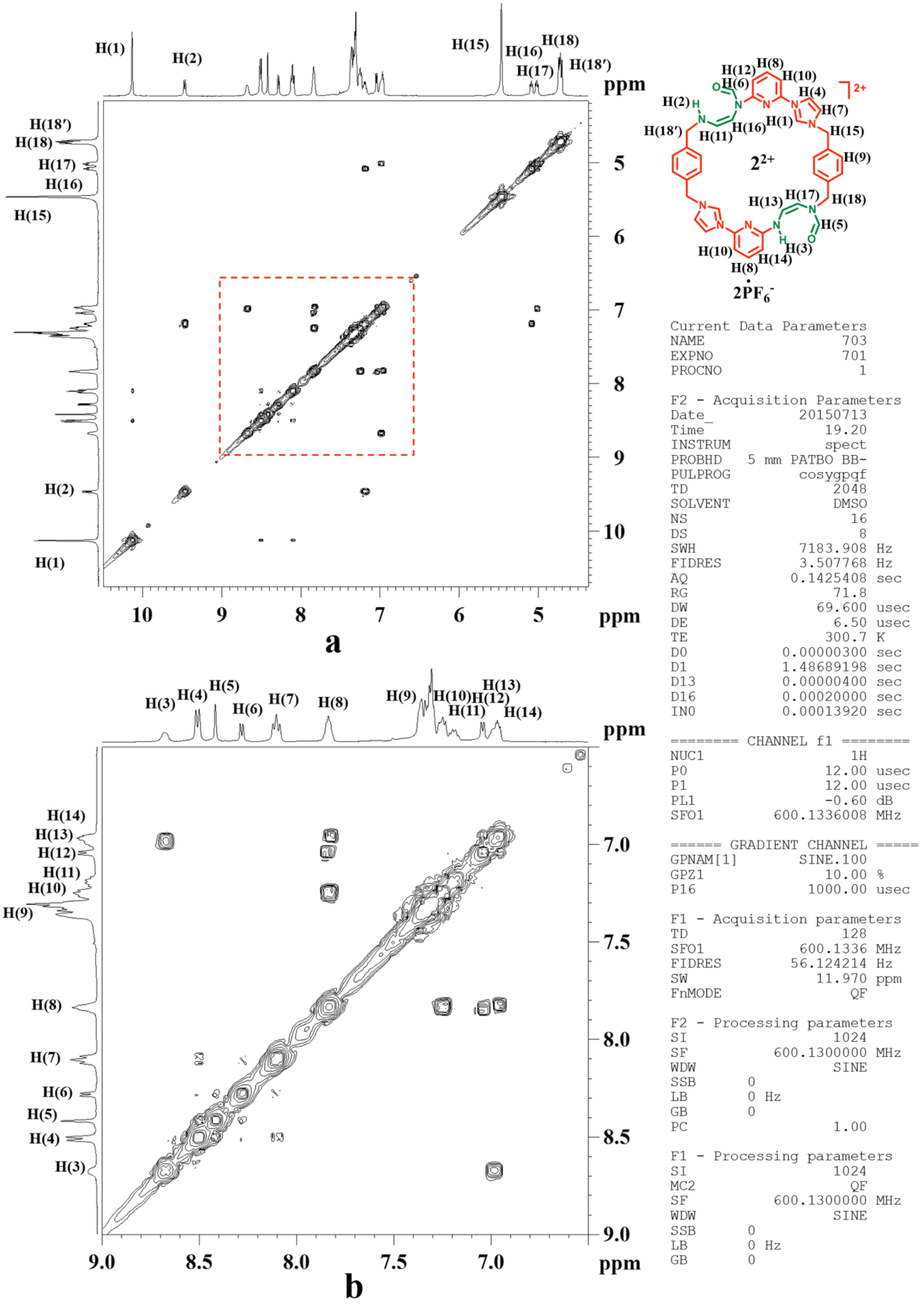

Figure S14. Full view (a) and expanded view (b) of the 2D COSY NMR spectrum of $\mathbf{2}^{2+}\cdot 2PF_6^-$ (6.0×10^{-2} M) collected in DMSO-d_6 at 300K (600 MHz).

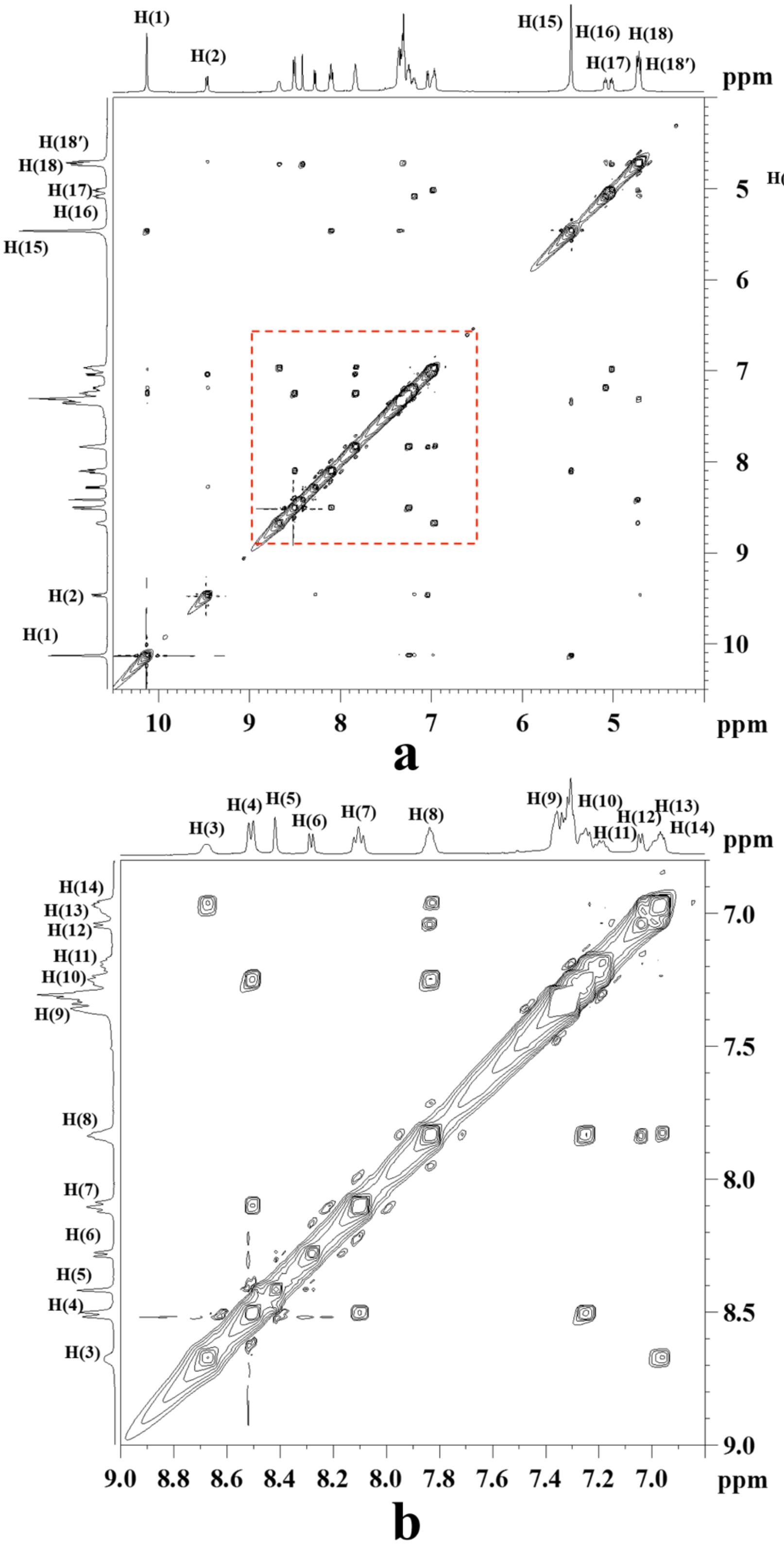

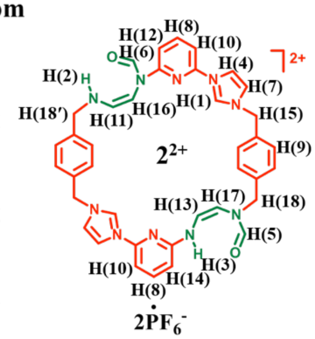


```
Current Data Parameters
NAME                  703
EXPNO                 702
PROCNO                  1

F2 - Acquisition Parameters
Date_            20150713
Time                16.46
INSTRUM             spect
PROBHD   5 mm PATBO BB-
PULPROG        noesygpph
TD                   2048
SOLVENT              DMSO
NS                     16
DS                     16
SWH              7183.908 Hz
FIDRES           3.507768 Hz
AQ              0.1425408 sec
RG                   71.8
DW                 69.600 usec
DE                   6.50 usec
TE                  301.3 K
D0             0.00005432 sec
D1             1.50000000 sec
D8             0.60000002 sec
D16            0.00020000 sec
IN0            0.00013920 sec

======== CHANNEL f1 ========
NUC1                   1H
P1                  12.00 usec
P2                  24.00 usec
PL1                 -0.60 dB
SFO1         600.1336008 MHz

====== GRADIENT CHANNEL =====
GPNAM[1]         SINE.100
GPZ1                40.00 %
P16               1000.00 usec

F1 - Acquisition parameters
TD                    256
SFO1             600.1336 MHz
FIDRES          28.062107 Hz
SW                 11.970 ppm
FnMODE         States-TPPI

F2 - Processing parameters
SI                   1024
SF           600.1300000 MHz
WDW                 QSINE
SSB                     2
LB          0 Hz
GB          0
PC                   1.40

F1 - Processing parameters
SI                   1024
MC2            States-TPPI
SF           600.1300000 MHz
WDW                 QSINE
SSB                     2
LB          0 Hz
GB          0
```

Figure S15. Full view (a) and expanded view (b) of the 2D NOESY NMR spectrum of $\mathbf{2}^{2+}\cdot 2PF_6^-$ (6.0×10^{-2} M) collected in DMSO-d_6 at 300K (600 MHz).

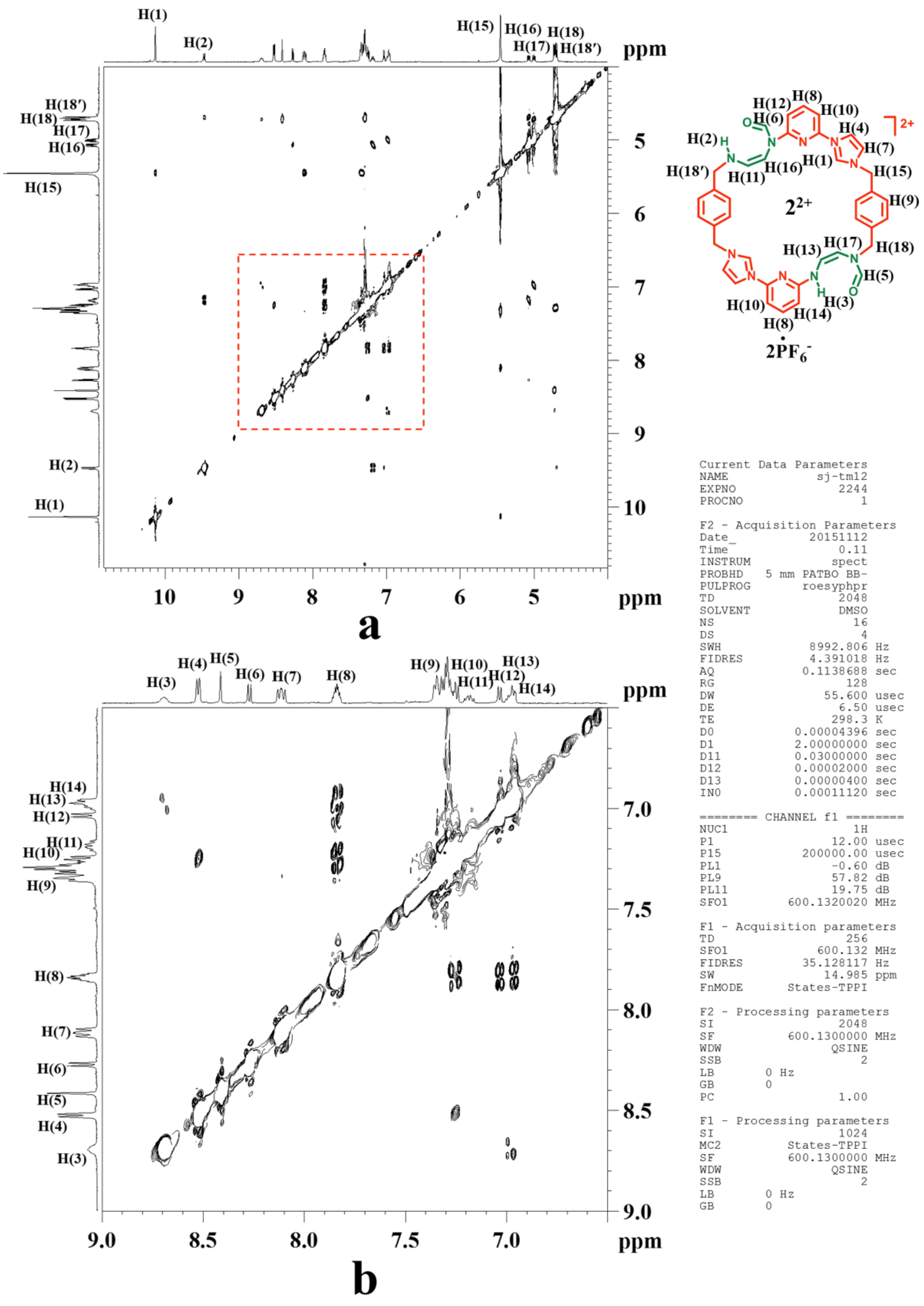

Figure S16. Full view (a) and expanded view (b) of the 2D ROESY NMR spectrum of $\mathbf{2}^{2+}\cdot 2PF_6^-$ (6.0×10^{-2} M) collected in DMSO-d_6 at 300K (600 MHz).

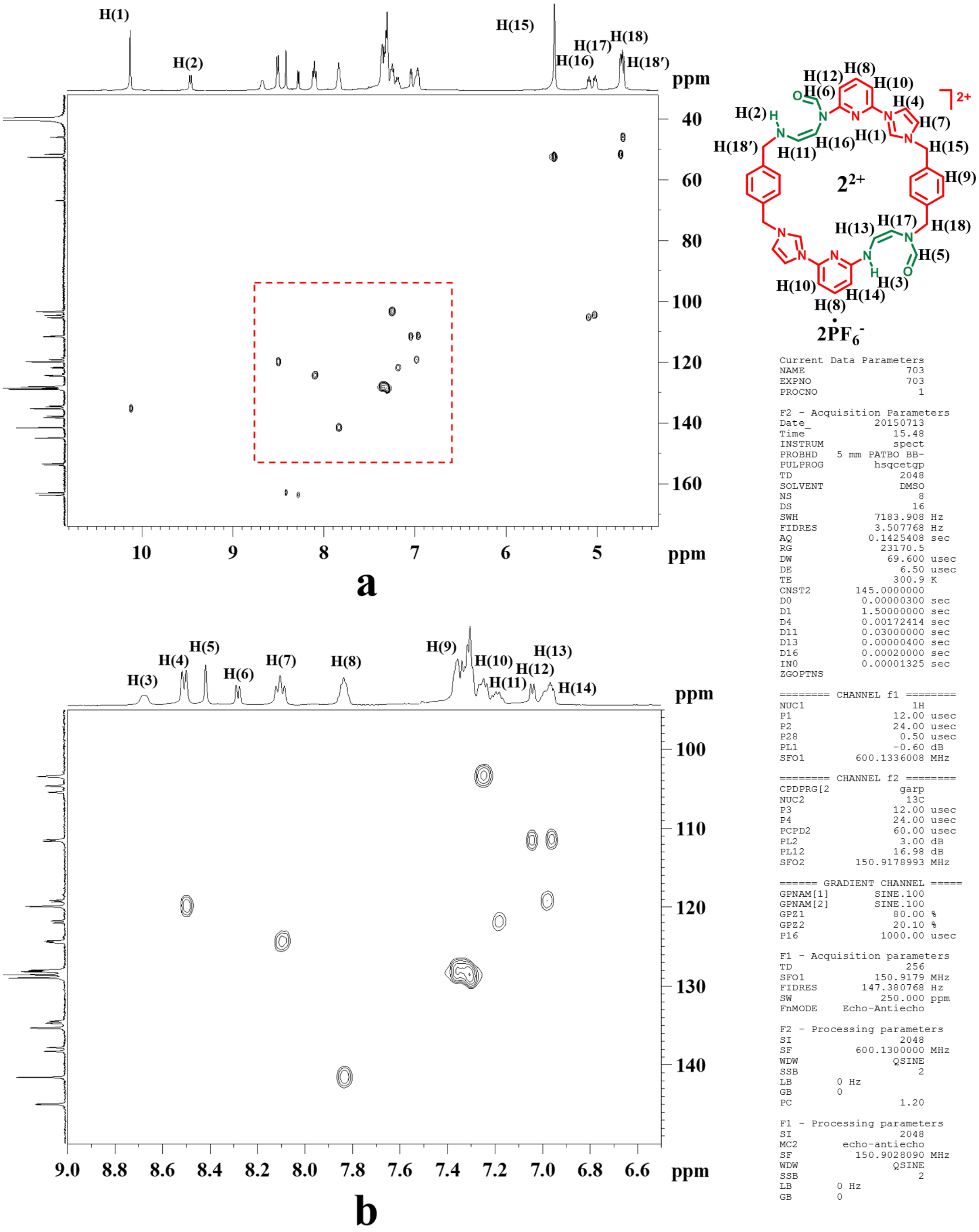

Figure S17. Full view (a) and expanded view (b) of the HSQC NMR spectrum of $\mathbf{2}^{2+}\cdot 2PF_6^-$ (6.0 × 10^{-2} M) collected in DMSO-d_6 at 300K (600 MHz).

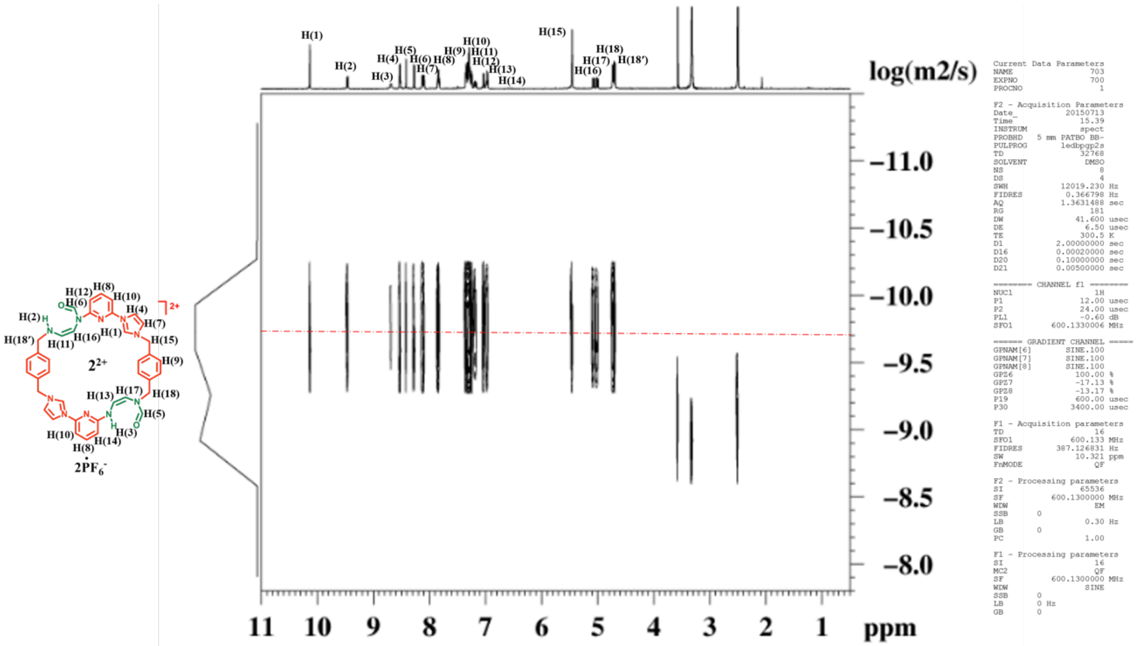

Figure S18. 2D DOSY NMR spectrum of $\mathbf{2}^{2+}\cdot 2PF_6^-$ (6.0×10^{-2} M) collected in DMSO-d_6 at 300K (600 MHz).

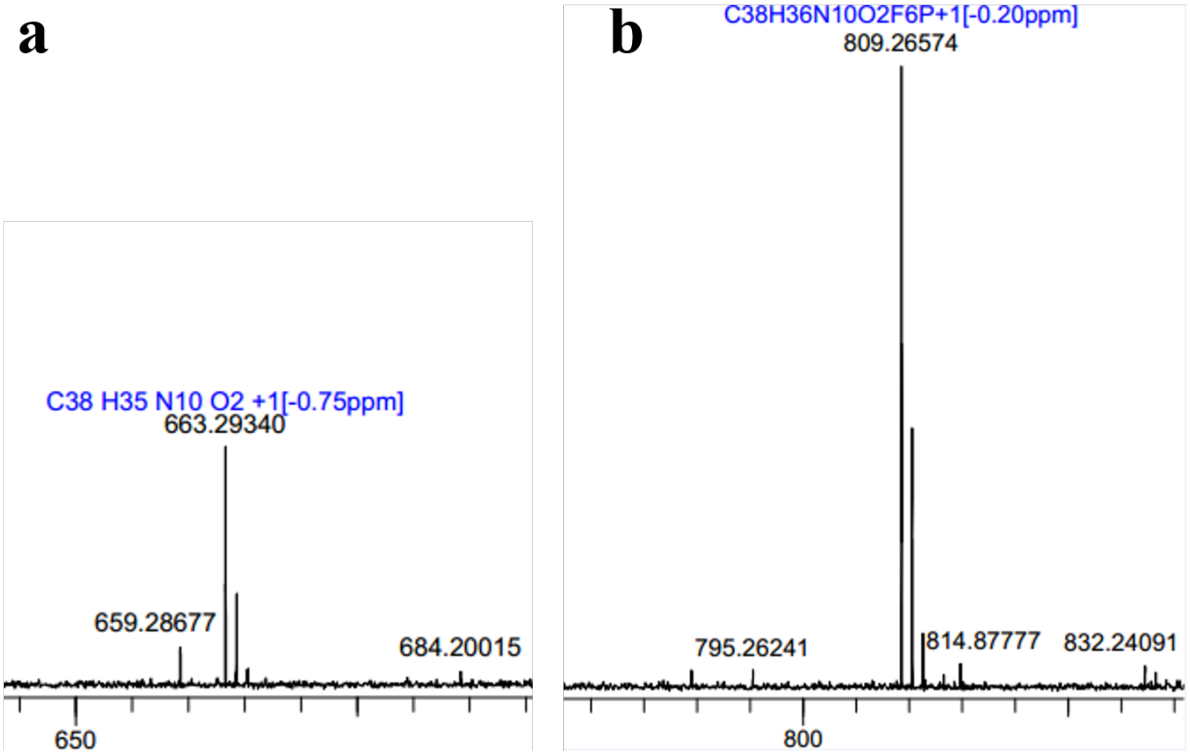

Figure S19. Expanded views (i.e., (a) and (b)) of the ESI high-resolution mass spectrum of $\mathbf{2}^{2+}\cdot 2PF_6^-$.

Structural analysis of $2^{2+}\cdot 2PF_6^-$ in DMSO-d_6 at 300K

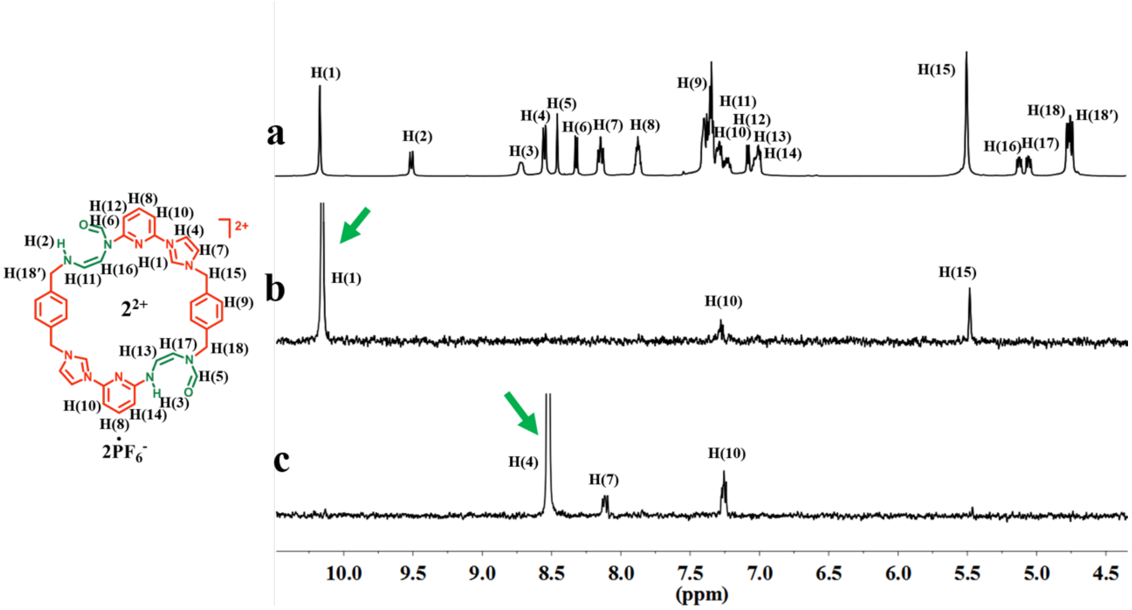

Figure S20. ^{1}H NMR (a), as well as the 1D-NOE NMR ((b) and (c)) spectra of $\mathbf{2}^{2+}\cdot 2PF_6^-$ as recorded in DMSO-d_6 at 300 K (600 MHz). The green arrow in (b) and (c) indicates irradiation at the frequency of H(1) and H(4) on $\mathbf{2}^{2+}$. It is suggested that macrocycle $\mathbf{2}^{2+}$ has a flexible structure as inferred from the observed correlations between H(1), H(4) and H(10).

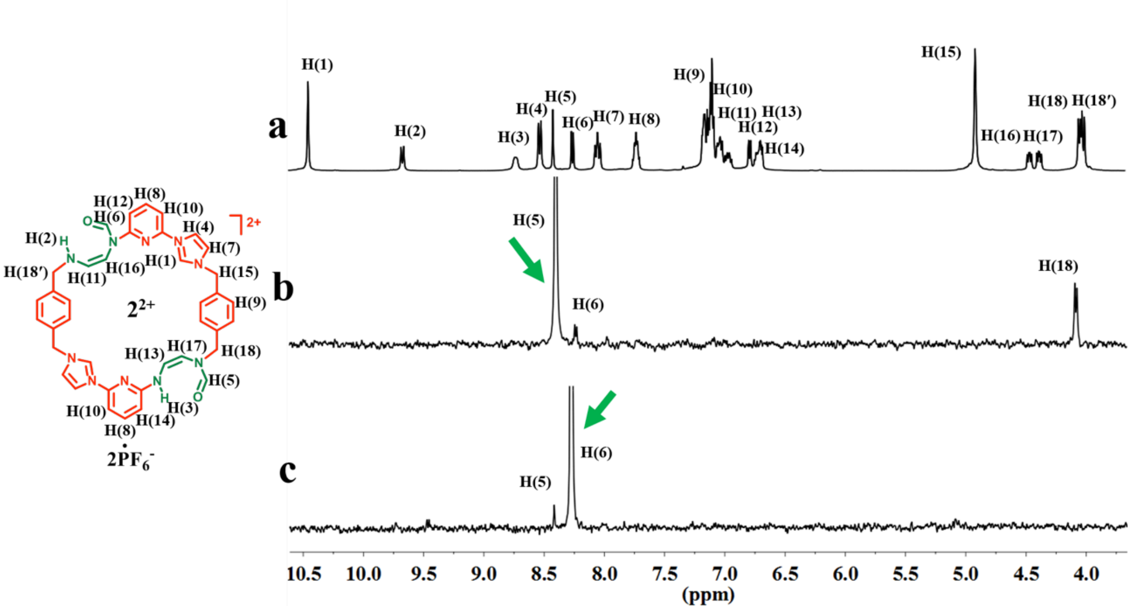

Figure S21. ^{1}H NMR (a), as well as the 1D-NOE NMR ((b) and (c)) spectra of $\mathbf{2}^{2+}\cdot 2PF_6^-$ in DMSO-d_6 at 300 K (600 MHz). The green arrow in (b) and (c) indicates irradiation at the frequency of H(5) and H(6) on $\mathbf{2}^{2+}$.

Structural analysis of $\mathbf{2}^{2+}\cdot 2PF_6^-$ in DMF-d_7

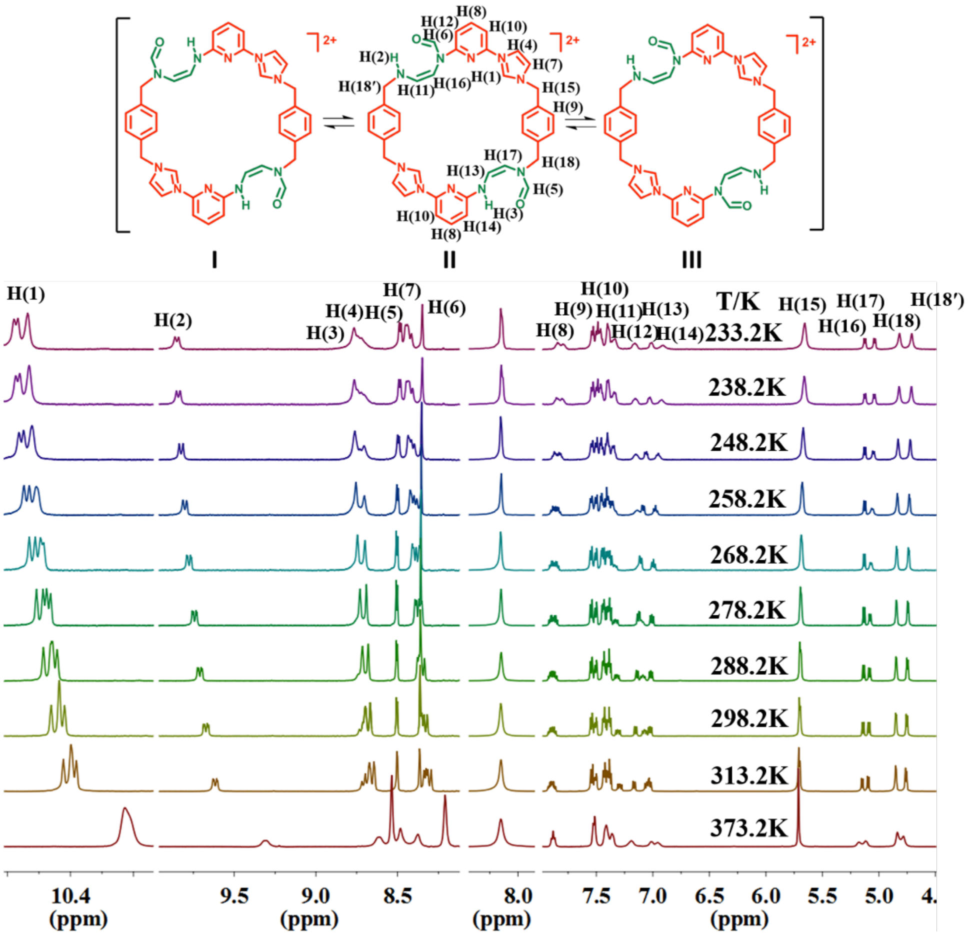

Figure S22. Three possible isomeric structural conversions of $\mathbf{2}^{2+}\cdot 2PF_6^-$, as well as the ^1H NMR spectra of $\mathbf{2}^{2+}\cdot 2PF_6^-$ in DMF-d_7 at different temperatures (500 MHz). At 278K, three isomers exists in DMF-d_7 solution in a ratio of about 1:2:1 (I:II:III).

a

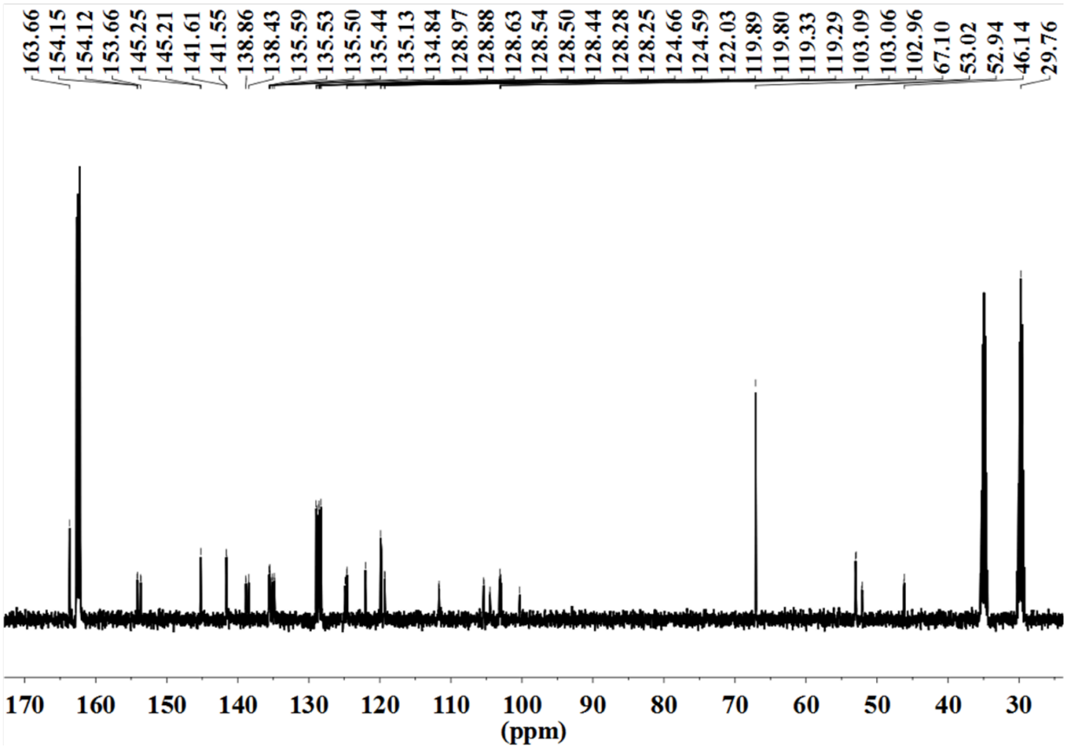
163.66
154.15
154.12
153.66
145.25
145.21
141.61
141.55
138.86
138.43
135.59
135.53
135.50
135.44
135.13
134.84
128.97
128.88
128.63
128.54
128.50
128.44
128.28
128.25
124.66
124.59
122.03
119.89
119.80
119.33
119.29
103.09
103.06
102.96
67.10
53.02
52.94
46.14
29.76
170 160 150 140 130 120 110 100 90 80 70 60 50 40 30
(ppm)

b

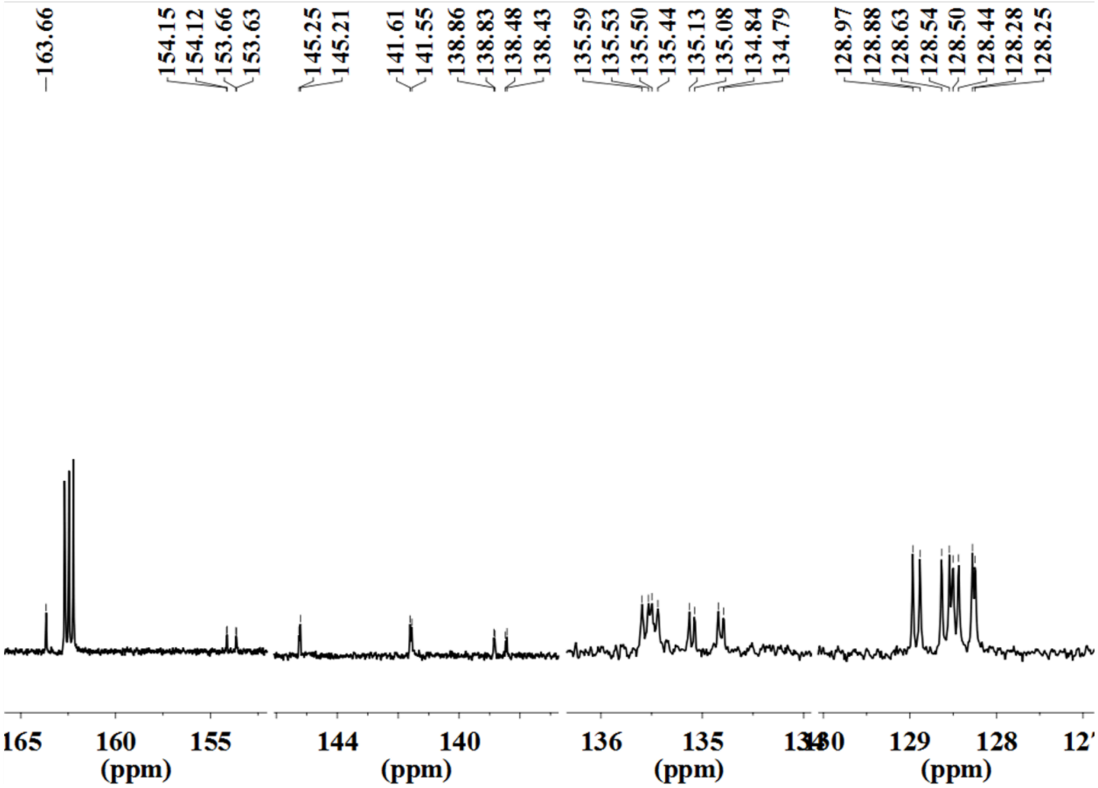
163.66
154.15
154.12
153.66
153.63
145.25
145.21
141.61
141.55
138.86
138.83
138.48
138.43
135.59
135.53
135.50
135.44
135.13
135.08
134.84
134.79
128.97
128.88
128.63
128.54
128.50
128.44
128.28
128.25
165 160 155
(ppm)
144 140
(ppm)
136 135
(ppm)
129 128
(ppm)

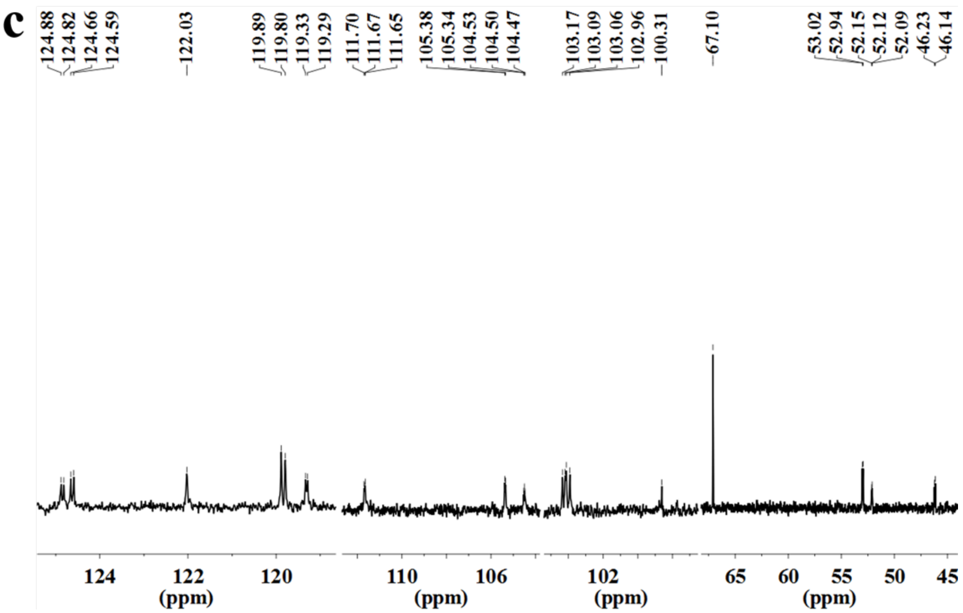

Figure S23. Full view (a) and expanded views ((b) and (c)) of the ^{13}C NMR spectrum of $\mathbf{2}^{2+}\cdot 2PF_6^-$ (2.0×10^{-2} M) collected in DMF-d_7 at 278K (125 MHz).

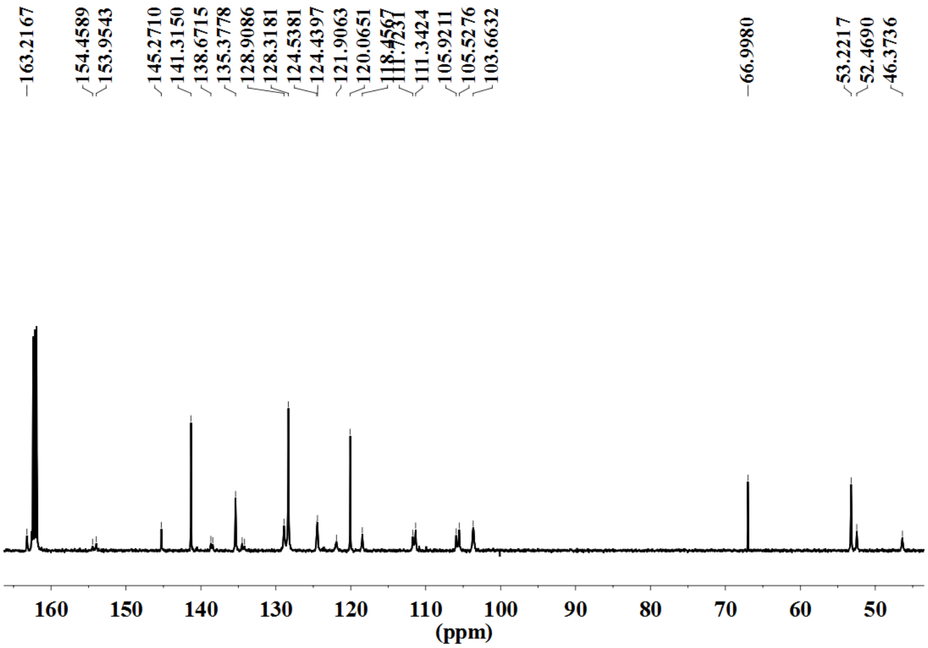

Figure S24. Full view of ^{13}C NMR spectrum of $\mathbf{2}^{2+}\cdot 2PF_6^-$ (2.0×10^{-2} M) collected in DMF-d_7 at 373K (125 MHz).

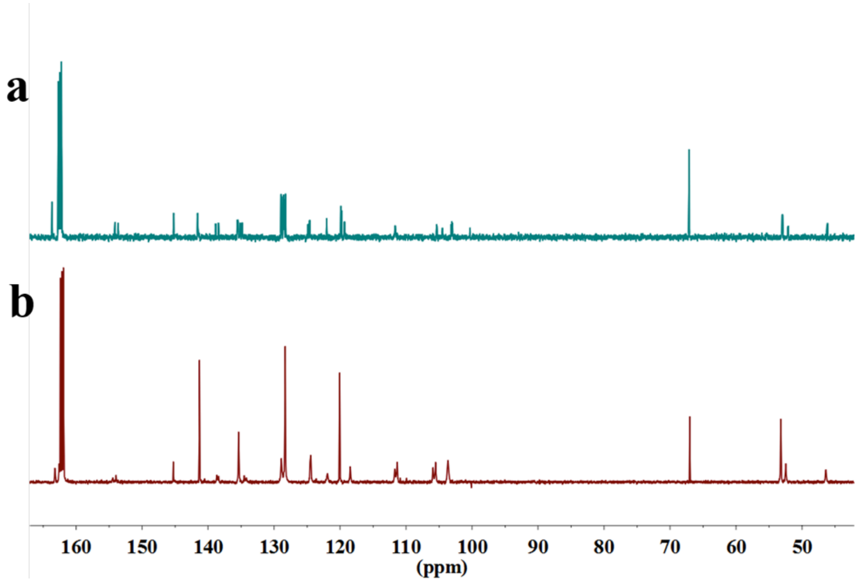

Figure S25. Comparison of the ^{13}C NMR spectra of $\mathbf{2}^{2+}\cdot 2PF_6^-$ (2.0×10^{-2} M) collected in DMF-d_7 at 278K (a) and 373K (b) (125 MHz).

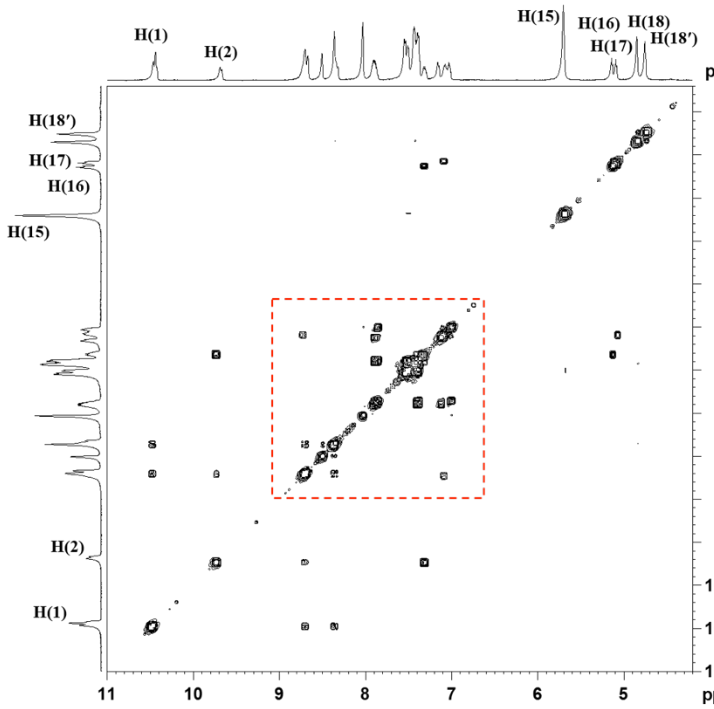

a

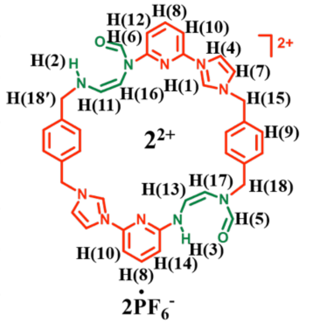


```
Current Data Parameters
NAME        SJ-TM12-DMF2
EXPNO                217
PROCNO                 1

F2 - Acquisition Parameters
Date_           20151013
Time               10.45
INSTRUM            spect
PROBHD   5 mm PABBO BB/
PULPROG       cosygpppqf
TD                  2048
SOLVENT              DMF
NS                     4
DS                    16
SWH             5656.108 Hz
FIDRES          2.761772 Hz
AQ             0.1810432 sec
RG                 64.12
DW                88.400 usec
DE                  6.50 usec
TE                 278.2 K
D0            0.00000300 sec
D1            2.00000000 sec
D11           0.03000000 sec
D12           0.00002000 sec
D13           0.00000400 sec
D16           0.00020000 sec
IN0           0.00017680 sec

======== CHANNEL f1 ========
SFO1         500.1331563 MHz
NUC1                  1H
P0                 10.00 usec
P1                 10.00 usec
P17              2500.00 usec
PLW1         10.00000000 W
PLW10         1.47930002 W

====== GRADIENT CHANNEL =====
GPNAM[1]        SMSQ10.100
GPZ1               10.00 %
P16              1000.00 usec

F1 - Acquisition parameters
TD                   128
SFO1            500.1332 MHz
FIDRES         44.188347 Hz
SW                11.309 ppm
FnMODE                QF

F2 - Processing parameters
SI                  1024
SF           500.1300000 MHz
WDW                QSINE
SSB       0
LB        0 Hz
GB        0
PC                  1.00

F1 - Processing parameters
SI                  1024
MC2                   QF
SF           500.1300000 MHz
WDW                QSINE
SSB       0
LB        0 Hz
GB        0
```

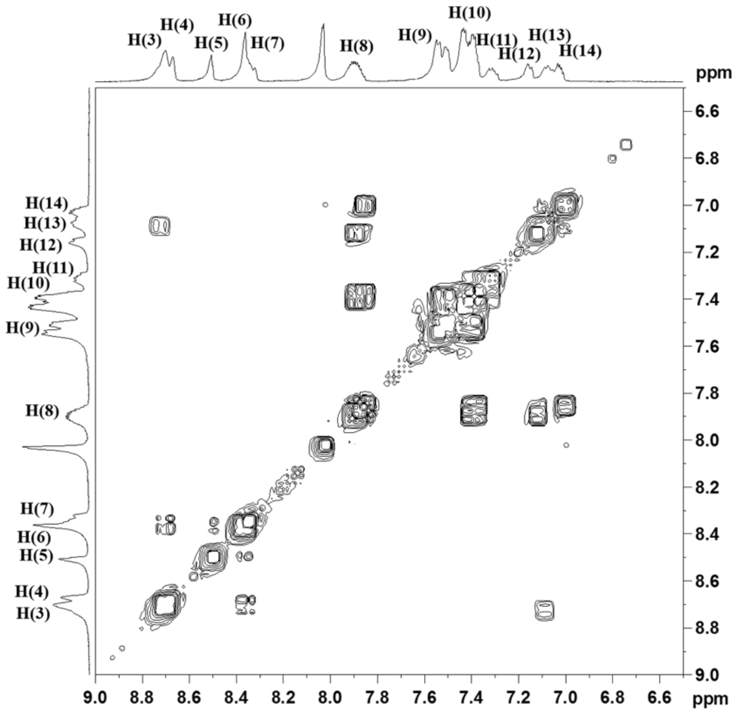

b

Figure S26. Full view (a) and expanded view (b) of the 2D COSY NMR spectrum of $\mathbf{2}^{2+}\cdot 2PF_6^-$ (2.00×10^{-2} M) collected in DMF-d_7 at 278K (500 MHz)

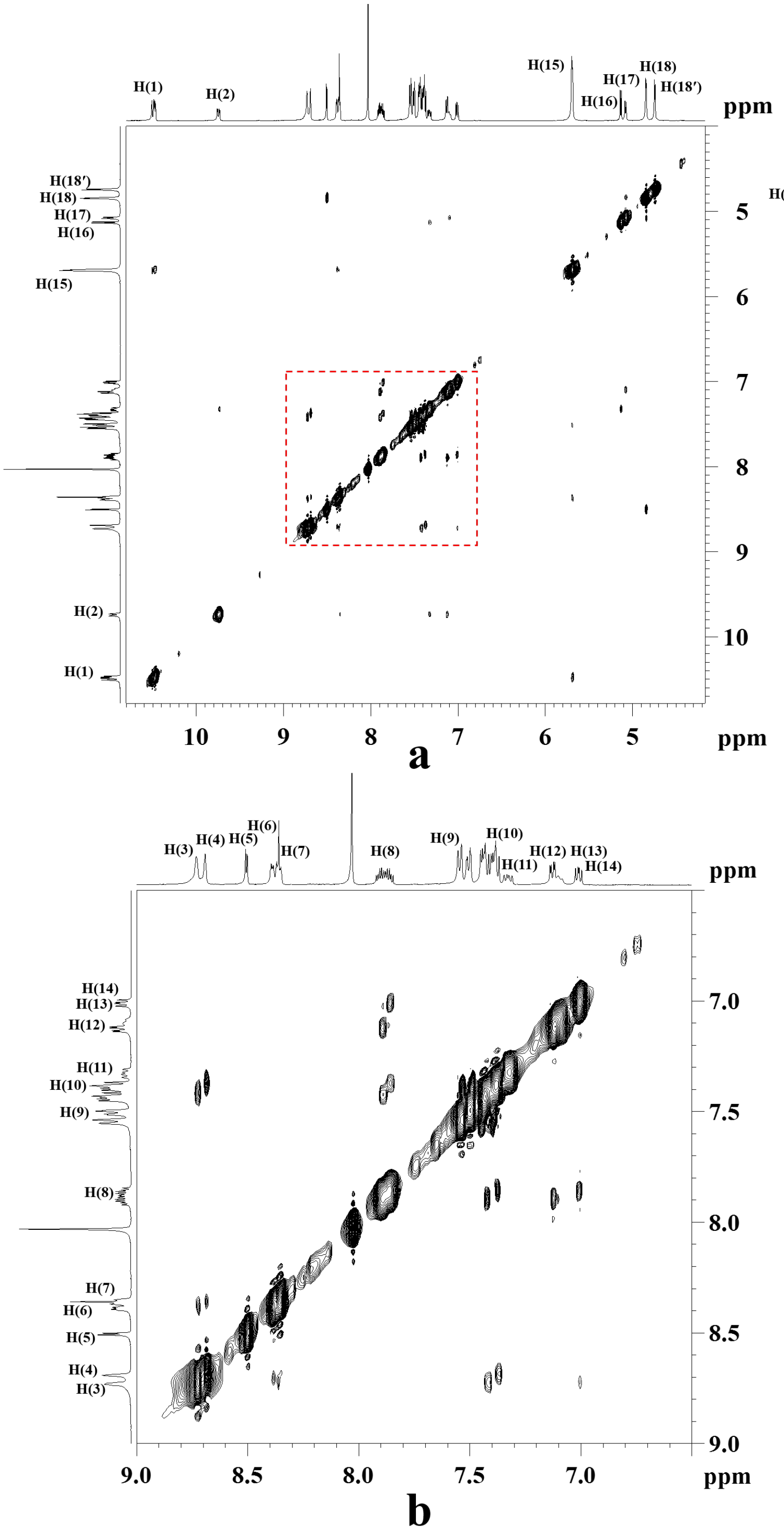

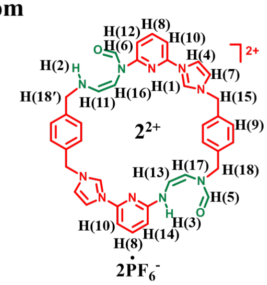


```
Current Data Parameters
NAME        SJ-TM12-DMF2
EXPNO                220
PROCNO                 1

F2 - Acquisition Parameters
Date_           20151013
Time               11.49
INSTRUM            spect
PROBHD   5 mm PABBO BB/
PULPROG      noesygpphpp
TD                  2048
SOLVENT              DMF
NS                     8
DS                    16
SWH             5747.126 Hz
FIDRES          2.806214 Hz
AQ             0.1781760 sec
RG                 64.12
DW                87.000 usec
DE                  6.50 usec
TE                 278.4 K
D0            0.00007427 sec
D1            2.00000000 sec
D8            0.30000001 sec
D11           0.03000000 sec
D12           0.00002000 sec
D16           0.00020000 sec
IN0           0.00017400 sec

======== CHANNEL f1 ========
SFO1         500.1332188 MHz
NUC1                  1H
P1                 10.00 usec
P2                 20.00 usec
P17              2500.00 usec
PLW1         10.00000000 W
PLW10         1.47930002 W

====== GRADIENT CHANNEL =====
GPNAM[1]      SMSQ10.100
GPZ1               40.00 %
P16              1000.00 usec

F1 - Acquisition parameters
TD                   256
SFO1            500.1332 MHz
FIDRES         22.449713 Hz
SW                11.491 ppm
FnMODE        States-TPPI

F2 - Processing parameters
SI                  1024
SF           500.1300000 MHz
WDW                QSINE
SSB                    2
LB                     0 Hz
GB                     0
PC                  1.00

F1 - Processing parameters
SI                  1024
MC2           States-TPPI
SF           500.1300000 MHz
WDW                QSINE
SSB                    2
LB                     0 Hz
GB                     0
```

Figure S27. Full view (a) and expanded view (b) of the 2D NOESY NMR spectrum of $\mathbf{2}^{2+}\cdot 2PF_6^-$ (2.00×10^{-2} M) collected in DMF-d_7 at 278K (500 MHz).

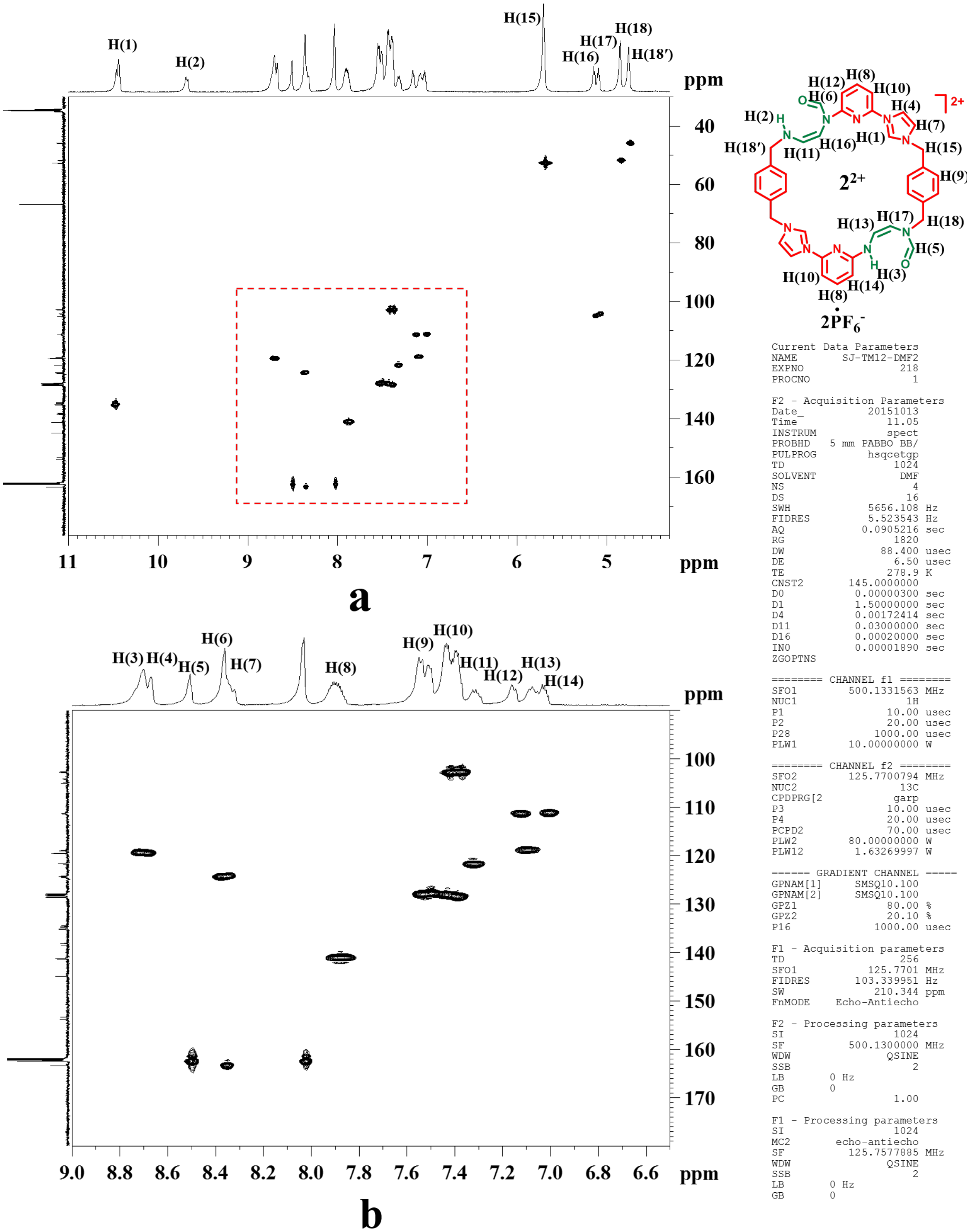

Figure S28. Full view (a) and expanded view (b) of the HSQC NMR spectrum of $\mathbf{2}^{2+}\cdot 2PF_6^-$ (2.00 × 10^{-2} M) collected in DMF-d_7 at 278K (500 MHz).

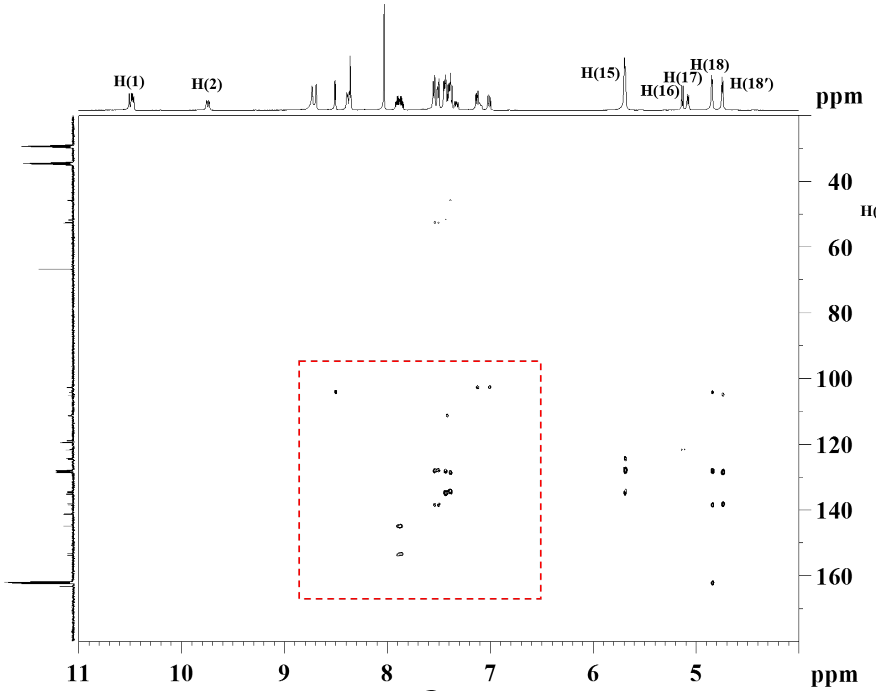

a

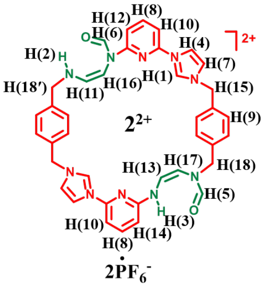


```
Current Data Parameters
NAME       SJ-TM12-DMF2-20mm
EXPNO                    219
PROCNO                     1

F2 - Acquisition Parameters
Date_               20151013
Time                   11.33
INSTRUM                spect
PROBHD      5 mm PABBO BB/
PULPROG         hmbcgplpndqf
TD                      2048
SOLVENT                  DMF
NS                         4
DS                        16
SWH                 5656.108 Hz
FIDRES              2.761772 Hz
AQ                 0.1810432 sec
RG                      1820
DW                    88.400 usec
DE                      6.50 usec
TE                     277.9 K
CNST2            145.0000000
CNST13            10.0000000
D0                0.00000300 sec
D1                1.50000000 sec
D2                0.00344828 sec
D6                0.05000000 sec
D16               0.00020000 sec
IN0               0.00001890 sec

======== CHANNEL f1 ========
SFO1             500.1331563 MHz
NUC1                      1H
P1                     10.00 usec
P2                     20.00 usec
PLW1             10.00000000 W

======== CHANNEL f2 ========
SFO2             125.7700794 MHz
NUC2                     13C
P3                     10.00 usec
PLW2             80.00000000 W

====== GRADIENT CHANNEL =====
GPNAM[1]         SMSQ10.100
GPNAM[2]         SMSQ10.100
GPNAM[3]         SMSQ10.100
GPZ1                   50.00 %
GPZ2                   30.00 %
GPZ3                   40.10 %
P16                  1000.00 usec

F1 - Acquisition parameters
TD                       128
SFO1                125.7701 MHz
FIDRES            206.679901 Hz
SW                   210.344 ppm
FnMODE                    QF

F2 - Processing parameters
SI                      2048
SF               500.1300000 MHz
WDW                     SINE
SSB        0
LB         0 Hz
GB         0
PC                      1.00

F1 - Processing parameters
SI                      1024
MC2                       QF
SF               125.7577885 MHz
WDW                     SINE
SSB        0
LB         0 Hz
GB         0
```

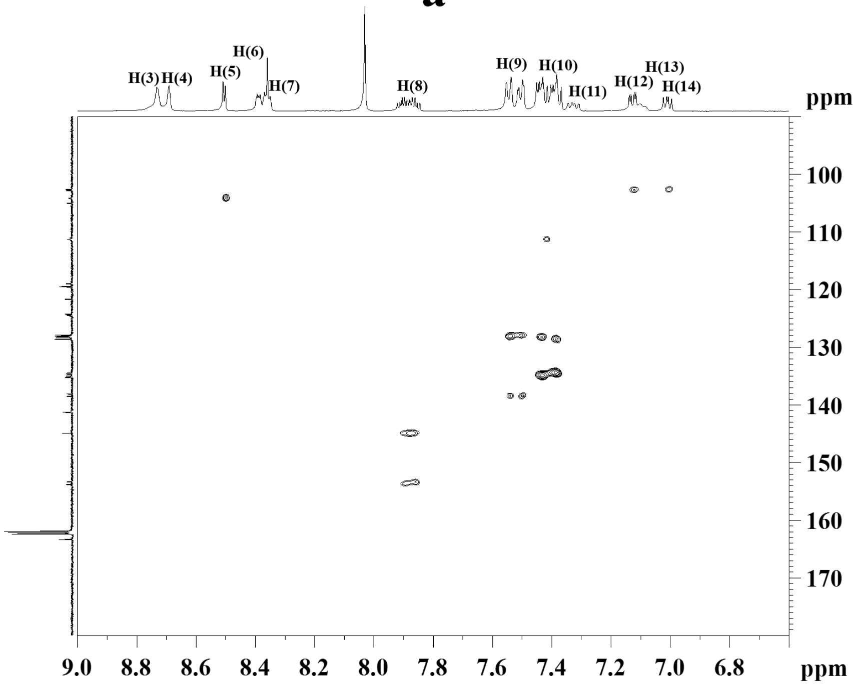

b

Figure S29. Full view (a) and expanded view (b) of the HMBC NMR spectrum of $\mathbf{2}^{2+}\cdot 2PF_6^-$ (2.00×10^{-2} M) collected in DMF-d_7 at 278K (500 MHz).

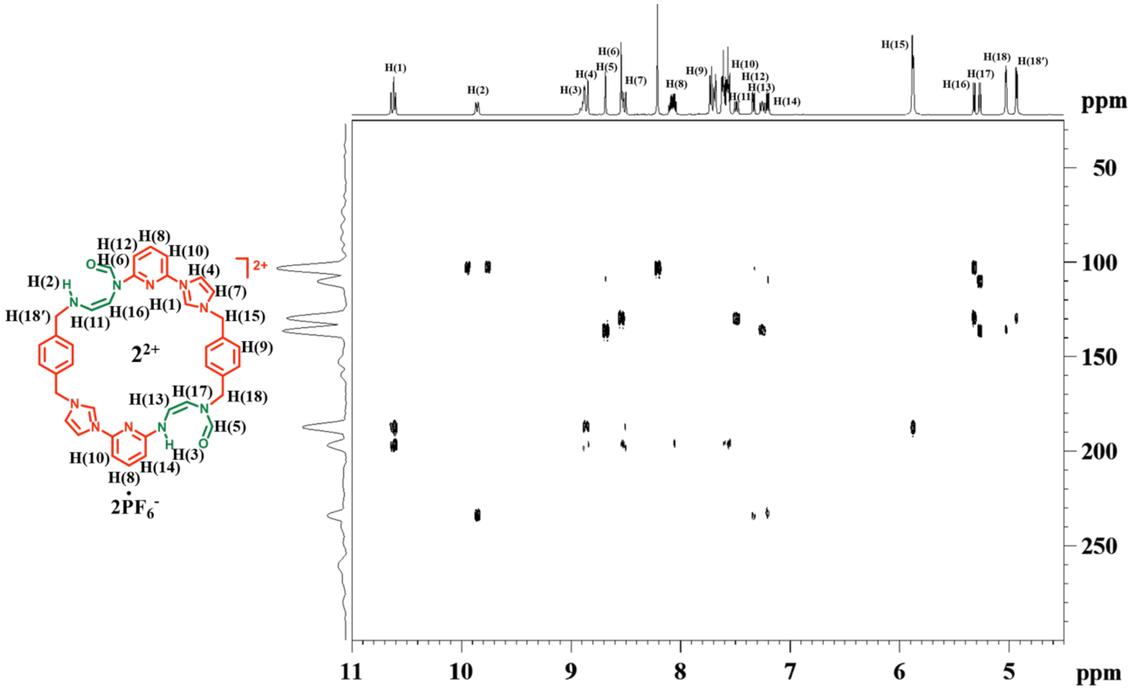

Figure S30. Full view of the ^{1}H,^{15}N-HMBC NMR spectrum of $\mathbf{2}^{2+}\cdot 2PF_6^-$ (2.00×10^{-2} M) collected in DMF-d_7 at 298K (500 MHz).

Characterization of the by-product: *Cis*-cyclo[2]((Z)-*N*-(2-((6-(1*H*-imidazol-1-yl)pyridin-2-yl)amino)vinyl)formamide)[2](1,4-bismethylbenzene)hexafluorophosphate [$3^{2+}\cdot2PF_6^-$]:

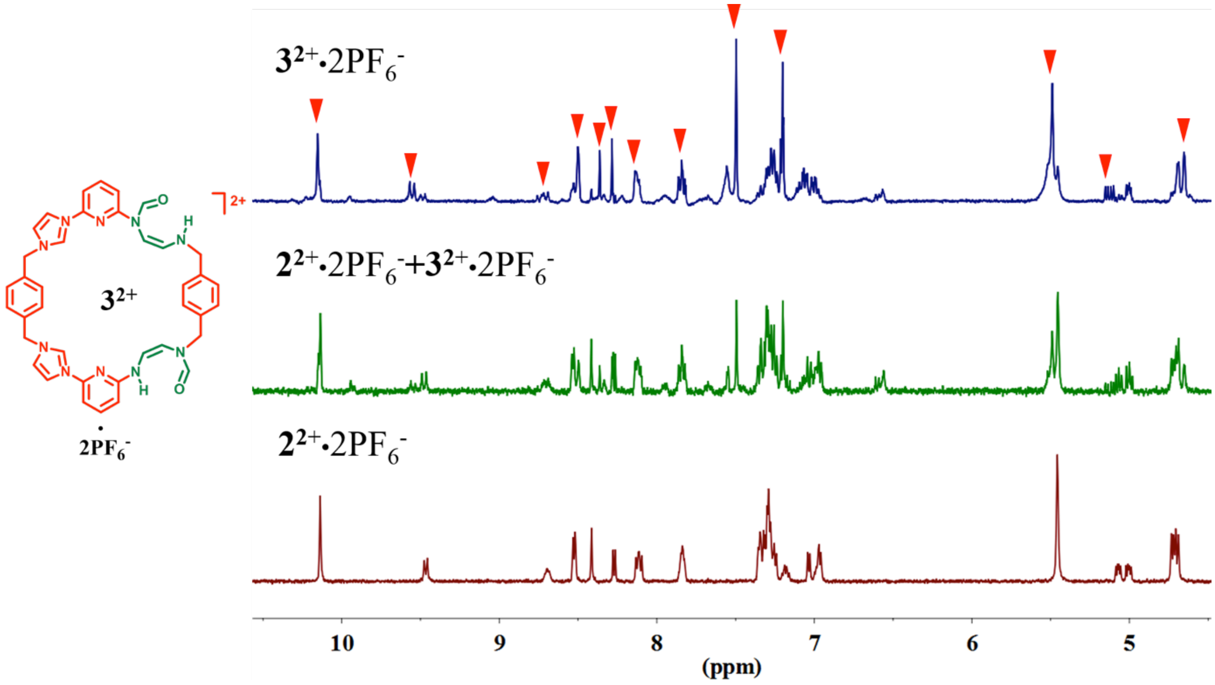

Figure S31. ^{1}H NMR spectrum of impure $\mathbf{3}^{2+}\cdot2PF_6^-$, the crude product containing $\mathbf{2}^{2+}\cdot2PF_6^-$and $\mathbf{3}^{2+}\cdot2PF_6^-$, and pure $\mathbf{2}^{2+}\cdot2PF_6^-$ collected in DMSO-d_6 at 300K (400 MHz). The signals labeled with "▼" indicate possible protons on $\mathbf{3}^{2+}\cdot2PF_6^-$.

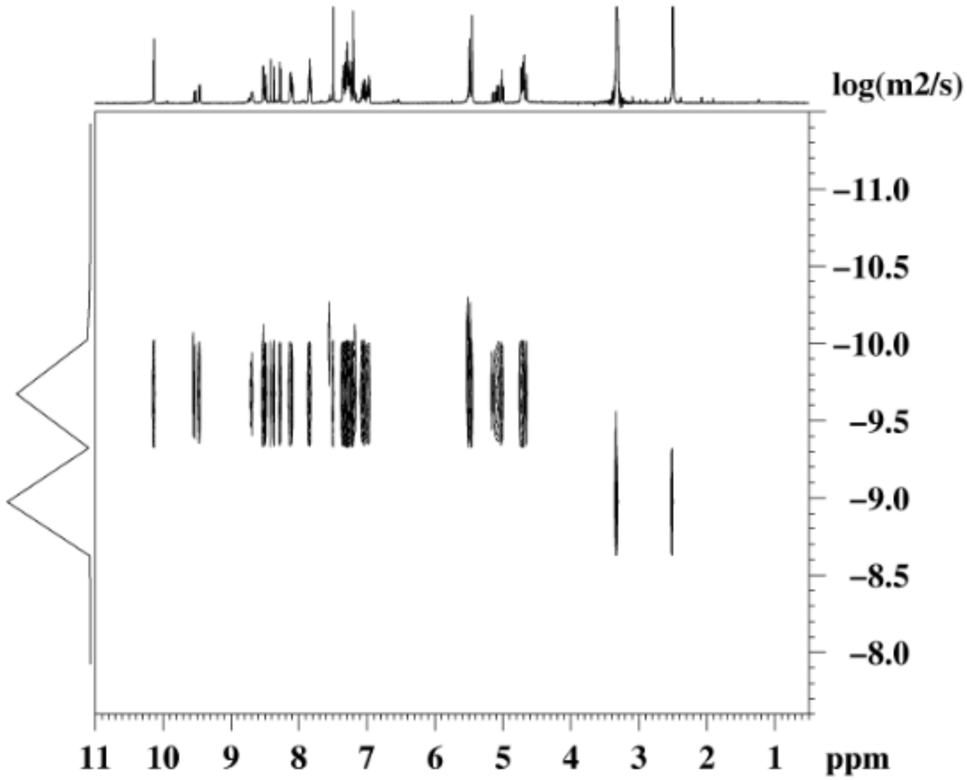

Figure S32. 2D DOSY NMR spectrum of crude product containing $\mathbf{2}^{2+}\cdot 2PF_6^-$ and $\mathbf{3}^{2+}\cdot 2PF_6^-$ (overall concentration as 5×10^{-3} M) collected in DMSO-d_6 at 300K (600 MHz).

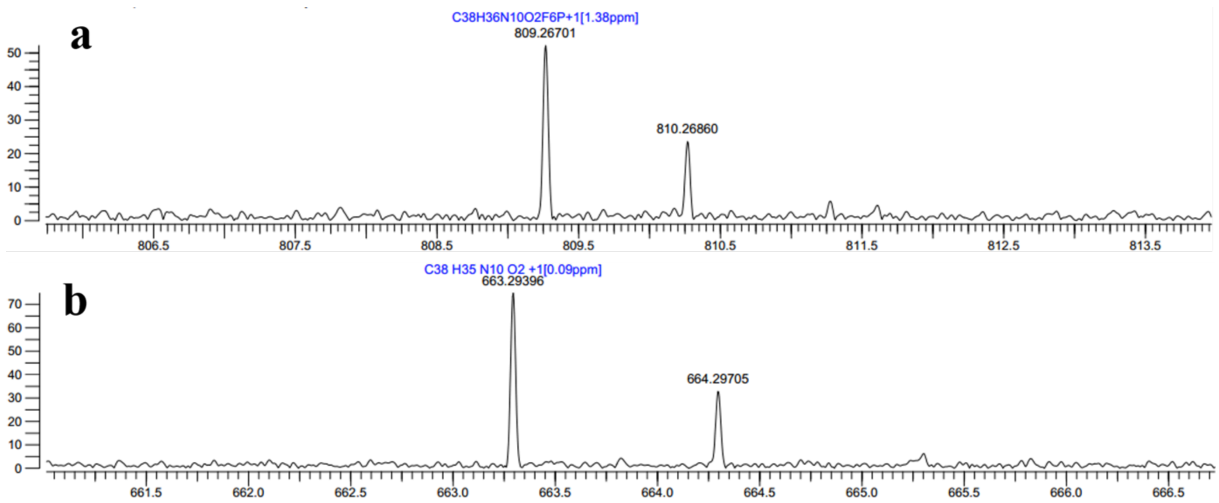

Figure S33. Expanded view ((a) and (b)) of ESI high resolution mass spectrum of $\mathbf{3}^{2+}\cdot 2PF_6^-$ generated from the single crystal sample used in X-ray diffraction analysis ([$\mathbf{3}^{2+}\cdot 2PF_6^-\cdot CH_3CN$]).

***Section S3:** Single crystal X-ray diffraction analyses of [**2**$^{2+}$·2PF$_6^-$·4dioxane], [**2**$^{2+}$·2PF$_6^-$·CH$_3$CN·H$_2$O] and [**3**$^{2+}$·2PF$_6^-$·CH$_3$CN]*

Table S2. Collects X-ray crystallographic data for [**2**$^{2+}$·2PF$_6^-$·4dioxane], [**2**$^{2+}$·2PF$_6^-$·CH$_3$CN·H$_2$O], and [**3**$^{2+}$·2PF$_6^-$·CH$_3$CN].

	**2**$^{2+}$·2PF$_6^-$·4dioxane	**2**$^{2+}$·2PF$_6^-$·CH$_3$CN ·H$_2$O	**3**$^{2+}$·2PF$_6^-$·CH$_3$CN
CCDC No.	1433724	1433726	1433725
description	prism	prism	prism
color	yellow	yellow	yellow
From solution	dioxane /acetonitrile	water /acetonitrile	water /acetonitrile
empirical formula	$C_{54}H_{68}F_{12}N_{10}O_{10}P_2$	$C_{40}H_{39}F_{12}N_{11}O_3P_2$	$C_{40}H_{39}F_{12}N_{11}O_2P_2$
Mr	1307.12	1011.76	995.76
crystal size (mm^3)	0.14 × 0.09 × 0.07	0.37 × 0.10 × 0.09	0.49× 0.07 × 0.06
crystal system	Triclinic	Monoclinic	Monoclinic
space group	P-1	C2/c	P2(1)/n
a [Å]	10.032(2)	28.552(6)	11.353(2)
b [Å]	10.288(2)	13.685(3)	20.065(4)
c [Å]	15.459(3)	22.641(5)	19.117(4)
α [deg]	80.45(3)	90.00	90.00
β [deg]	80.06(3)	103.63(3)	98.58(3)
γ[deg]	80.81(3)	90.00	90.00
V/ [Å^3]	1535.6(5)	8598(3)	4305.8(15)
d/[g/cm^3]	1.413	1.563	1.536
Z	1	8	4
T [K]	173(2)	153(2)	153(2)
R1, wR2 $I > 2ó(I)$	0.0758 0.1585	0.0798 0.1651	0.0804 0.1687
R1, wR2 (all data)	0.0846 0.1643	0.1000 0.1786	0.0985 0.1791
quality of fit	1.011	1.002	1.020

Single-crystal X-ray structure of [2^{2+}·$2PF_6^-$·4dioxane]:

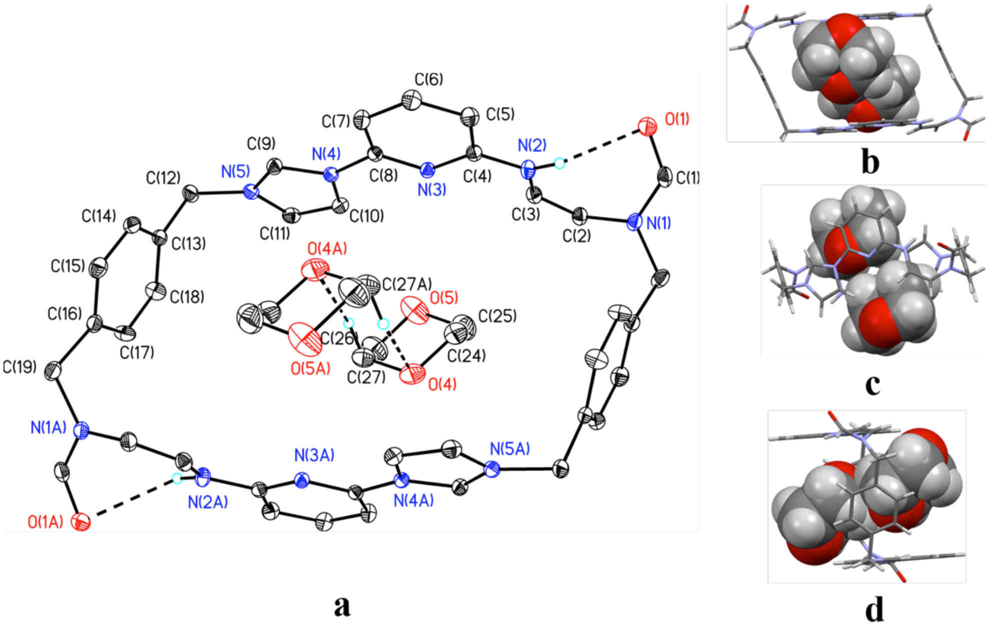

Figure S34. Top view of $\mathbf{2}^{2+}$ (a) in ellipsoid form, as well as its top view (b), side view (c), and the front view (d) in stick form. Here, $\mathbf{2}^{2+}$ adopts a "box" conformation containing two dioxanes in the final structure [$\mathbf{2}^{2+}$·$2PF_6^-$·4dioxane]. Displacement ellipsoids are scaled to the 30% probability level. Some or all of the counter anions, solvent molecules and hydrogen atoms have been omitted for clarity. The symmetry transformation invoked by the additional "A" letter in the atom labels on macrocycle $\mathbf{2}^{2+}$ or dioxane is (1-x, 1-y, 1-z). The possible O…π interaction was characterized with selected atomic distances between $\mathbf{2}^{2+}$ and dioxane are listed as below. Selected interatomic distances [Å]: O(4A)…N(4) 3.012(4), O(4A)…N(3) 3.754(4), O(4A)…N(5) 3.794(4), O(4A)…C(7) 3.475(5), O(4A)…C(8) 3.163(4), O(4A)…C(9) 3.197(5), O(4A)…C(10) 3.577(4). Intramolecular hydrogen bonds of $\mathbf{2}^{2+}$ was noted via selected interatomic distances [Å]: O(1)…N(2) 2.951; and selected bond angles: N(2)-H(2B)…O(1) 120.9º. Possible intermolecular hydrogen bonds between two dioxane molecules with selected interatomic distances [Å]: O(4)…C(27A) 3.610; and selected bond angles: C(27A)-H(27AA)…O(4) 141.2º.

Single-crystal X-ray structure of [$\mathbf{2}^{2+}$·$2PF_6^-$·CH_3CN·H_2O]:

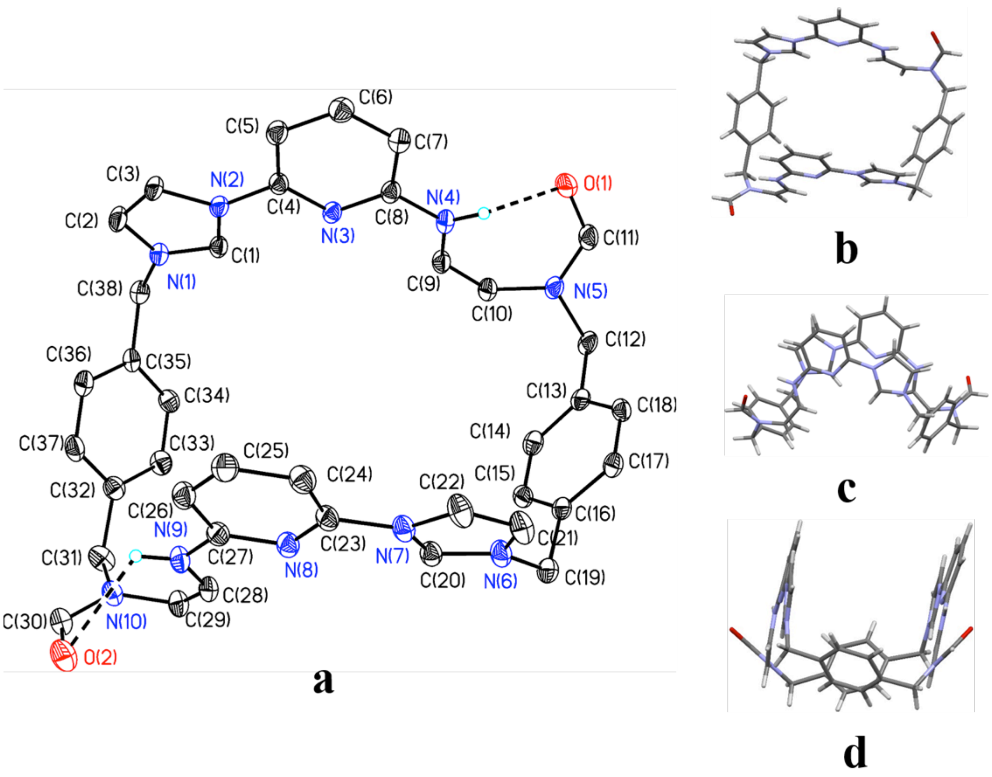

Figure S35. Top view of $\mathbf{2}^{2+}$ (a) shown in ellipsoid form, as well as top (b), side (c), and front (d) views in the stick form. Here $\mathbf{2}^{2+}$ adopts a "clip" conformation in the final structure [$\mathbf{2}^{2+}$·$2PF_6^-$·CH_3CN·H_2O]. Displacement ellipsoids are scaled to the 25% probability level. Some or all of the counter anions, solvent molecules and hydrogen atoms have been omitted for clarity. Possible intramolecular hydrogen bonds are characterized with selected interatomic distances [Å]: O(1)…N(4) 2.720(4), O(2)…N(9) 3.061(5); selected bond angles: N(4)-H(4A)…O(1) 126.8º, N(9)-H(9B)…O(2) 118.9º.

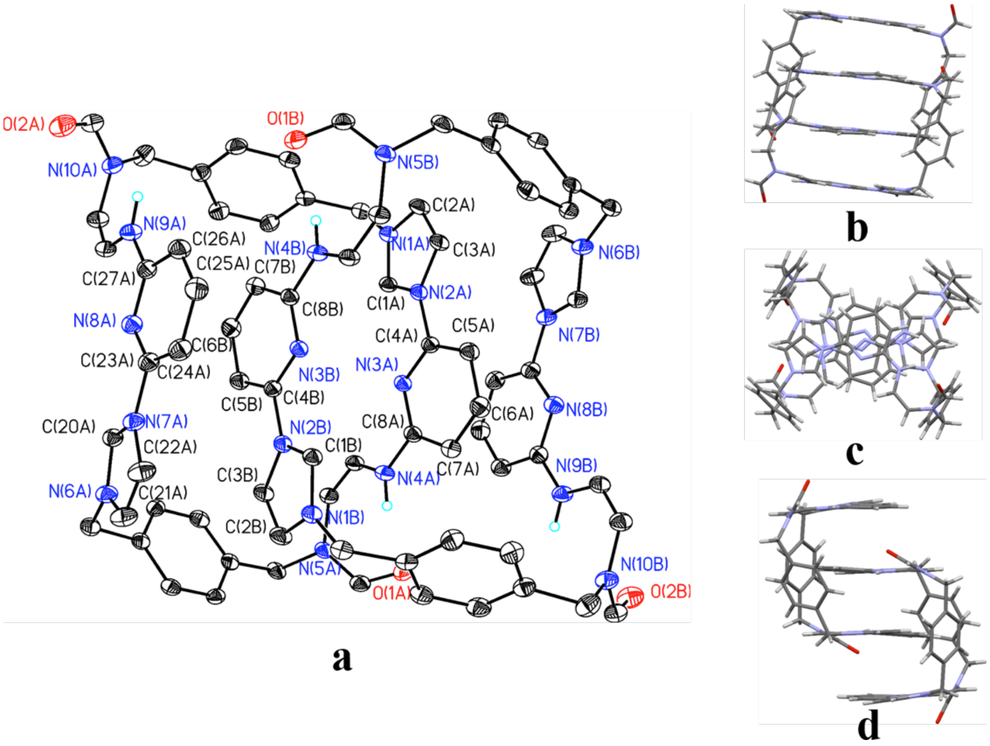

Figure S36. Top view of $[\mathbf{2}^{2+}]_2$ (a) shown in ellipsoid form, as well as top (b), side (c), and the front views (d) in stick form. Here, $\mathbf{2}^{2+}$ organizes into a head-to-tail racemic dimer in the final structure $[\mathbf{2}^{2+}\cdot 2PF_6^{-}\cdot CH_3CN\cdot H_2O]$. Displacement ellipsoids are scaled to the 25% probability level. Some or all of the counter anions, solvent molecules and hydrogen atoms have been omitted for clarity. The symmetry transformation invoked by the additional "A" letter and "B" letter in the atom labels on the macrocycle $\mathbf{2}^{2+}$ are (x, y, z) and (1.5-x, 1/2-y, 1-z). Possible intramolecular π…π interaction: Selected interatomic distances [Å]: N(3B)…C(24A) 3.666(5), N(3B)…C(23A) 3.741(5), C(8B)…C(26A) 3.492(6), C(8B)…C(27A) 3.565(6), C(6B)…N(8A) 3.503(5), N(3B)…N(3A) 3.304(4), C(8A)…N(3B) 3.800(5), N(3B)…C(4A) 3.441(5), C(4B)…C(8A) 3.712(6), C(5B)…C(23A) 3.788(6), C(4B)…C(23A) 3.580(6), N(7A)…N(2B) 3.697(5), C(21A)…C(2B) 3.787(7), C(21A)…C(3B) 3.728(6), N(6A)…C(3B) 3.762(6).

Single-crystal X-ray structure of [3^{2+}·$2PF_6^{-}$·CH_3CN]:

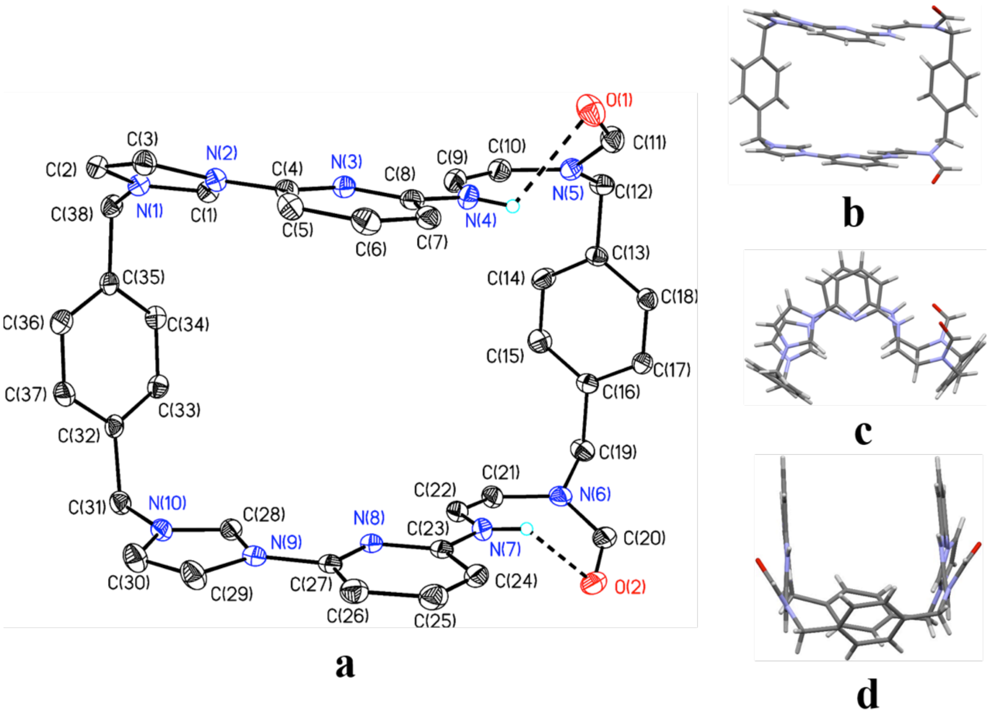

Figure S37. Top view of $\mathbf{3}^{2+}$ (a) in ellipsoid form, as well as top (b), side (c), and front views (d) in stick form. Here, $\mathbf{3}^{2+}$ adopts a "clip" conformation in the final structure [$\mathbf{3}^{2+}$·$2PF_6^{-}$·CH_3CN]. Displacement ellipsoids are scaled to the 25% probability level. Some or all of the counter anions, solvent molecules and hydrogen atoms have been omitted for clarity. Possible intramolecular hydrogen bonds are characterized with selected interatomic distances [Å]: O(1)…N(4) 2.751(5), O(2)…N(7) 2.690(5); selected bond angles: N(4)-H(4A)…O(1) 112.1º, N(7)-H(7B)…O(2) 124.1º.

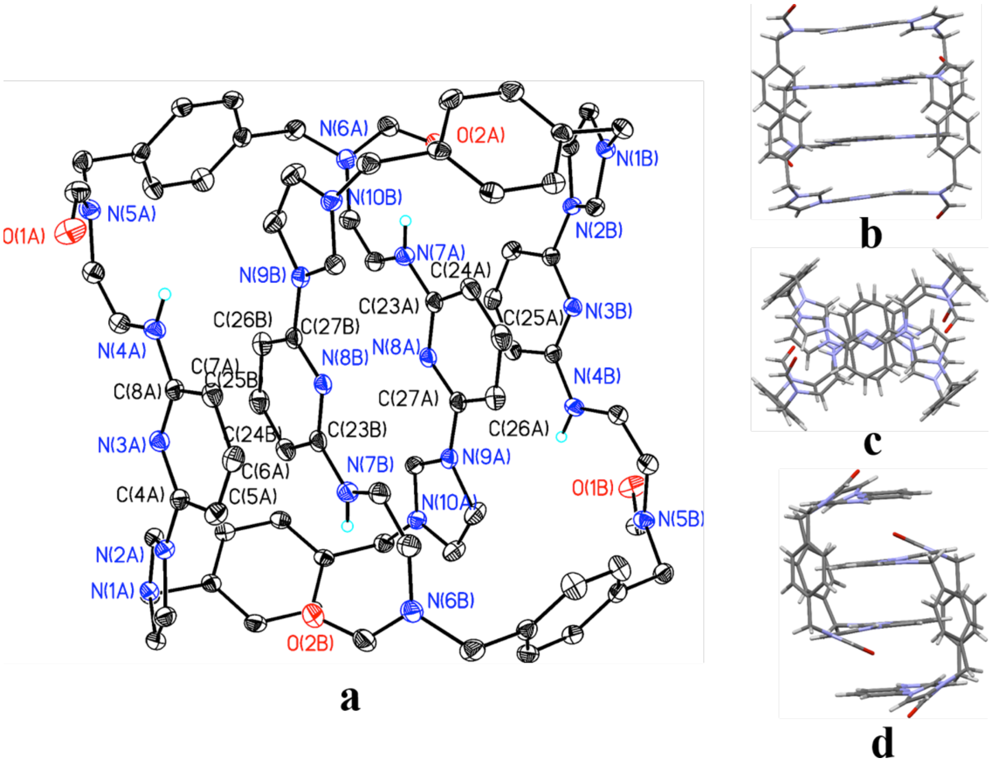

Figure S38. Top view of $[\mathbf{3}^{2+}]_2$ (a) in ellipsoid form, as well as top (b), side (c), and front views (d) in stick form. Here, $\mathbf{3}^{2+}$ organizes into a head-to-tail racemic dimer in the final structure $[\mathbf{3}^{2+}\cdot 2PF_6^-\cdot CH_3CN]$. Displacement ellipsoids are scaled to the 25% probability level. Some or all of the counter anions, solvent molecules and hydrogen atoms have been omitted for clarity. The symmetry transformation invoked by the additional "A" letter and "B" letter in the atom labels on the macrocycle $\mathbf{3}^{2+}$ are (x, y, z) and (1-x, 1-y, 1-z). Possible intramolecular π…π interaction: selected interatomic distances [Å]: N(8B)…N(3A) 3.681(5), N(8B)…C(7A) 3.491(5), N(8B)…C(8A) 3.405(5), C(23B)…C(5A) 3.792(6), C(23B)…C(4A) 3.552(6), C(24B)…C(4A) 3.791(5), C(24B)…N(3A) 3.664(5), C(27B)…C(8A) 3.438(5), N(8)…N(8B) 3.307(4), N(8B)…C(23A) 3.496(5), N(8B)…C(27A) 3.415(5), N(8B)…C(26A) 3.743(5), C(23B)…C(27A) 3.368(6).

Section S4: *Solution binding studies and characterization of the host-guest complexes formed between [$\mathbf{2}^{2+}$·2PF$_6^-$] and dianionic guests 2,6-naphthalenedicarboxylate (**4**), 2,6-naphthalenedisulfonate (**5**) or 1,5-naphthalenedisulfonate (**6**)*

Solution binding studies and characterization of the host-guest complex formed between $\mathbf{2}^{2+}$ and dianionic guest 2,6-naphthalene dicarboxylate (4):

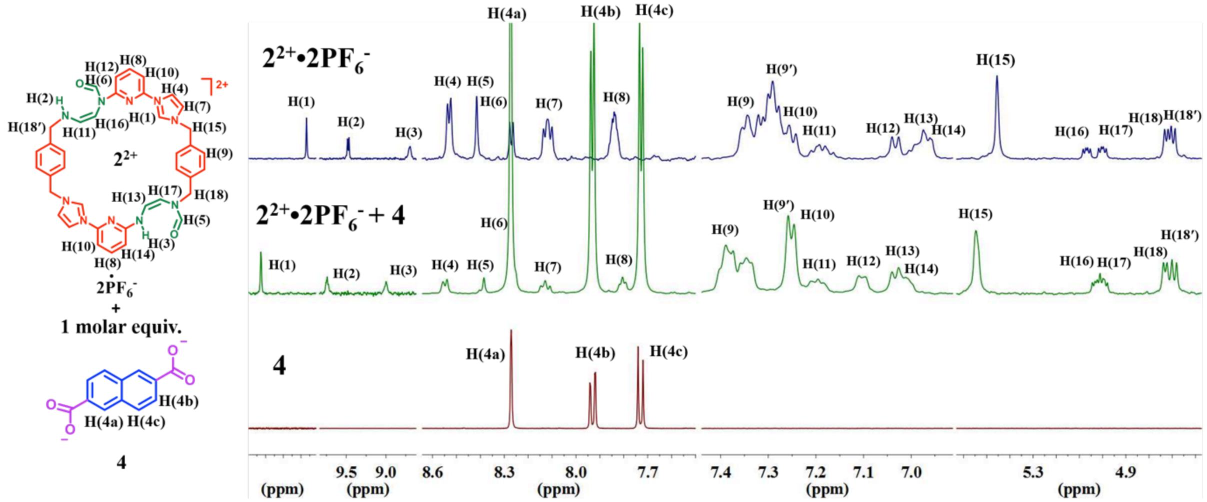

Figure S39. Expanded view of the ^{1}H NMR spectra of $\mathbf{2}^{2+}$·2PF$_6^-$ (1.00×10^{-3} M) recorded in the absence and presence of 1 molar equiv. of **4** in DMSO-d_6 at 300 K over the indicated spectral ranges (600 MHz).

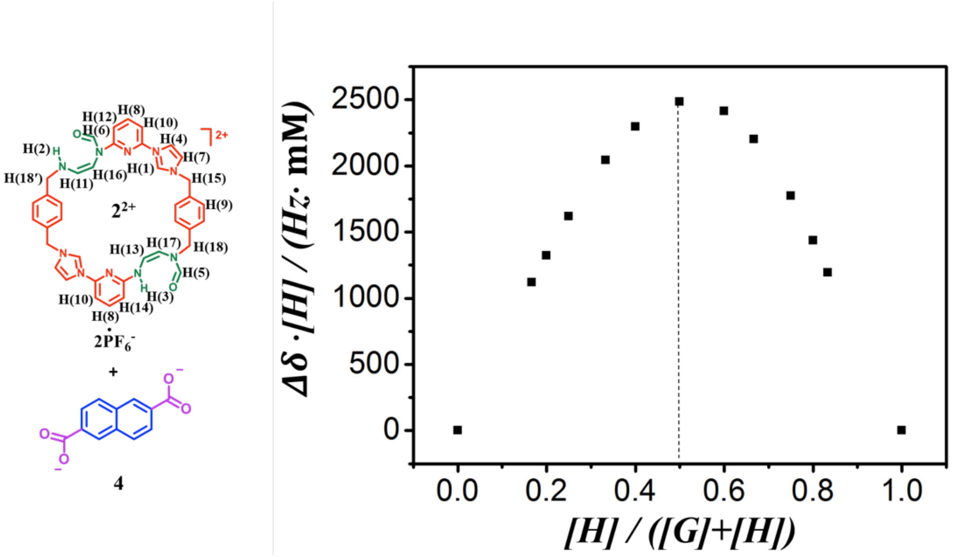

Figure S40. ^{1}H NMR Job-plots (600 MHz) corresponding to the binding between $\mathbf{2}^{2+}$·2PF$_6^-$ and **4** ([host] + [guest] = 2.00×10^{-2} M). The maximum value was found to be 0.5 at this concentration, a finding consistent with a 1:1 (host: guest) binding stoichiometry.[8]

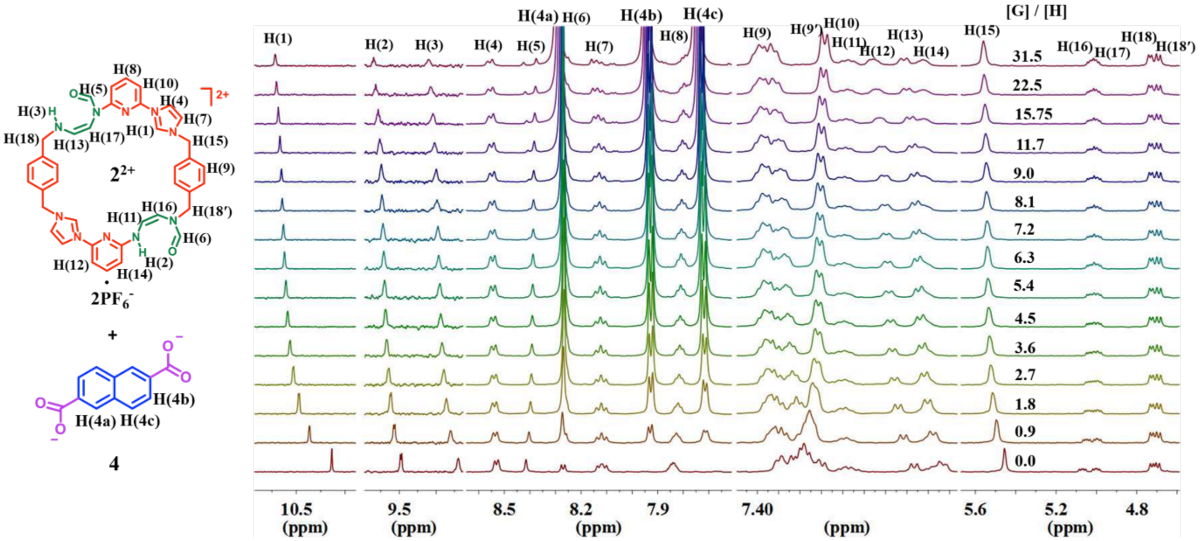

Figure S41. ^{1}H NMR spectroscopic titration of $\mathbf{2}^{2+}\cdot 2PF_6^-$ (1.00×10^{-3} M) with 2,6-naphthalenedicarboxylic acid in the presence of 2 molar equiv. of $TMA^+\cdot OH^-$ in DMSO-d_6 at 300K (600 MHz).

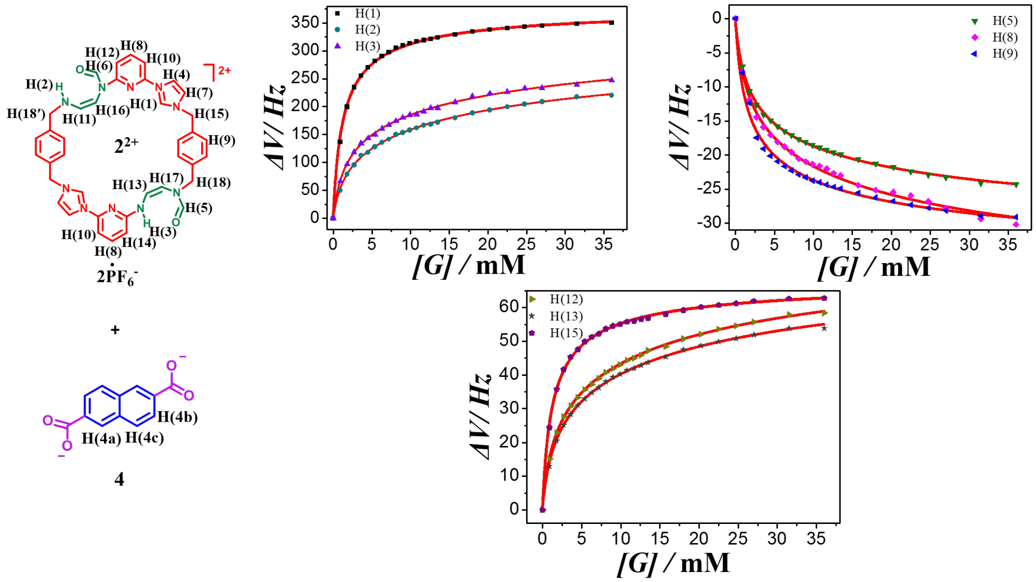

Figure S42. ^{1}H NMR binding isotherms corresponding to the interaction between $\mathbf{2}^{2+}\cdot 2PF_6^-$ and **4** in DMSO-d_6 at 300K. The chemical shift changes of (a) H(1), H(2), H(3) and (b) H(5), H(8), H(9) and (c) H(12), H(13), H(15) on $\mathbf{2}^{2+}$ were used for the calculation of Ka = $(2.0 \pm 0.1) \times 10^2$ M^{-1} using the Hyperquad 2003 program.[9] The chemical shift changes of the signals of other protons present in $\mathbf{2}^{2+}$ and **4** overlap and thus could not be accurately fit with either a Guassian or Lorentzian function. In other cases the change in chemical shifts were too small (less than 15.6 Hz); these signals were therefore not used in the calculation of the association constant.[10] The red dashed lines show the non-linear curve fit of the experimental data to the appropriate equation.

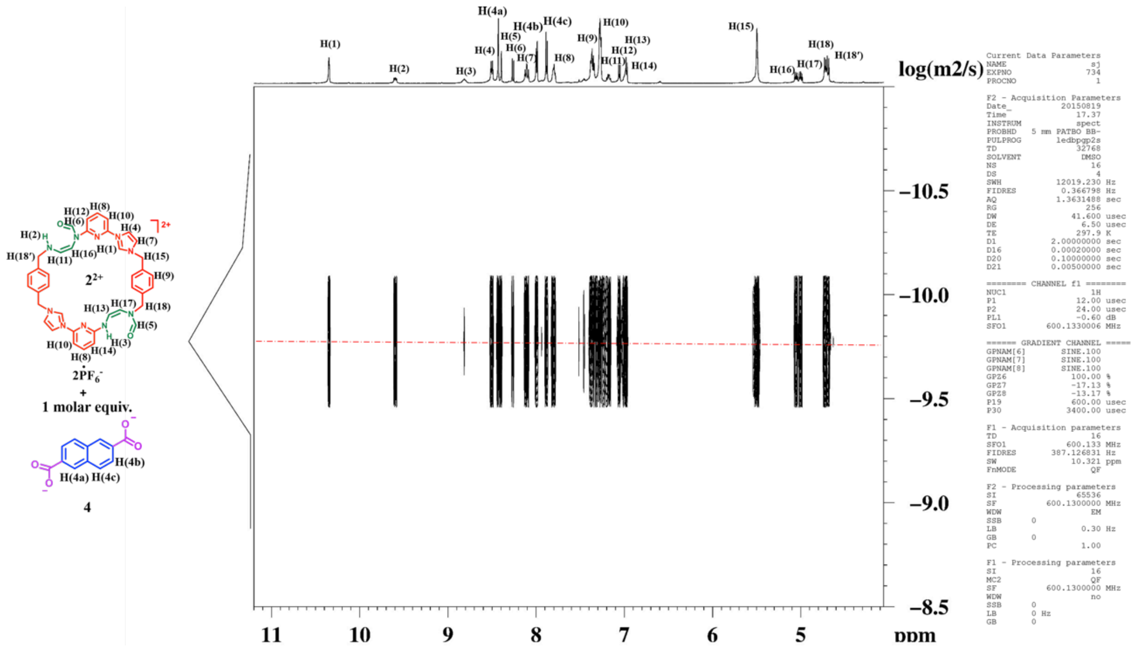

Figure S43. Full view of the 600 MHz 2D-DOSY NMR spectra of $\mathbf{2}^{2+}\cdot 2PF_6^-$ (2.00×10^{-2} M) recorded in the presence of 1 molar equiv. of 2,6-naphthalenedicarboxylic acid and 2 molar equiv. of $TMA^+\cdot OH^-$ collected in DMSO-d_6 at 300K.

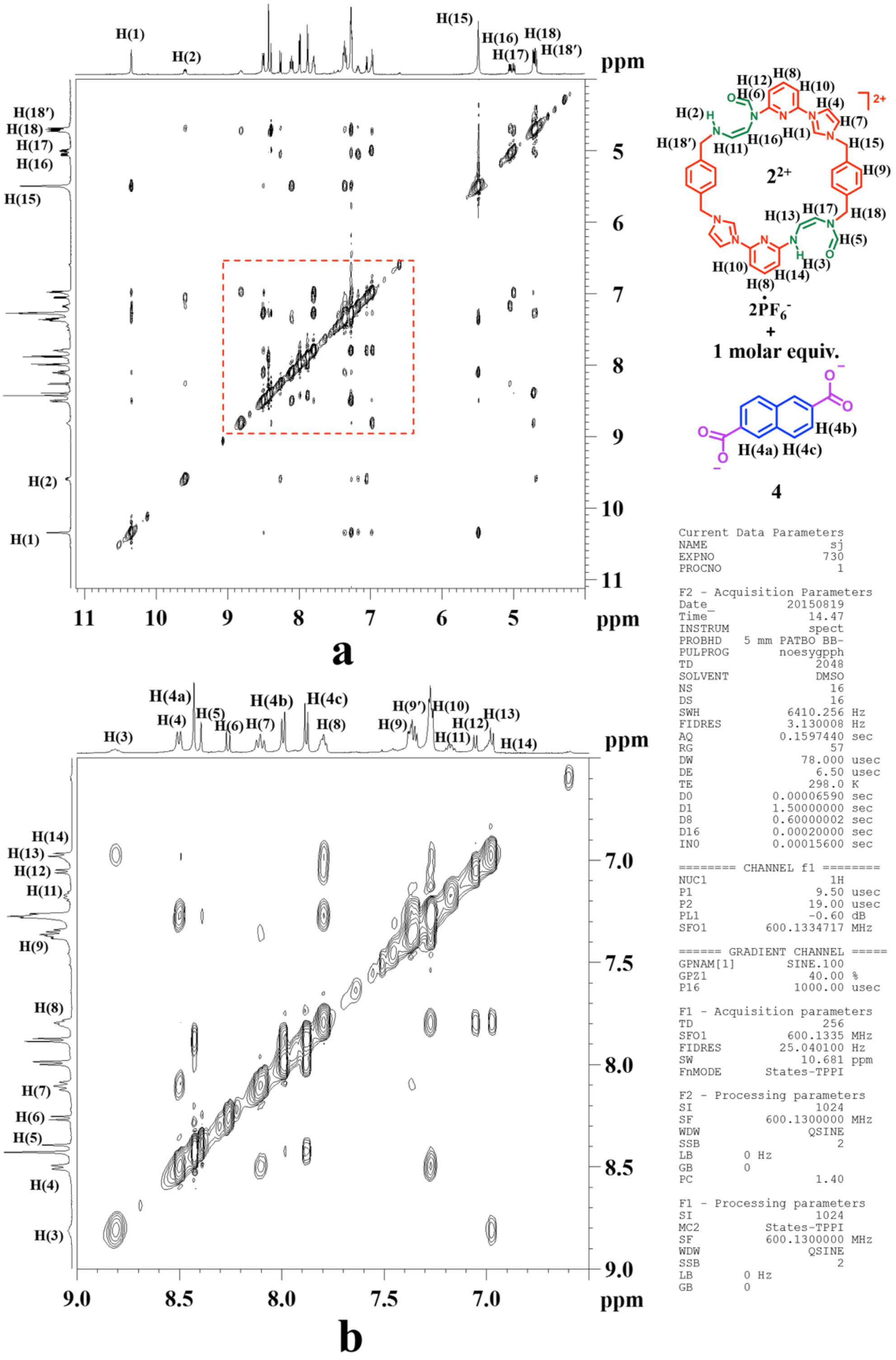

Figure S44. Full view (a) and expanded view (b) of the 600 MHz NOESY NMR spectrum of $\mathbf{2}^{2+}\cdot 2PF_6^-$ (2.0×10^{-2} M) recorded in the presence of 1 molar equiv. of 2,6-naphthalenedicarboxylic acid and 2 molar equiv. of $TMA^+\cdot OH^-$ collected in DMSO-d_6 at 300K.

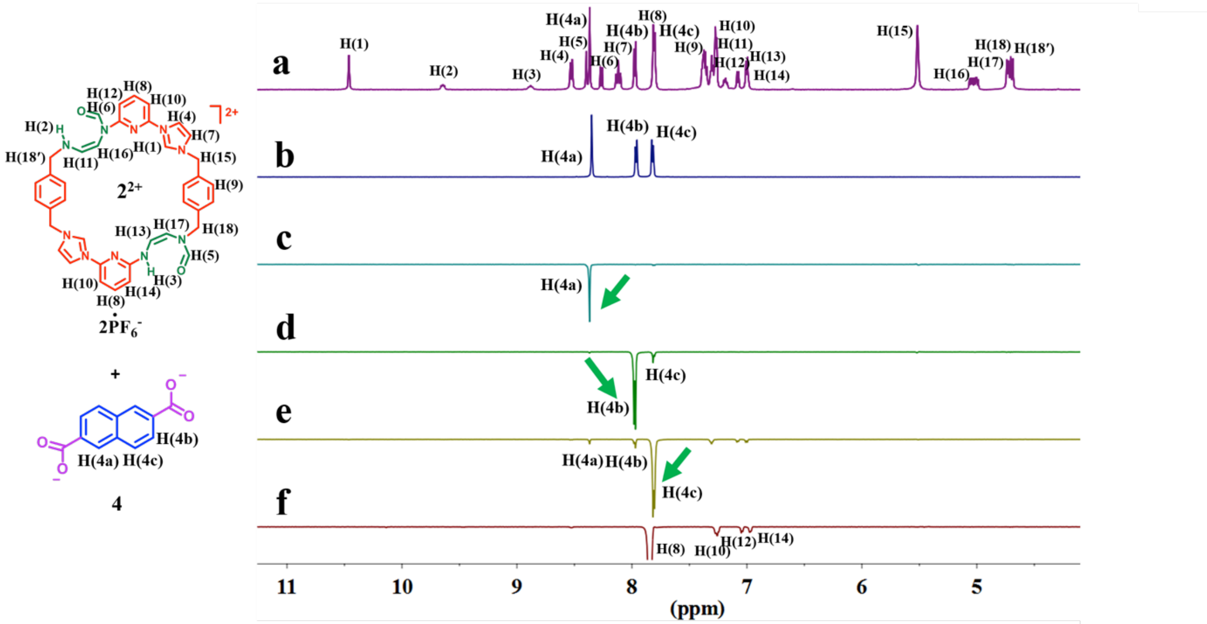

Figure S45. 600 MHz ^{1}H NMR and 1D NOE NMR spectra recorded in DMSO-d_6 at 300K: (a) ^{1}H NMR of $\mathbf{2}^{2+}\cdot 2PF_6^-$ in the presence of 1 molar equiv. of 2,6-naphthalenedicarboxylic acid and 2 molar equiv. of $TMA^+\cdot OH^-$ ([host] = [guest] = 1.00×10^{-2} M), (b) ^{1}H NMR of the 2,6-naphthalenedicarboxylic acid in the presence of 2 molar equiv. of $TMA^+\cdot OH^-$, (c-e) 1D-NOE NMR spectra of $\mathbf{2}^{2+}\cdot 2PF_6^-$ in the presence of 1 molar equiv. of 2,6-naphthalenedicarboxylic acid and 2 molar equiv. of $TMA^+\cdot OH^-$ ([host] = [guest] = 1.00×10^{-2} M). The green arrows indicate irradiation at the frequency of H(4a), H(4b) and H(4c) of **4**. Shown in (f) is the 1D NOE NMR spectrum of $\mathbf{2}^{2+}\cdot 2PF_6^-$ (1.00×10^{-2} M). The green arrow indicates irradiation at the frequency of H(8) of $\mathbf{2}^{2+}\cdot 2PF_6^-$.

Solution binding studies and characterization of the host-guest complex formed between 2^{2+} and dianionic guest 2,6-naphthalenedisulfonate (5):

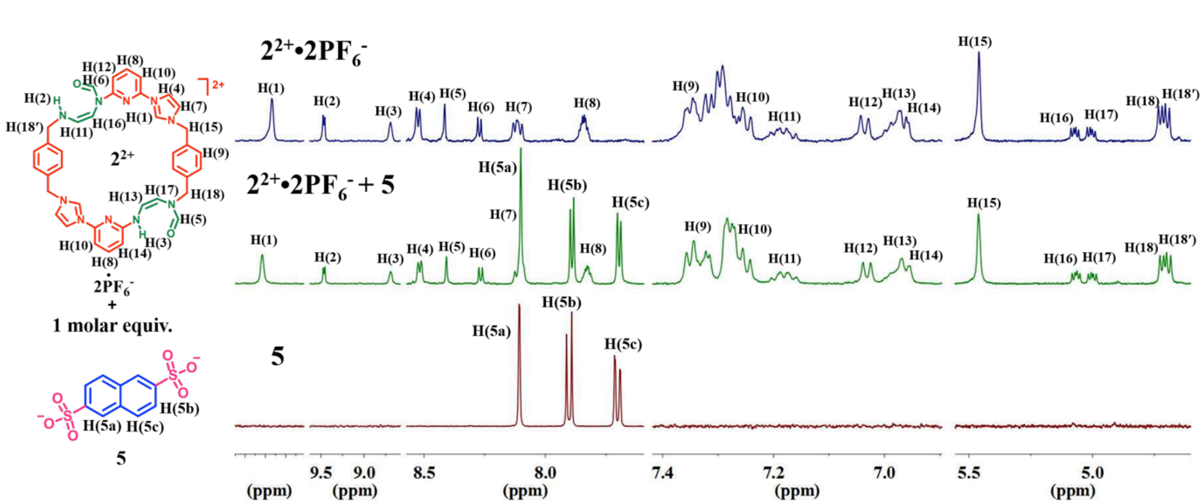

Figure S46. Expanded views of the ^{1}H NMR spectra of $\mathbf{2}^{2+}\cdot 2PF_6^-$ (1.00×10^{-3} M) recorded in the absence and presence of 1 molar equiv. of **5** in DMSO-d_6 at 300 K over the indicated spectral ranges (600 MHz).

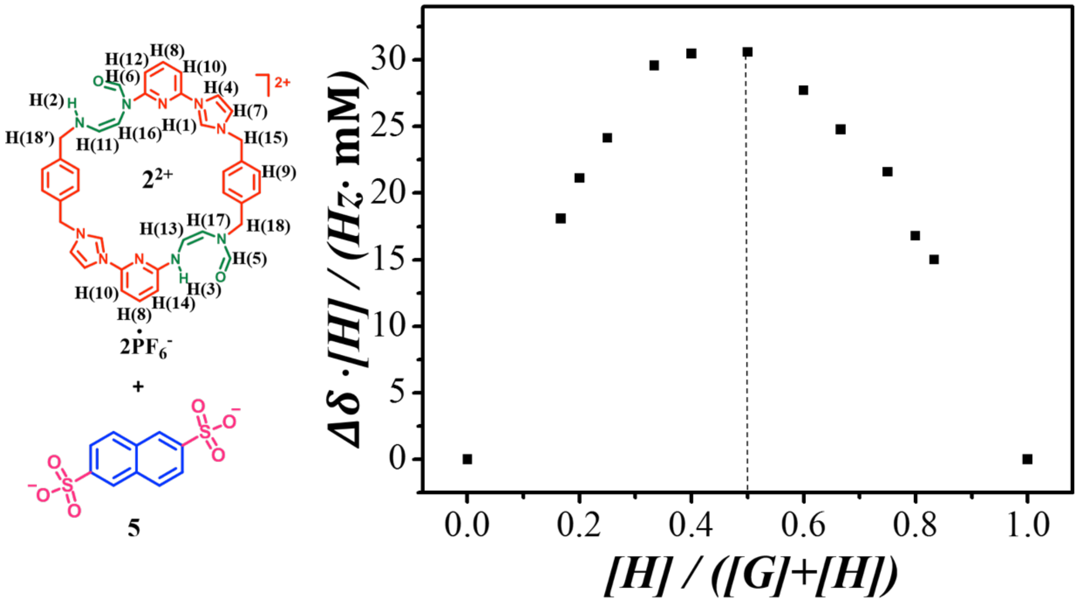

Figure S47. ^{1}H NMR Job-plots (600 MHz) associated with the binding between $\mathbf{2}^{2+}\cdot 2PF_6^-$ and **5** ([host] + [guest] = 1.00×10^{-2} M). The maximum value was determined to be 0.5 at this concentration, a finding consistent with a 1:1 (host: guest) binding stoichiometry.[8]

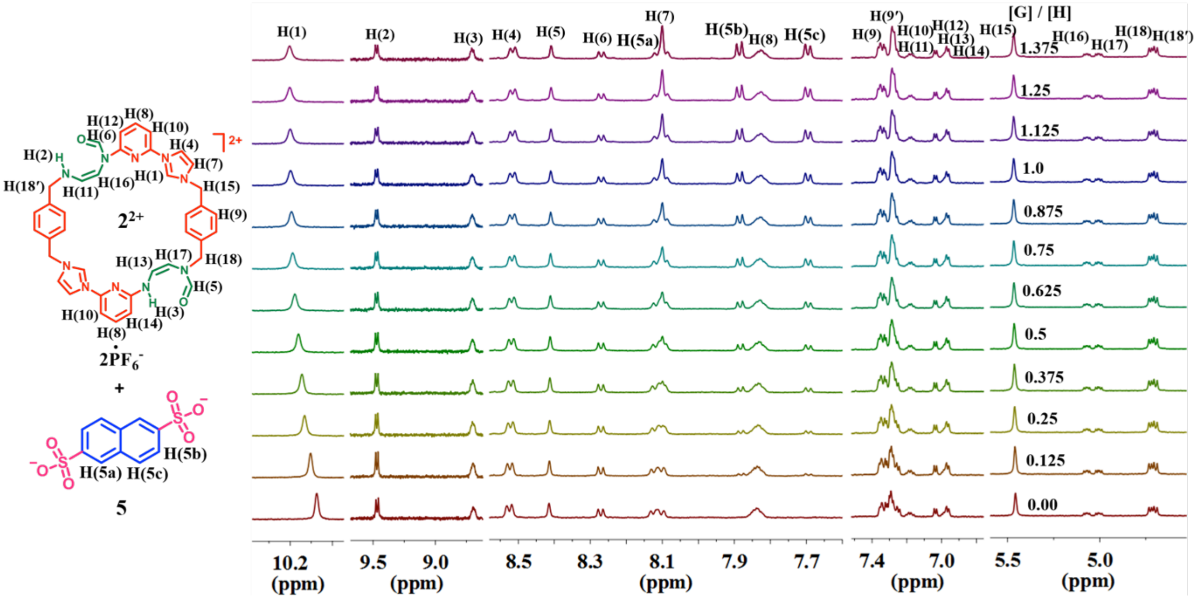

Figure S48. ^{1}H NMR spectroscopic titration of $\mathbf{2}^{2+}\cdot 2PF_6^-$ (1.00×10^{-3} M) with 2,6-naphthalenedisulfonic acid in the presence of 2 molar equiv. of $TMA^+\cdot OH^-$ collected in DMSO-d_6 at 300K (600 MHz).

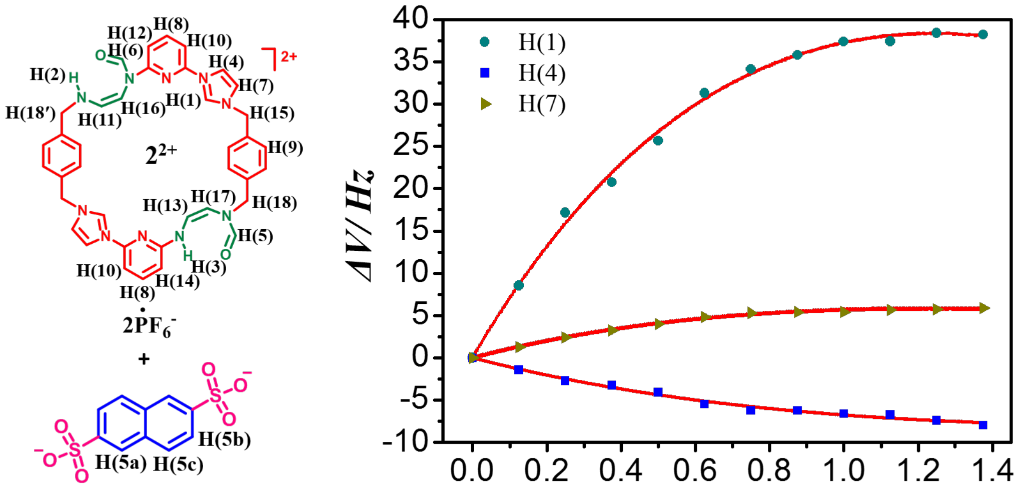

Figure S49. ^{1}H NMR binding isotherms that correspond to the interaction between $\mathbf{2}^{2+}\cdot 2PF_6^-$ and **5** collected in DMSO-d_6 at 300K. The chemical shift changes of (a) H(1), H(4) and H(7) on $\mathbf{2}^{2+}$ were used for the calculation of K_a = (1.7 ± 0.2) ×10^2 M^{-1} for [$\mathbf{2}^{2+}\cdot\mathbf{5}$] formation using the Hyperquad 2003 program.[9] The changes in chemical shift of other protons present in $\mathbf{2}^{2+}$ and **5** overlapped and thus could not be accurately fit with either a Guassian or Lorentzian function. Also in some cases the chemical shift change was too small (less than 8.0 Hz); these signals were therefore not used in the calculation of the association constant.[10] The red dashed lines show the non-linear curve fit of the experimental data to the appropriate equation.

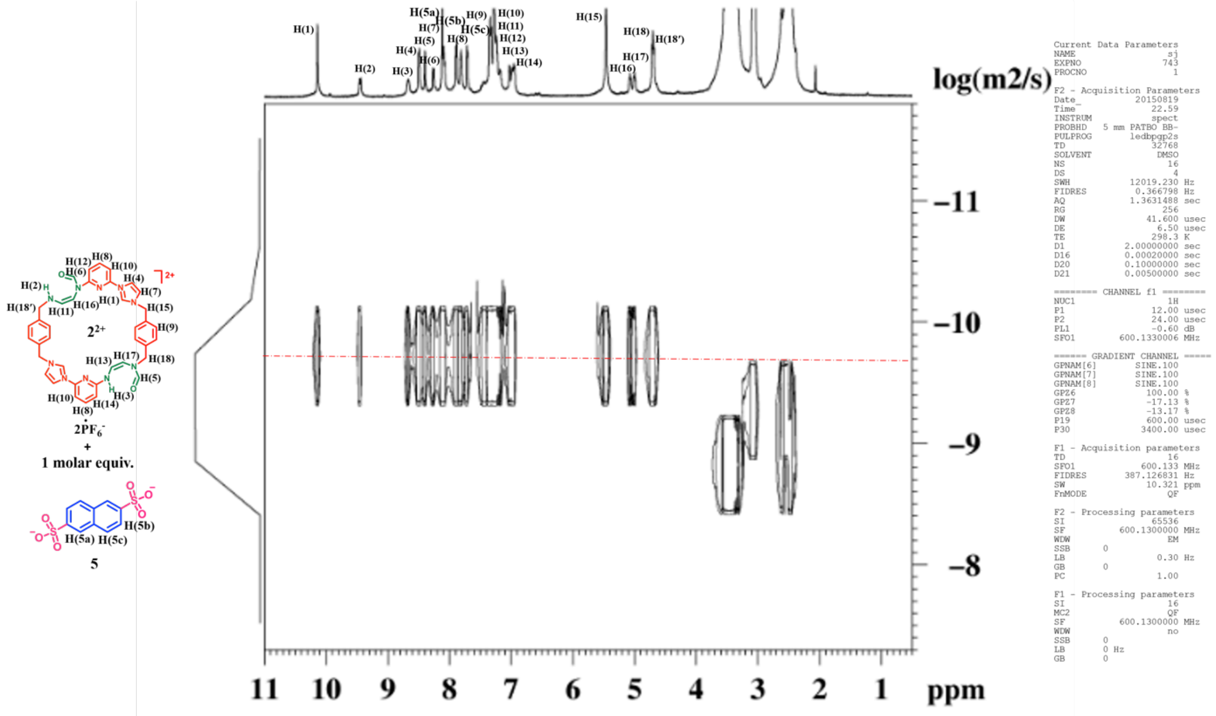

Figure S50. 600 MHz 2D-DOSY NMR spectra of $\mathbf{2}^{2+}{\cdot}2PF_6^-$ (2.00×10^{-2} M) recorded in the presence of 1 molar equiv. of 2,6-naphthalenedisulfonic acid and 2 molar equiv. of $TMA^+{\cdot}OH^-$ collected in DMSO-d_6 at 300K.

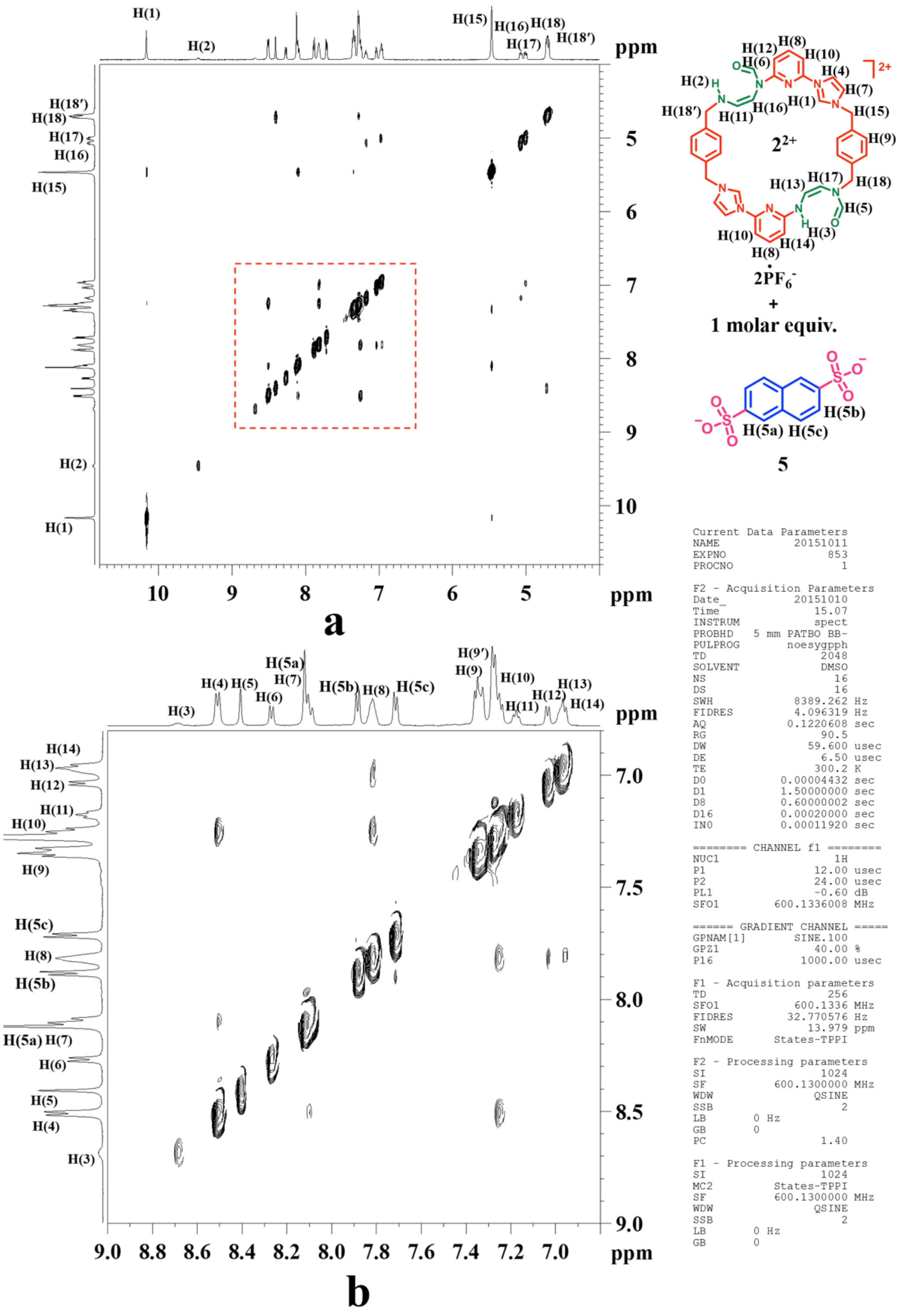

Figure S51. Full view (a) and expanded view (b) of the 600 MHz NOESY NMR spectrum of $\mathbf{2}^{2+}\cdot 2PF_6^-$ (2.00×10^{-2} M) recorded in the presence of 1 molar equiv. of 2,6-naphthalenedisulfonic acid and 2 molar equiv. of $TMA^+\cdot OH^-$ in DMSO-d_6 at 300K.

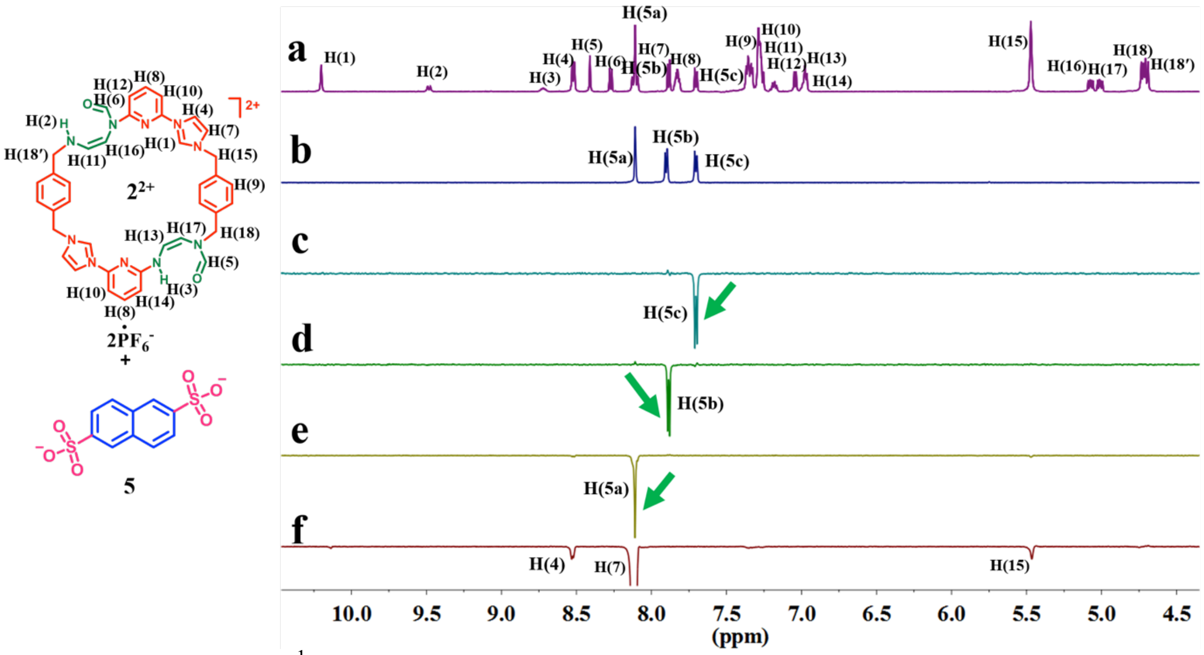

Figure S52. 600 MHz ^{1}H NMR and 1D NOE NMR spectra recorded in DMSO-d_6 at 300K: (a) ^{1}H NMR of $\mathbf{2}^{2+}\cdot 2PF_6^{-}$ in the presence of 1 molar equiv. of 2,6-naphthalenedisulfonic acid and 2 molar equiv. of $TMA^{+}\bullet OH^{-}$ ([host] = [guest] = 5.00×10^{-3} M), (b) ^{1}H NMR of the 2,6-naphthalenedisulfonic acid in the presence of 2 molar equiv. of $TMA^{+}\bullet OH^{-}$, and (c-e) 1D-NOE NMR spectra of $\mathbf{2}^{2+}\cdot 2PF_6^{-}$ in the presence of 1 molar equiv. of 2,6-naphthalenedisulfonic acid and 2 molar equiv. of $TMA^{+}\bullet OH^{-}$ ([host] = [guest] = 5.00×10^{-3} M). The green arrows indicate irradiation at the frequency of H(5a), H(5b) and H(5c) of 2,6-naphthalenedisulfonate (**5**). Shown in (f) is the 1D-NOE NMR spectra of $\mathbf{2}^{2+}\cdot 2PF_6^{-}$ (5.00×10^{-3} M). The green arrow indicates irradiation at the frequency of H(7) of $\mathbf{2}^{2+}\cdot 2PF_6^{-}$.

Solution binding studies and characterization of host-guest complex formed between 2^{2+} and dianionic guest 1,5-naphthalenedisulfonate (6):

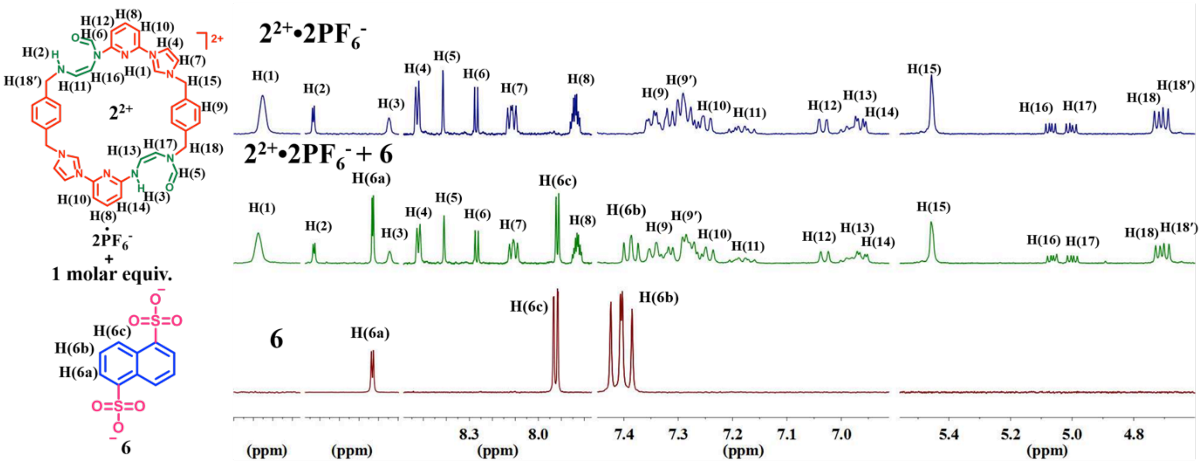

Figure S53. Expanded views of the ^{1}H NMR spectra of $\mathbf{2}^{2+}{\cdot}2PF_6^-$ (1.00×10^{-3} M) recorded in the absence and presence of 1 molar equiv. of **6** in DMSO-d_6 at 300 K over the indicated spectral ranges (600 MHz).

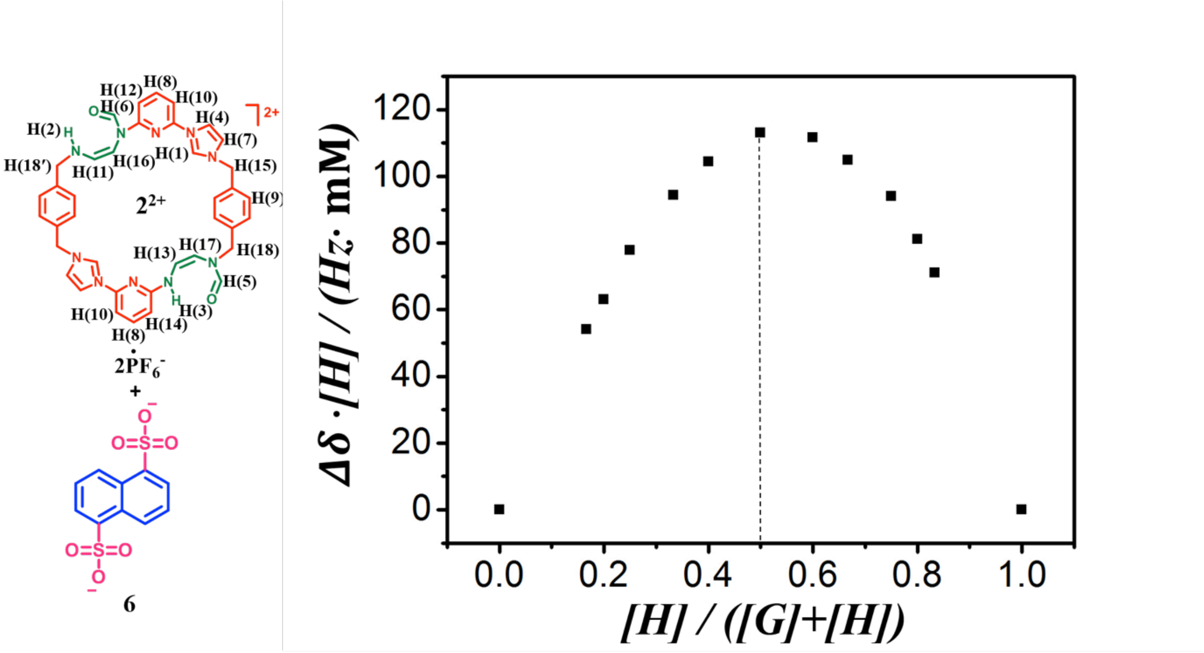

Figure S54. ^{1}H NMR Job-plots (600 MHz) corresponding to the binding between $\mathbf{2}^{2+}{\cdot}2PF_6^-$ and **6** ([host] + [guest] = 1.00×10^{-2} M). The maximum value was determined to be 0.5 at this concentration, a finding that is consistent with a 1:1 (host: guest) binding stoichiometry.[8]

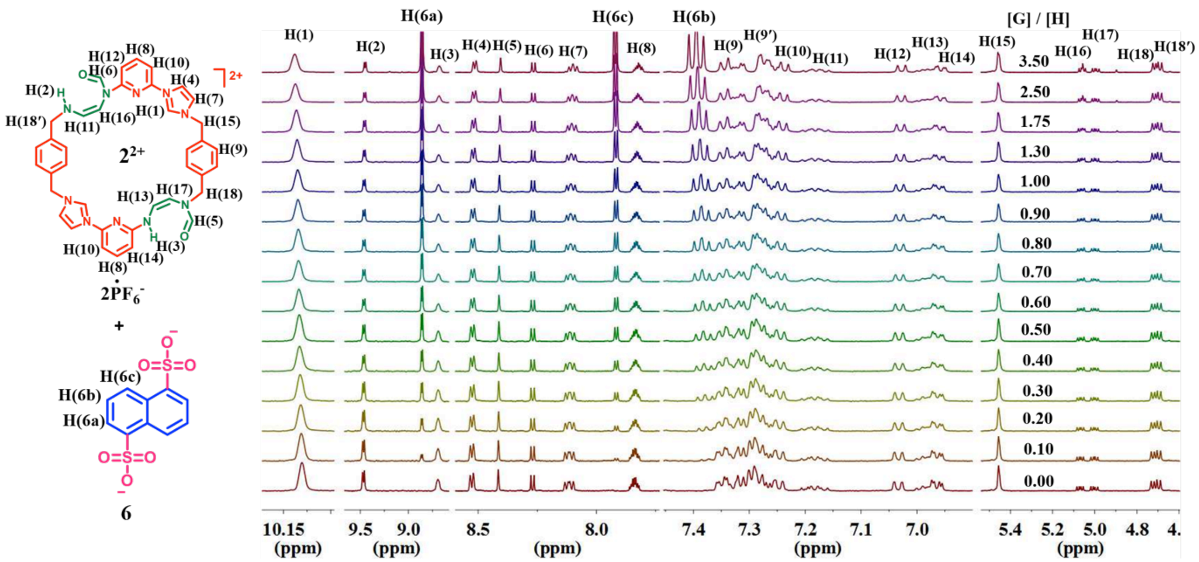

Figure S55. ^{1}H NMR spectroscopic titration of $\mathbf{2}^{2+}\cdot 2PF_6^-$ (1.00×10^{-3} M) with 1,5-naphthalenedisulfonic acid in the presence of 2 molar equiv. of $TMA^+\cdot OH^-$ collected in DMSO-d_6 at 300K (600 MHz).

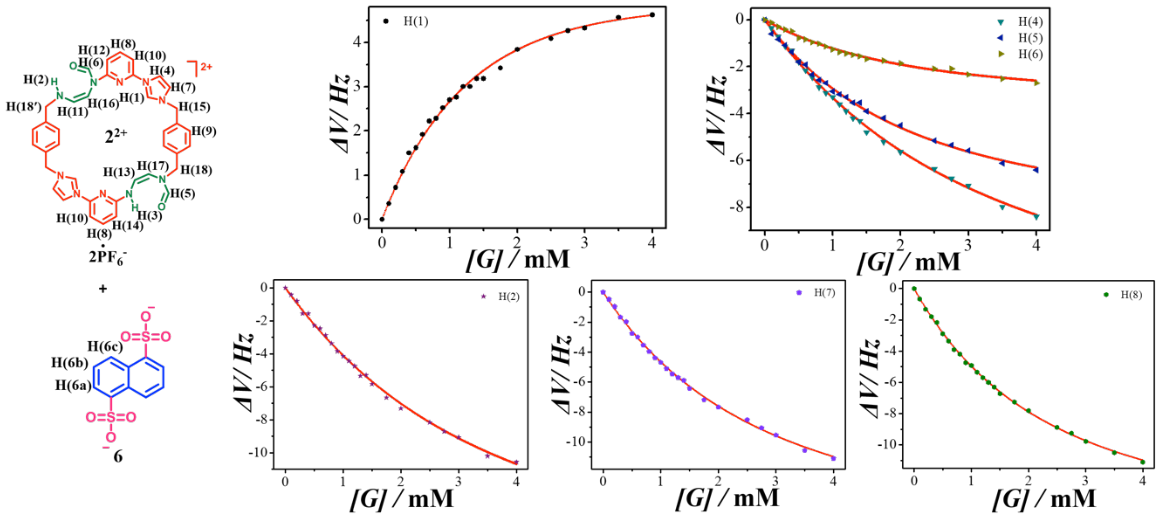

Figure S56. ^{1}H NMR binding isotherms corresponding to the interaction between $\mathbf{2}^{2+}\cdot 2PF_6^-$ and **6** in DMSO-d_6 at 300K. The chemical shift changes of H(1) and H(2), H(4), H(5), H(6), H(7) and H(8) on $\mathbf{2}^{2+}$ were used for the calculation of $(4.0 \pm 0.5) \times 10^2$ M^{-1} using the Hyperquad 2003 program.[9] The changes in chemical shift of the other protons present in $\mathbf{2}^{2+}$ and **6** overlapped and thus could not be accurately fit with either a Guassian or Lorentzian function. Also in some cases the chemical shift change was too small (less than 8.0 Hz); these signals were therefore not used in the calculation of the association constant.[10] The red dashed lines show the non-linear curve fit of the experimental data to the appropriate equation.

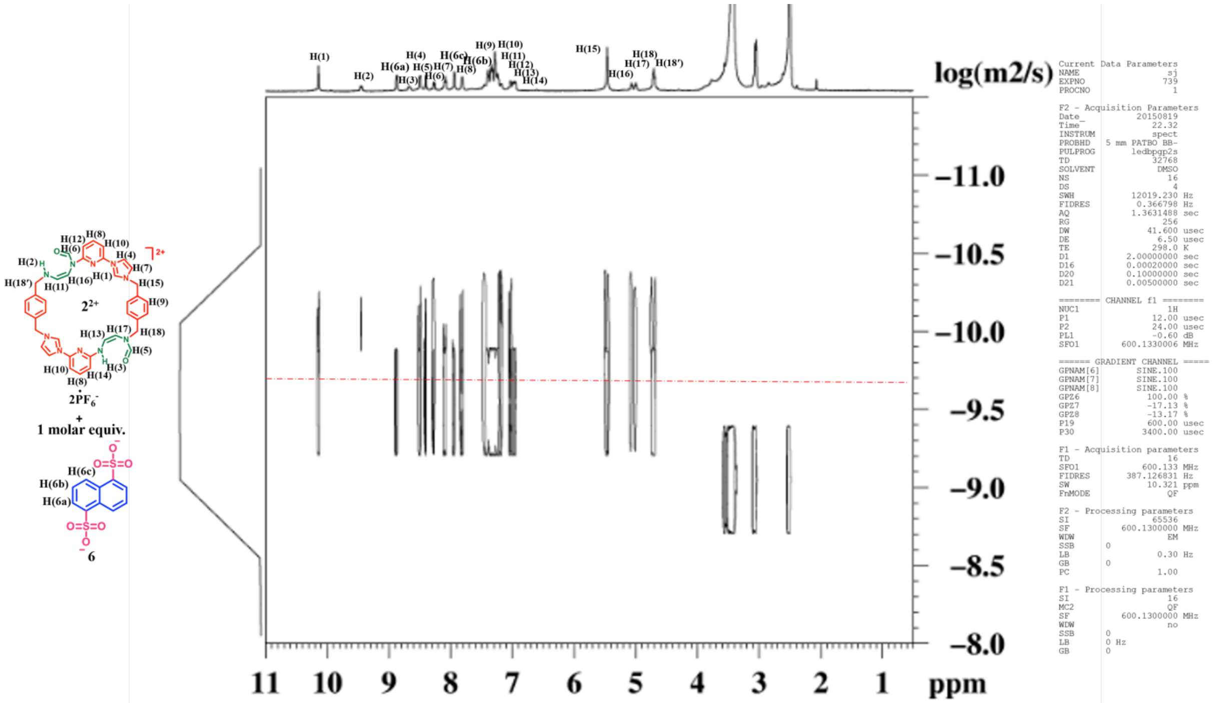

Figure S57. Expanded view of the 600 MHz 2D-DOSY NMR spectra of $\mathbf{2}^{2+}\cdot 2PF_6^-$ (2.00×10^{-2} M) recorded in the presence of 1 molar equiv. of 1,5-naphthalenedisulfonic acid and 2 molar equiv. of $TMA^+\cdot OH^-$ in DMSO-d_6 at 300K.

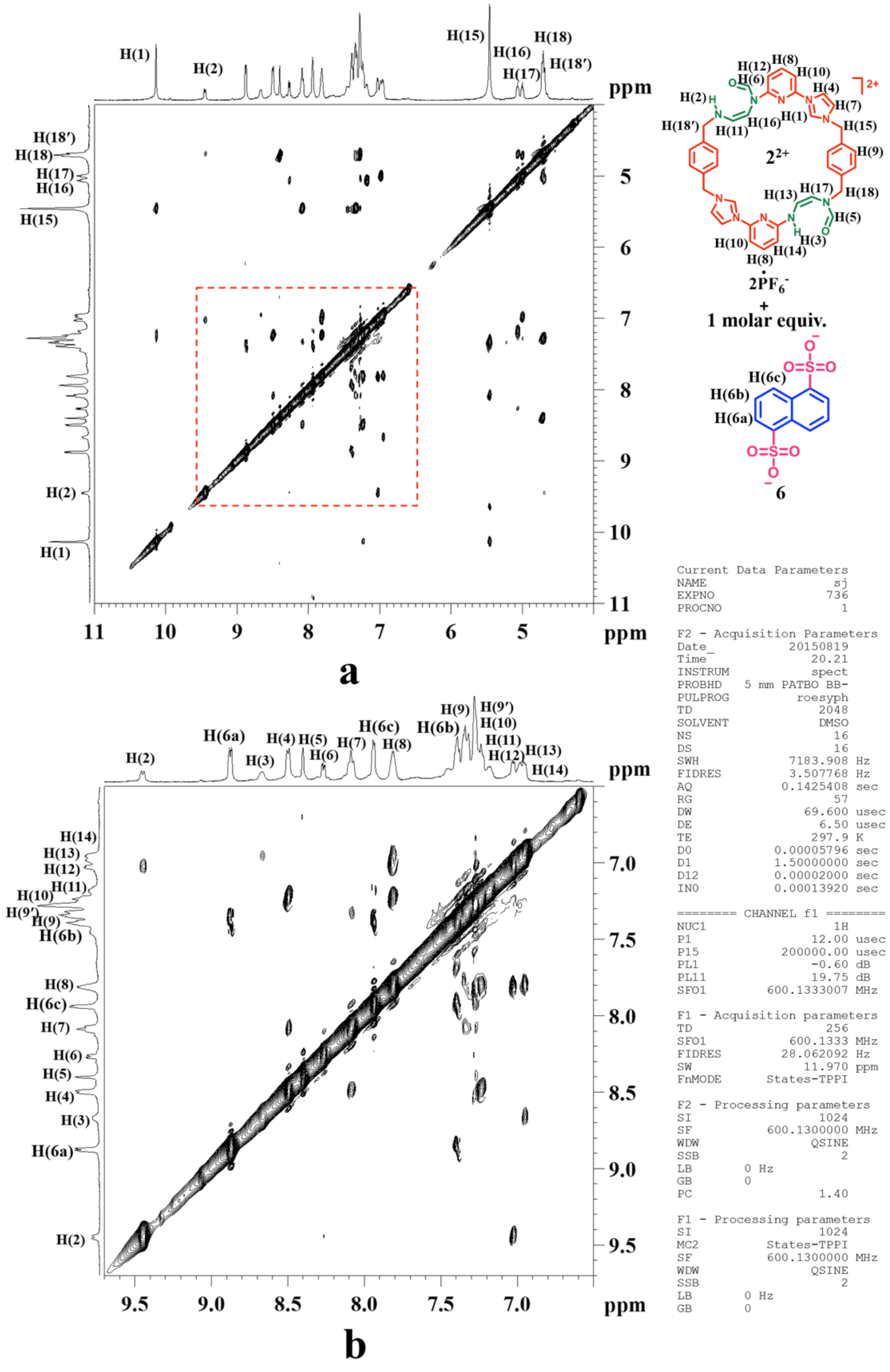

Figure S58. Full (a) and expanded view (b) of the 600 MHz ROESY NMR spectrum of $\mathbf{2}^{2+}\cdot 2PF_6^-$ (2.00×10^{-2} M) recorded in the presence of 1 molar equiv. of 1,5-naphthalenedisulfonic acid and 2 molar equiv. of $TMA^+\cdot OH^-$ in DMSO-d_6 at 300K.

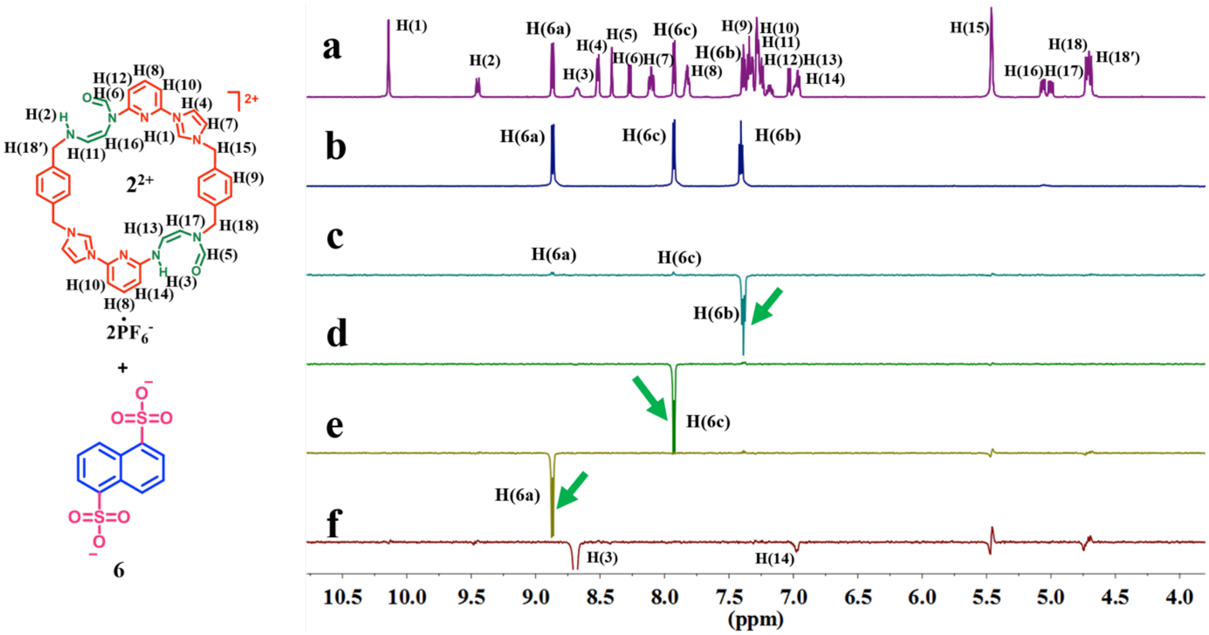

Figure S59. 600 MHz ^{1}H NMR and 1D NOE NMR spectra recorded in DMSO-d_6 at 300K: (a) ^{1}H NMR of $\mathbf{2}^{2+}{\cdot}2PF_6^-$ in the presence of 1 molar equiv. of 1,5-naphthalenedisulfonic acid and 2 molar equiv. of $TMA^+{\cdot}OH^-$ ([host] = [guest] = 5.00 × 10^{-3} M), (b) ^{1}H NMR of the 1,5-naphthalenedisulfonic acid in the presence of 2 molar equiv. of $TMA^+{\bullet}OH^-$, and (c-e) 1D NOE NMR spectra of $\mathbf{2}^{2+}{\cdot}2PF_6^-$ in the presence of 1 molar equiv. of 1,5-naphthalenedisulfonic acid and 2 molar equiv. of $TMA^+{\cdot}OH^-$ ([host] = [guest] = 5.00 × 10^{-3} M). The green arrows indicate irradiation at the frequency of H(6a), H(6b) and H(6c) of 1,5-naphthalenedisulfonate. Shown in (f) is the 1D NOE NMR spectrum of $\mathbf{2}^{2+}{\cdot}2PF_6^-$ (5.00 × 10^{-3} M). The green arrow indicates irradiation at the frequency of H(3) of $\mathbf{2}^{2+}{\cdot}2PF_6^-$.

Section S5: *Single crystal X-ray analysis of the complexes formed between* $\mathbf{2}^{2+}$ *and dianionic guest* ***5*** *or* ***6***

Table S3. Single crystal X-ray crystallographic data for [$\mathbf{2}^{2+}$·**5**·5H_2O] and [$\mathbf{2}^{2+}$·**6**·2.5H_2O].

	$\mathbf{2}^{2+}$·**5**·5H_2O	$\mathbf{2}^{2+}$·**6**·2.5H_2O
CCDC No.	1433723	1433722
description	prism	needle
colour	colourless	colourless
From solution	DMF/water /acetonitrile	DMF/water /acetonitrile
empirical formula	C_{48} H_{52} N_{10} O_{13} S_2	C_{48} H_{47} N_{10} $O_{10.50}$ S_2
Mr	1041.12	996.08
crystal size (mm^3)	0.05 × 0.03 × 0.02	0.11 × 0.04 × 0.03
crystal system	Monoclinic	Triclinic
space group	P2(1)/n	P-1
a [Å]	13.490(3)	16.853(3)
b [Å]	18.126(4)	17.211(3)
c [Å]	20.197(4)	18.087(4)
α [deg]	90.00	99.16(3)
β [deg]	101.26(3)	91.70(3)
γ[deg]	90.00	96.78(3)
V/ [Å^3]	4843.4(17)	5136.5(18)
d/[g/cm^3]	1.428	1.288
Z	4	4
T [K]	173(2)	173(2)
R1, wR2 $I > 2ó(I)$	0.1162 0.2200	0.1539 0.3146
R1, wR2 (all data)	0.1480 0.2401	0.2232 0.3478
quality of fit	1.005	0.997

Single-crystal X-ray structure of [$\mathbf{2}^{2+}$·5**·5H_2O]:**

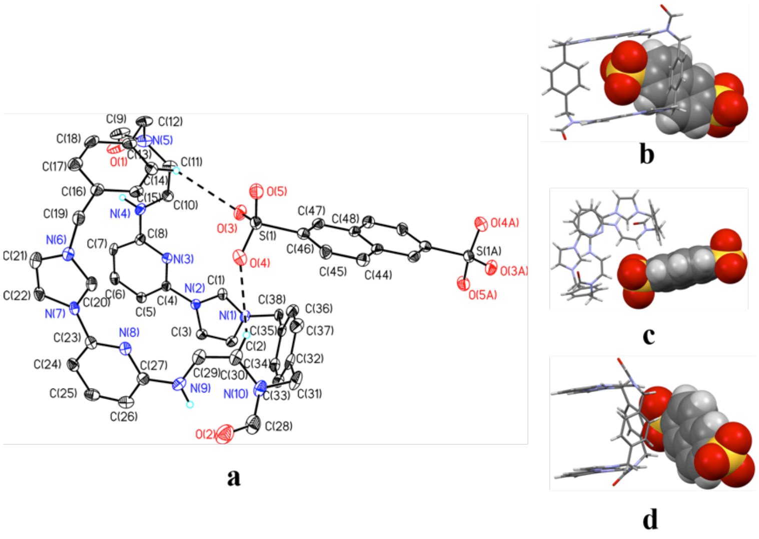

Figure S60. Structure of $\mathbf{2}^{2+}$ viewed from the top view in ellipsoid form (a), as well as top (b), side (c) and front views (d) of $\mathbf{2}^{2+}$ in stick form. In these structures, $\mathbf{2}^{2+}$ adopts a "clip-like" conformation with a molecule of **5** bound to its outer periphery in the final structure [$\mathbf{2}^{2+}$·**5**·5H_2O]. Displacement ellipsoids are scaled to the 25% probability level. Some or all of the counter anions, solvent molecules and hydrogen atoms have been omitted for clarity. The symmetry transformation invoked by the additional "A" letter in the atom labels on the **5** is (1-x, 1-y, 1-z). Possible intermolecular hydrogen bonds: selected interatomic distances [Å]: O(4)…C(30) 3.501(8), O(3)…C(14) 3.627(7); selected bond angles: C(30)-H(30A)…O(4) 139.5º, C(14)-H(14A)…O(3) 148.8º.

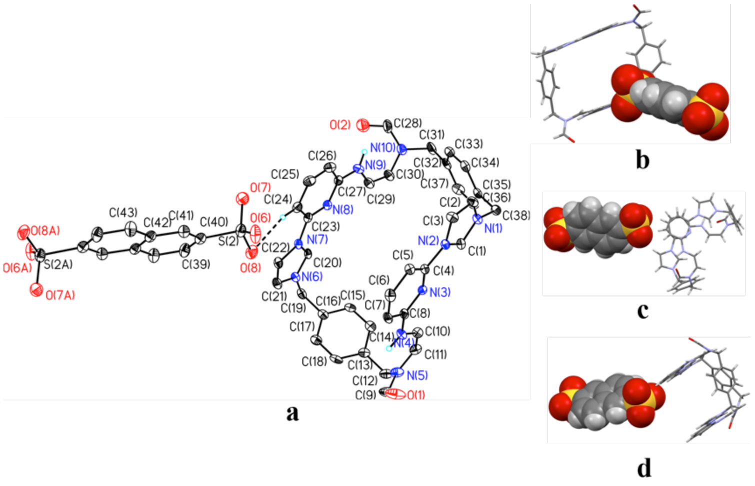

Figure S61. Structure of $\mathbf{2}^{2+}$ viewed from the top in ellipsoid form (a), as well as top (b), side (c) and front views (d) of $\mathbf{2}^{2+}$ in stick form. In these structures, $\mathbf{2}^{2+}$ adopts a "clip-like" conformation with a molecule of **5** bound to its outer periphery in the final structure [$\mathbf{2}^{2+}$•**5**•5H_2O]. Displacement ellipsoids are scaled to the 25% probability level. Some or all of the counter anions, solvent molecules and hydrogen atoms have been omitted for clarity. The symmetry transformation invoked by the additional "A" letter in the atom labels on the **5** is (1-x, 1-y, 1-z). Possible intermolecular hydrogen bonds: selected interatomic distances [Å]: O(8)…C(24) 3.399(7); selected bond angles: C(24)-H(24A)…O(8) 167.2º.

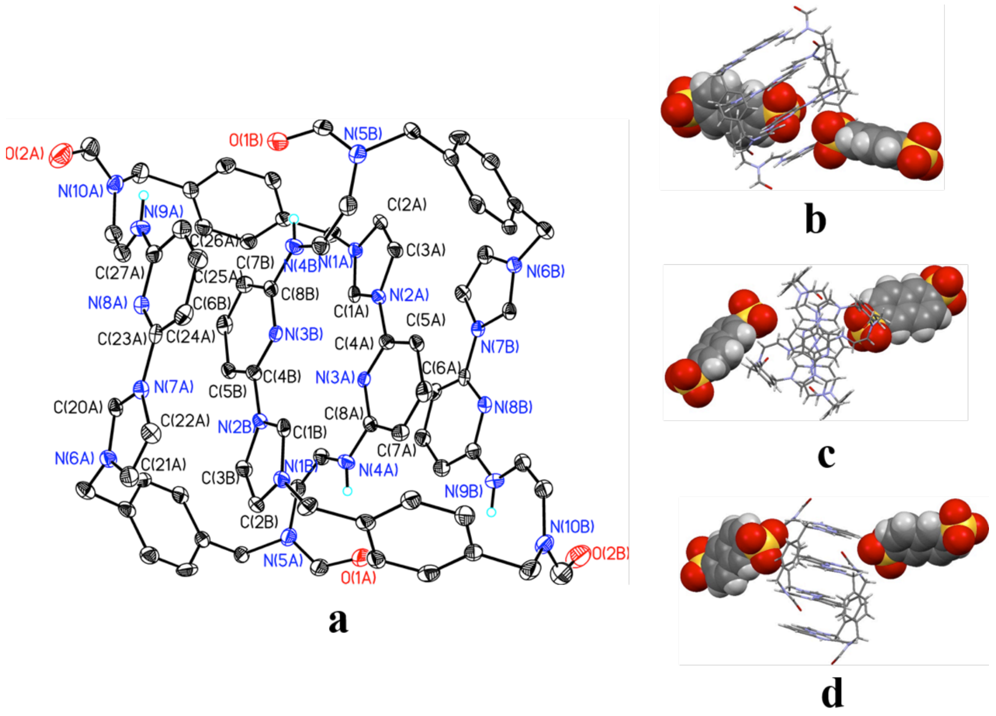

Figure S62. Structure of $\mathbf{2}^{2+}$ viewed from the top (a) in ellipsoid form, as well as top (b), side (c), and front views (d) in stick form. In these structures, $\mathbf{2}^{2+}$ is organized in a head-to-tail racemic dimer which is present in the final structure [$\mathbf{2}^{2+}$•**5**•5H_2O]. Displacement ellipsoids are scaled to the 25% probability level. Some or all of the counter anions, solvent molecules and hydrogen atoms have been omitted for clarity. The symmetry transformation invoked by the additional "A" letter and "B" letter in the atom labels on the macrocycle $\mathbf{2}^{2+}$ are (x, y, z) and (-x, -y, 1-z). Possible intramolecular π…π interaction: Selected interatomic distances [Å]: N(3B)…C(24A) 3.594(8), N(3B)…C(23A) 3.442(7), C(8B)…C(26A) 3.498(9), C(8B)…C(27A) 3.341(8), C(6B)…N(8A) 3.726(8), N(3B)…N(3A) 3.366(6), C(8A)…N(3B) 3.779(8), N(3B)…C(4A) 3.306(7), C(4B)…C(8A) 3.510(8), C(5B)…C(23A) 4.020(8), C(4B)…C(23A) 3.495(8), N(7A)…N(2B) 3.409(6), C(21A)…C(2B) 3.448(9), C(21A)…C(3B) 3.550(1), N(6A)…C(3B) 3.590(9).

Single-crystal analysis for structure [$\mathbf{2}^{2+}$·**6**·2.5H_2O]**:**

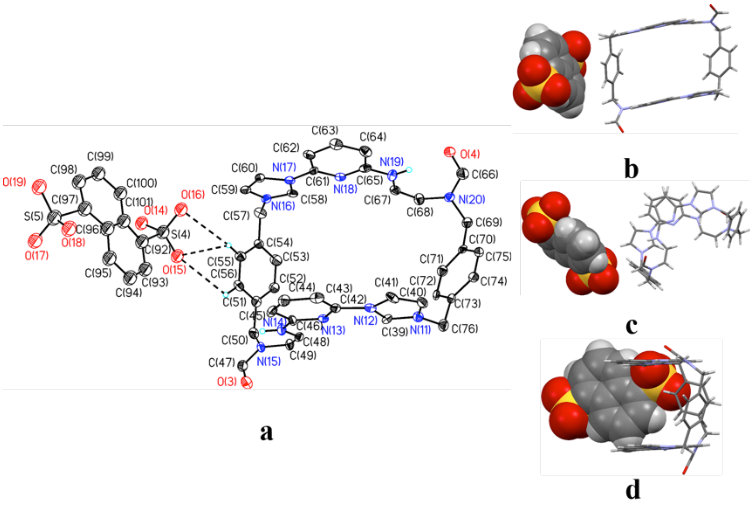

Figure S63. Structure of $\mathbf{2}^{2+}$ viewed from the top (a) in ellipsoid form, as well as top (b), side (c), and front views (d) in stick form. In these structures, $\mathbf{2}^{2+}$ adopts a "clip" conformation and **6** is bound to the outer periphery of the final structure [$\mathbf{2}^{2+}$•**6**•2.5H_2O]. Displacement ellipsoids are scaled to the 25% probability level. Some or all of the counter anions, solvent molecules and hydrogen atoms have been omitted for clarity. Selected bond distances between $\mathbf{2}^{2+}$ and the anion **6** are as follows. Intermolecular hydrogen bonds: selected interatomic distances [Å]: O(15)…C(55) 3.23(2), O(15)…C(56) 3.48(2), O(16)…C(55) 3.59(2). Selected bond angles: C(55)-H(55A)…O(15) 95.9º, C(56)-H(56A)…O(15) 85.8º, C(55)-H(55A)…O(16) 132.7º.

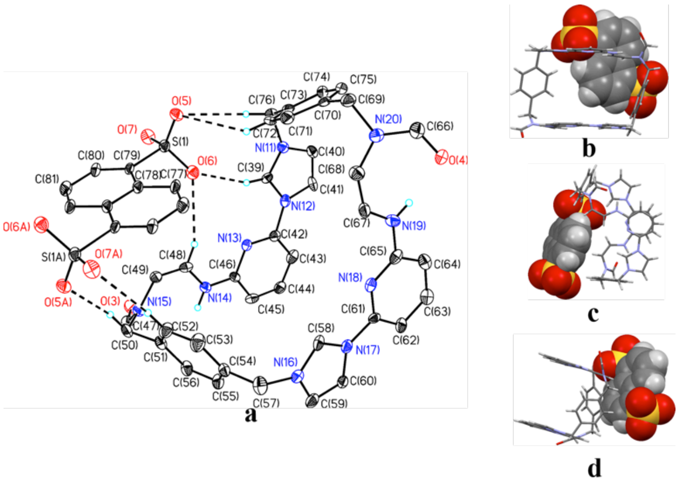

Figure S64. Structure of $\mathbf{2}^{2+}$ viewed from the top (a) in ellipsoid form, as well as top (b), side (c), and front views (d) shown in stick form. In these structures, $\mathbf{2}^{2+}$ adopts a "clip" conformation and **6** is bound to the outer periphery of the final structure [$\mathbf{2}^{2+}$•**6**•2.5H_2O]. Displacement ellipsoids are scaled to the 25% probability level. Some or all of the counter anions, solvent molecules and hydrogen atoms have been omitted for clarity. The symmetry transformation invoked by the additional "A" letter in the atom labels on the **6** is (1-x, 1-y, -z). Intermolecular hydrogen bonds: selected interatomic distances [Å]:

O(5A)…C(50) 3.53(1), O(5)…C(76) 3.77(1), O(5)…C(72) 3.59(1), O(6)…C(39) 2.97(1), O(6)…C(48) 3.57(1), O(7A)…C(52) 3.94(1). Selected bond angles: C(50)-H(50B)…O(5A) 163.5º, C(72)-H(72A)…O(5) 140.9º, C(76)-H(76A)…O(5) 156.3º, C(48)-H(48A)…O(6) 154.2º, C(39)-H(39A)…O(6) 152.7º, C(52)-H(52A)…O(7A) 159.0º.

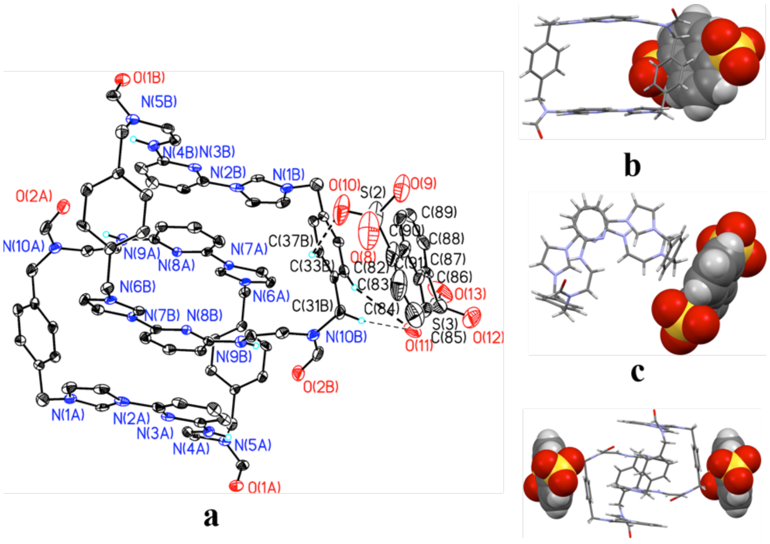

d

Figure S65. Structure of **2**$^{2+}$ viewed from the top (a) in ellipsoid form, as well as top (b), side (c), and front views (d) shown in stick form. In these structures, **2**$^{2+}$ adopts a "clip" conformation and **6** is bound to the outer periphery of the final structure [**2**$^{2+}$·**6**·2.5H_2O]. Displacement ellipsoids are scaled to the 25% probability level. Some or all of the counter anions, solvent molecules and hydrogen atoms have been omitted for clarity. The symmetry transformation invoked by the additional "A" letter and "B" letter in the atom labels on the **2**$^{2+}$ are (x, y, z) and (1-x, 2-y, 1-z). Intermolecular hydrogen bonds: selected interatomic distances [Å]: O(11)…C(31B) 3.46(1), O(11)…C(33B) 3.68(2), O(10)…C(37B) 3.23(2); selected bond angles: C(31B)-H(31BA)…O(11) 164.5º, C(33B)-H(33BA)…O(11) 130.6º, C(37B)-H(37BA)…O(10) 115.9º. Possible intermolecular π…π donor-acceptor interactions: selected interatomic distances [Å]: C(90)…C(37B) 3.46(2), C(91)…C(37B) 3.51(2), C(86)…C(33B) 3.86(2), C(87)…C(33B) 3.51(2), C(88)…C(33B) 3.44(2).

Section S6: *Mass spectrometric analysis of the complexes formed between* $\mathbf{2}^{2+}$ *and dianionic guest* ***4***, ***5*** *or* ***6***

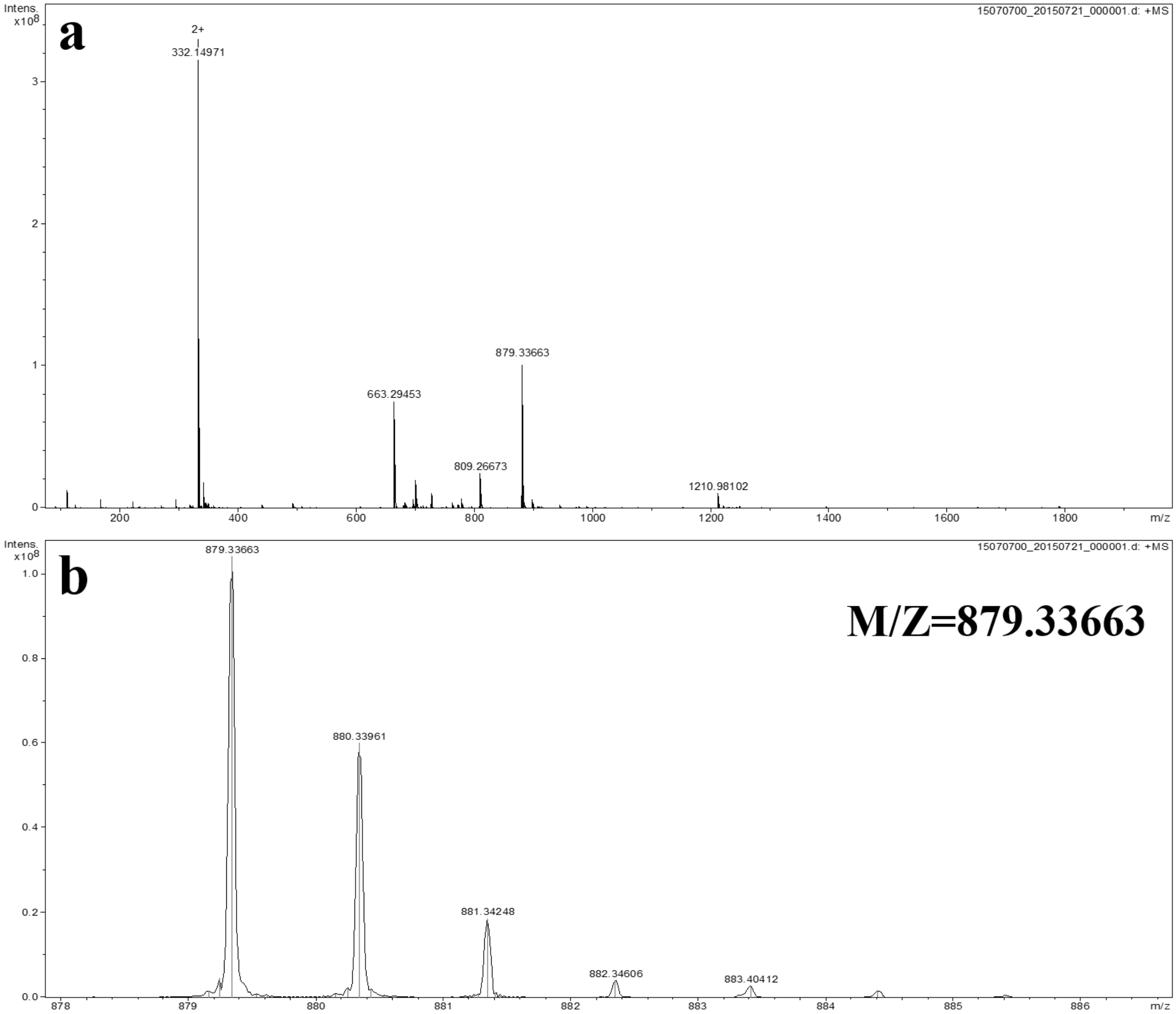

Figure S66. Full (a) and expanded view (b) of the ESI high resolution mass spectrum obtained from a mixture of $\mathbf{2}^{2+}$ and guest **4**.

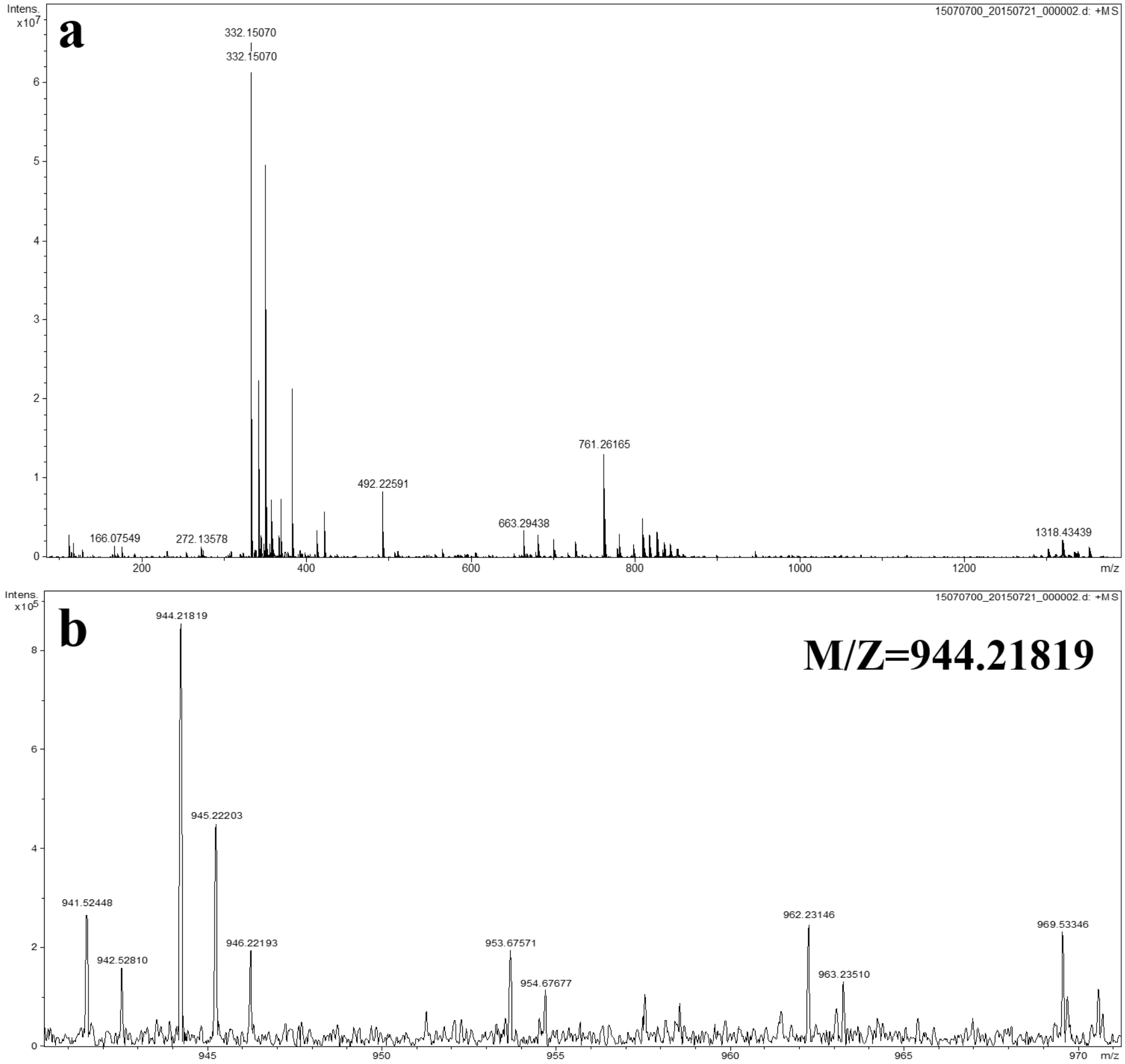

Figure S67. Full (a) and expanded view (b) of the ESI high resolution mass spectrum obtained from a mixture of $\mathbf{2}^{2+}$ and guest **5**.

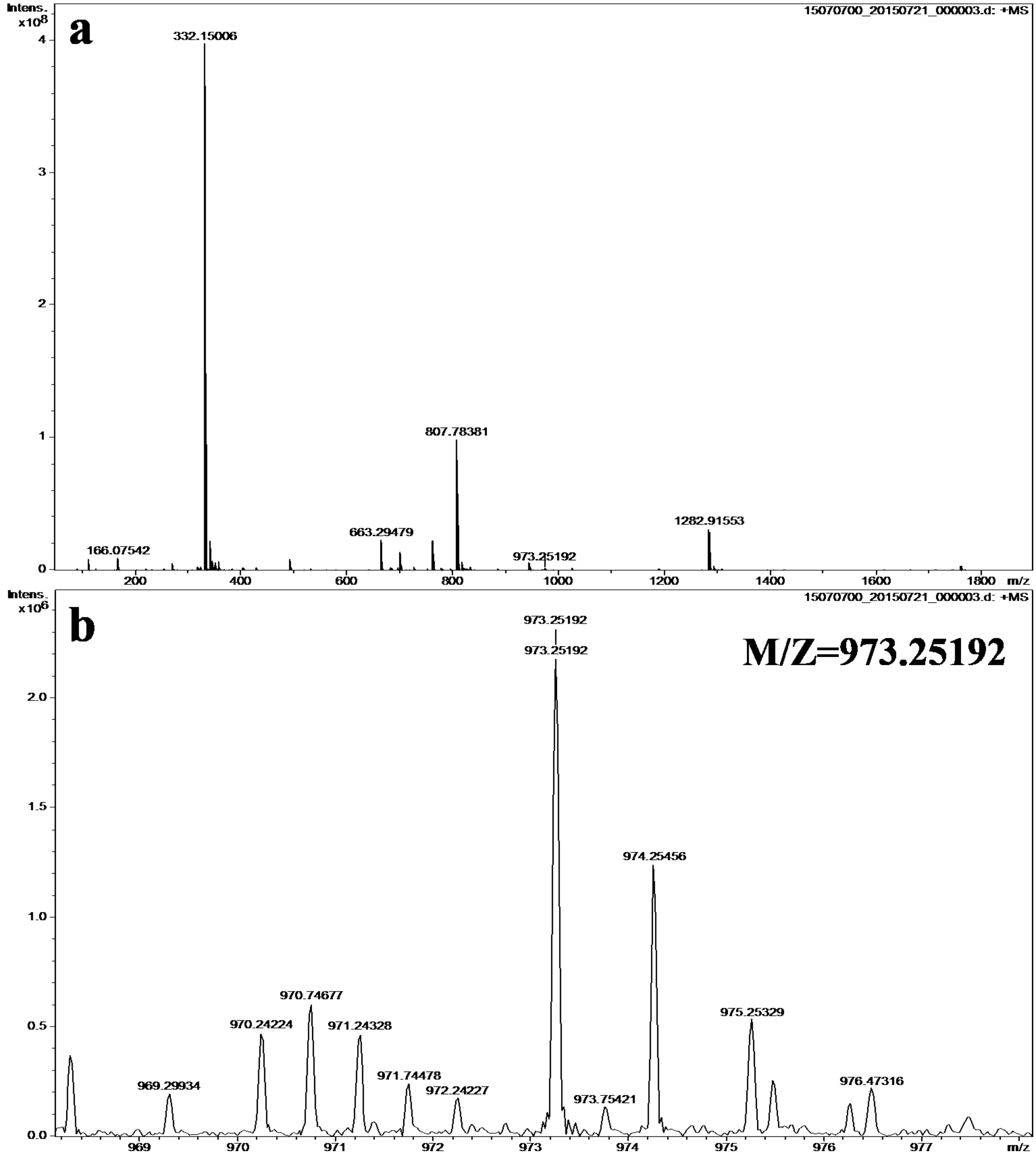

Figure S68. Full (a) and expanded view (b) of the ESI high resolution mass spectrum obtained from a mixture of $\mathbf{2}^{2+}$ and guest **6**.

Table S4. Summary of ESI-MS results.

**Presumed Complex**	**Peak Assignment**	**Calculated m/z**	**Observed m/z**
[$\mathbf{2}^{2+}$•**4**]	[$\mathbf{2}^{2+}$+**4**+H]$^{+•}$	879.3367	879.3366
[$\mathbf{2}^{2+}$•**5**]	[$\mathbf{2}^{2+}$+**5**-6H]$^{+•}$	944.2159	944.2182
[$\mathbf{2}^{2+}$•**6**]	[$\mathbf{2}^{2+}$+**6**+Na]$^{+•}$	973.2526	973.2519

Section S7: *Solution binding studies and characterization of the OH⁻ ring-opening process leading to the irreversible disassembly of the interpenetrated structures [$\mathbf{1}^{4+}$·**4**] and [$\mathbf{1}^{4+}$·**5**]*

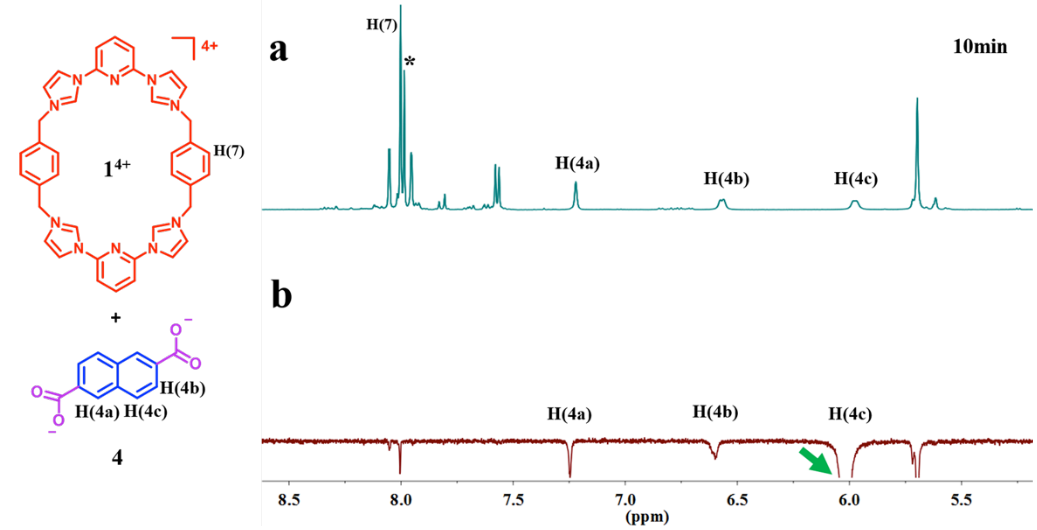

Figure S69. 600 MHz ^{1}H NMR and 1D NOE NMR spectra recorded in DMF-d_7 /D_2O/ND_3·D_2O (1/0.98/0.02, v/v/v) at 298K: (a) ^{1}H NMR spectrum of $\mathbf{1}^{4+}$·4PF_6^- recorded in the presence of 1 molar equiv. of 2,6-naphthalenedicarboxylic acid and 2 molar equiv. of TMA^+·OH^- at 10 min (reaction time) ([host] = [guest] = 5.00 × 10^{-3} M), (b) 1D NOE NMR spectra of $\mathbf{1}^{4+}$·4PF_6^- in the presence of 1 molar equiv. of 2,6-naphthalenedicarboxylic acid and 2 molar equiv. of TMA^+·OH^- at 10 min (reaction time) ([host] = [guest] = 5.00 × 10^{-3} M). There is one proton signal of the solvent DMF-d_7 that is labeled with a "*". The green arrow indicates irradiation at the frequency of H(4c) of 2,6-naphthalenedicarboxylate (**4**).

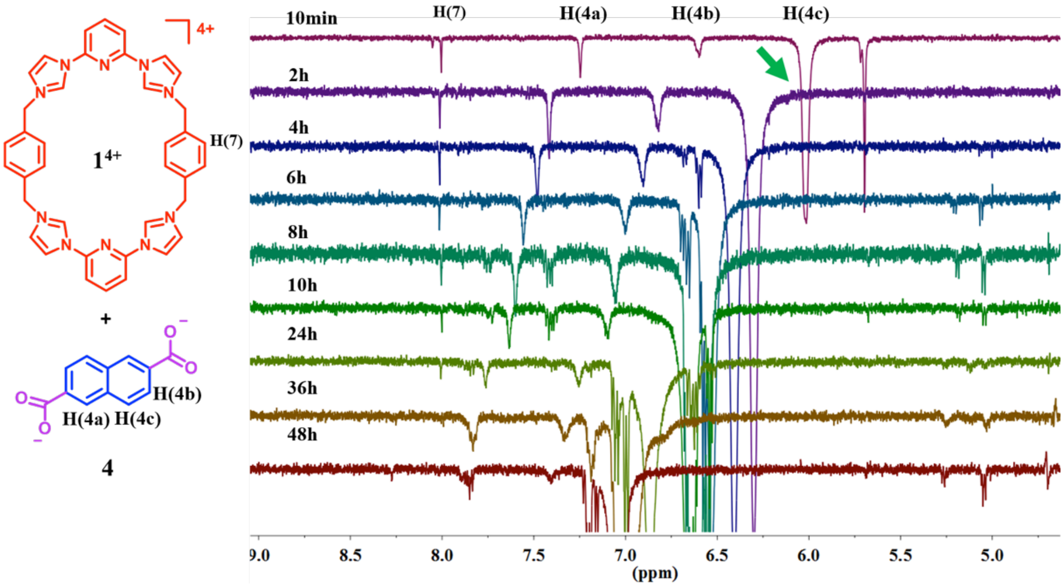

Figure S70. 1D NOE NMR spectra of $\mathbf{1}^{4+}$•4PF_6^- recorded in the presence of 1 molar equiv. of 2,6-naphthalenedicarboxylic acid and 2 molar equiv. of TMA^+·OH^- ([host] = [guest] = 5.00 × 10^{-3} M) in DMF-d_7 /D_2O/ND_3·D_2O (1/0.98/0.02, v/v/v) at 298K (600 MHz). The green arrows indicate irradiation at the frequency of H(4c) of 2,6-naphthalenedicarboxylate (**4**). These findings are consistent with **4** dethreading from the pseudorotaxane structure [$\mathbf{1}^{4+}$·**4**] as reaction proceeds.

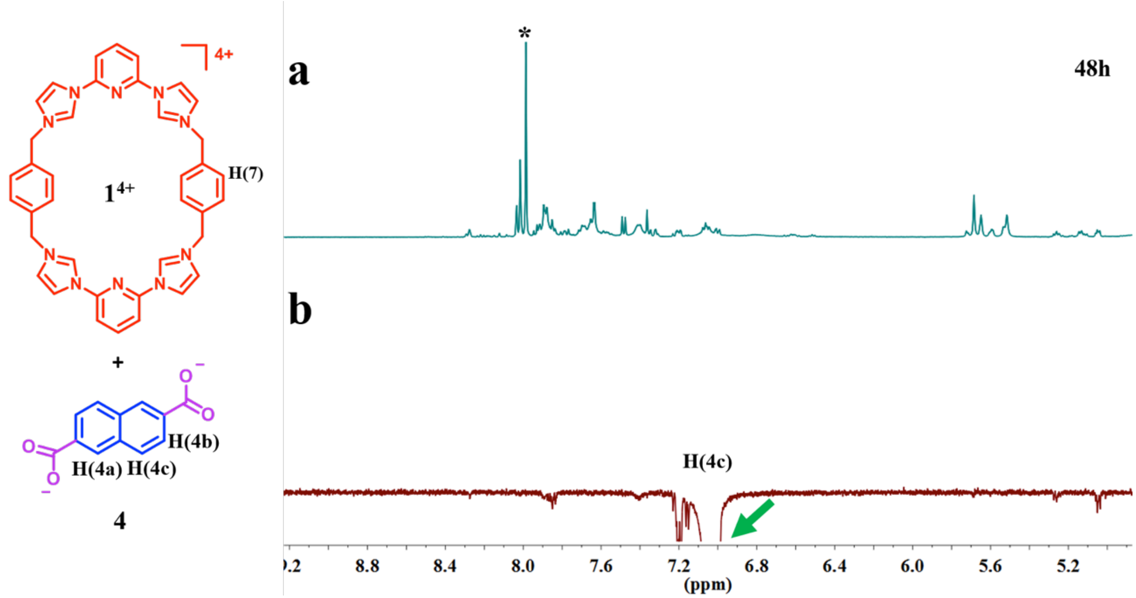

Figure S71. 600 MHz ^{1}H NMR and 1D NOE NMR spectra recorded in DMF-d_7 /D_2O/ND_3·D_2O (1/0.98/0.02, v/v/v) at 298K: (a) ^{1}H NMR of $\mathbf{1}^{4+}$·$4PF_6^-$ in the presence of 1 molar equiv. of 2,6-naphthalenedicarboxylic acid and 2 molar equiv. of TMA^+·OH^- at 48 h (reaction time) ([host] = [guest] = 5.00 × 10^{-3} M), (b) 1D NOE NMR spectra of $\mathbf{1}^{4+}$·$4PF_6^-$ recorded in the presence of 1 molar equiv. of 2,6-naphthalenedicarboxylic acid and 2 molar equiv. of TMA^+•OH^- at 48 h (reaction time) ([host] = [guest] = 5.00 × 10^{-3} M), The one proton signal of the solvent DMF-d_7 is labeled with a "*". The green arrow indicates irradiation at the frequency of H(4c) of 2,6-naphthalenedicarboxylate (**4**).

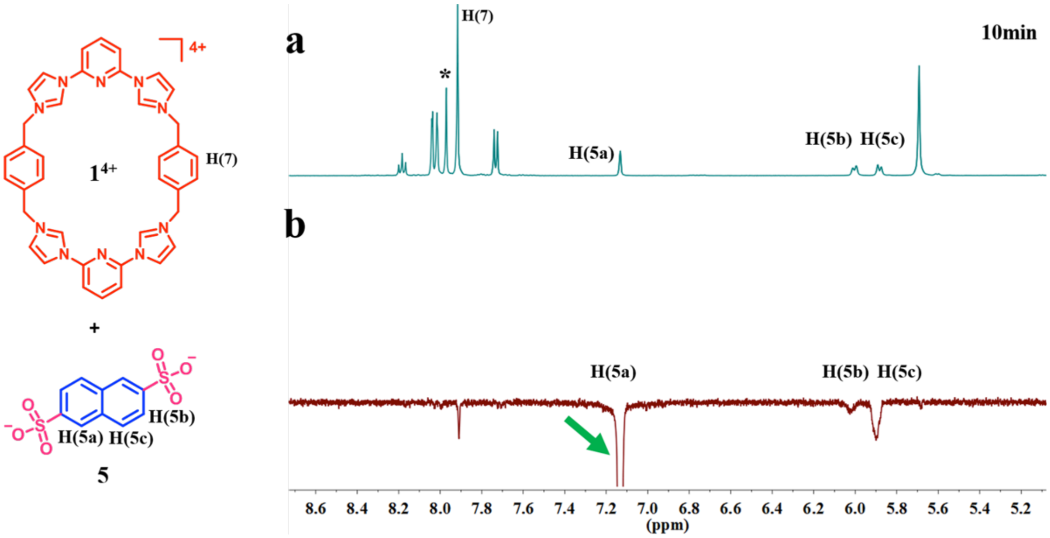

Figure S72. 600 MHz ^{1}H NMR and 1D NOE NMR spectra recorded in DMF-d_7 /D_2O/ND_3·D_2O (1/0.98/0.02, v/v/v) at 298K: (a) ^{1}H NMR of $\mathbf{1}^{4+}$·$4PF_6^-$ in the presence of 1 molar equiv. of 2,6-naphthalenedisulfonic acid and 2 molar equiv. of TMA^+·OH^- at 10min (reaction time) ([host] = [guest] = 5.00 × 10^{-3} M), (b) 1D NOE NMR spectra of $\mathbf{1}^{4+}$·$4PF_6^-$ in the presence of 1 molar equiv. of 2,6-naphthalenedisulfonic acid and 2 molar equiv. of TMA^+·OH^- at 10min (reaction time) ([host] = [guest] = 5.00 × 10^{-3} M). The one proton signal of the solvent DMF-d_7 is labeled with a "*". The green arrows indicate irradiation at the frequency of H(5a) of 2,6-naphthalenedisulfonate (**5**).

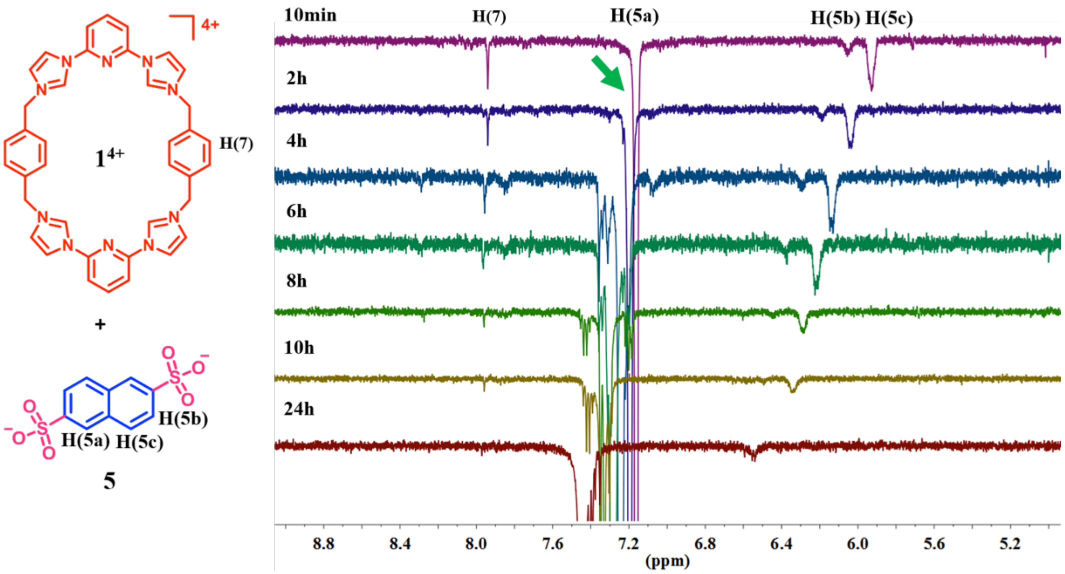

Figure S73. 1D NOE NMR spectra of $\mathbf{1}^{4+}\cdot 4PF_6^-$ recorded in the presence of 1 molar equiv. of 2,6-naphthalenedisulfonic acid and 2 molar equiv. of $TMA^+\cdot OH^-$ ([host] = [guest] = 5.00×10^{-3} M) in DMF-d_7 /D_2O/$ND_3\cdot D_2O$ (1/0.98/0.02, v/v/v) at 298K (600 MHz). The green arrows indicate irradiation at the frequency of H(5a) of 2,6-naphthalenedisulfonate (**5**). These findings are consistent with **5** dethreading from the pseudorotaxane structure [$\mathbf{1}^{4+}\cdot\mathbf{5}$] as the reaction proceeds.

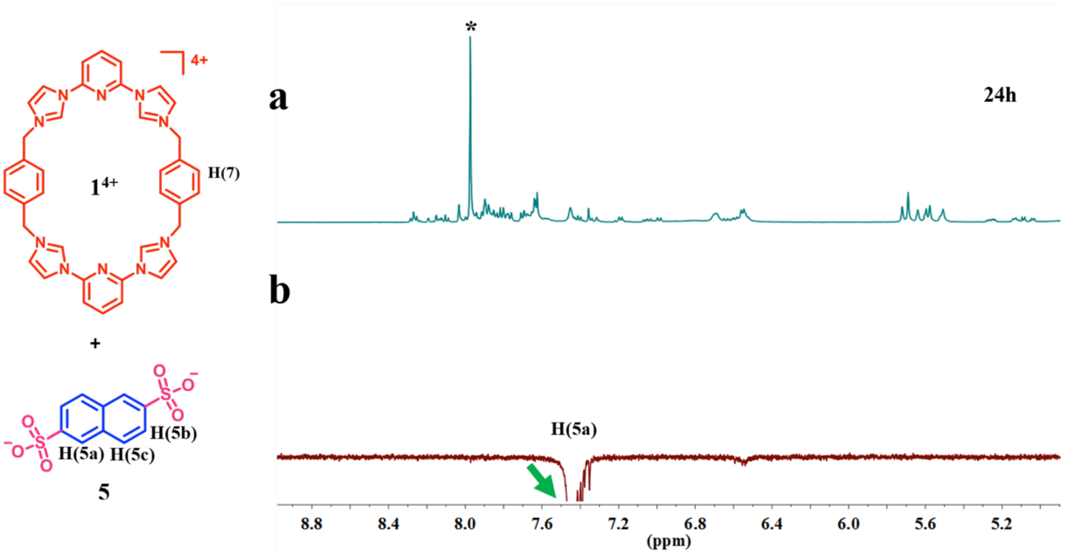

Figure S74. 600 MHz ^{1}H NMR and 1D NOE NMR spectra recorded in DMF-d_7 /D_2O/$ND_3\cdot D_2O$ (1/0.98/0.02, v/v/v) at 298K: (a) ^{1}H NMR spectra of $\mathbf{1}^{4+}\cdot 4PF_6^-$ recorded in the presence of 1 molar equiv. of 2,6-naphthalenedisulfonic acid and 2 molar equiv. of $TMA^+\cdot OH^-$ at 24 h (reaction time) ([host] = [guest] = 5.00×10^{-3} M), (b) 1D NOE NMR spectra of $\mathbf{1}^{4+}\bullet 4PF_6^-$ in the presence of 1 molar equiv. of 2,6-naphthalenedisulfonic acid and 2 molar equiv. of $TMA^+\cdot OH^-$ at 24 h (reaction time) ([host] = [guest] = 5.00×10^{-3} M). The one proton signal of the solvent DMF-d_7 is labeled with a “*”. The green arrows indicate irradiation at the frequency of H(5a) of 2,6-naphthalenedisulfonate (**5**).

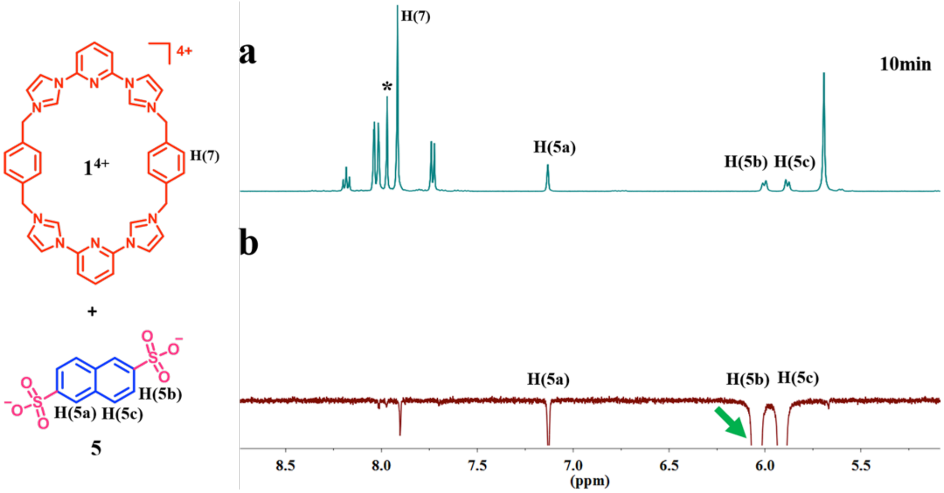

Figure S75. 600 MHz ^{1}H NMR and 1D NOE NMR spectra recorded in DMF-d_7 /D_2O/ND_3·D_2O (1/0.98/0.02, v/v/v) at 298K: (a) ^{1}H NMR of **1**$^{4+}$·$4PF_6^-$ in the presence of 1 molar equiv. of 2,6-naphthalenedisulfonic acid and 2 molar equiv. of TMA^+·OH^- at 10min (reaction time) ([host] = [guest] = 5.00 × 10^{-3} M), (b) 1D NOE NMR spectra of **1**$^{4+}$·$4PF_6^-$ in the presence of 1 molar equiv. of 2,6-naphthalenedisulfonic acid and 2 molar equiv. of TMA^+·OH^- at 10min (reaction time) ([host] = [guest] = 5.00 × 10^{-3} M). The one proton signal of the solvent DMF-d_7 is labeled with a "*". The green arrow indicates irradiation at the frequency of H(5b) of 2,6-naphthalenedisulfonate (**5**).

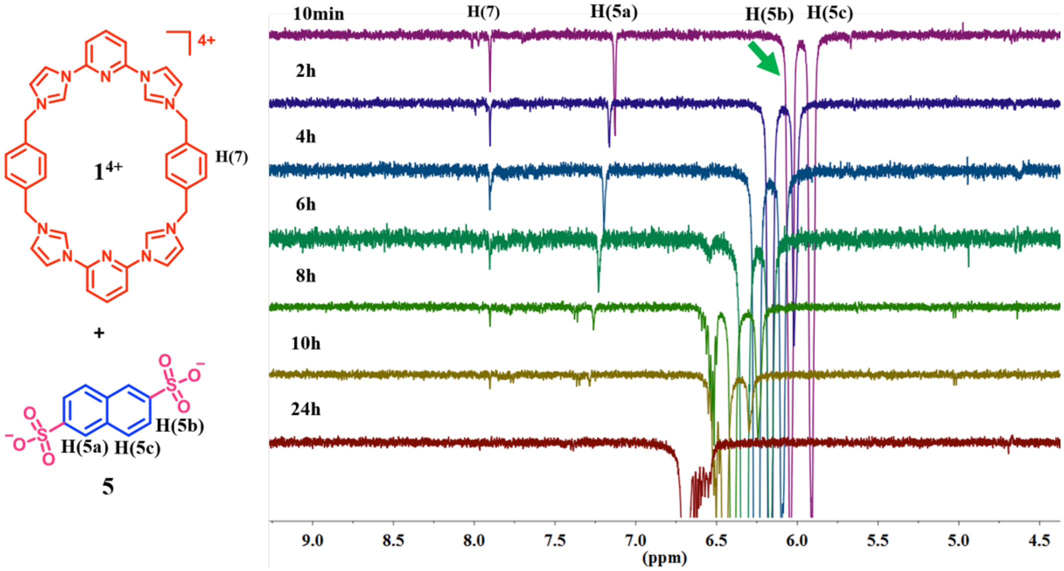

Figure S76. 1D NOE NMR spectra of **1**$^{4+}$·$4PF_6^-$ recorded in the presence of 1 molar equiv. of 2,6-naphthalenedisulfonic acid and 2 molar equiv. of TMA^+·OH^- ([host] = [guest] = 5.00 × 10^{-3} M) in DMF-d_7 /D_2O/ND_3·D_2O (1/0.98/0.02, v/v/v) at 298K (600 MHz). The green arrows indicate irradiation at the frequency of H (5b) of 2,6-naphthalenedisulfonate (**5**). These results are consistent with **5** dethreading from the pseudorotaxane structure [**1**$^{4+}$·**5**] as the reaction proceeds.

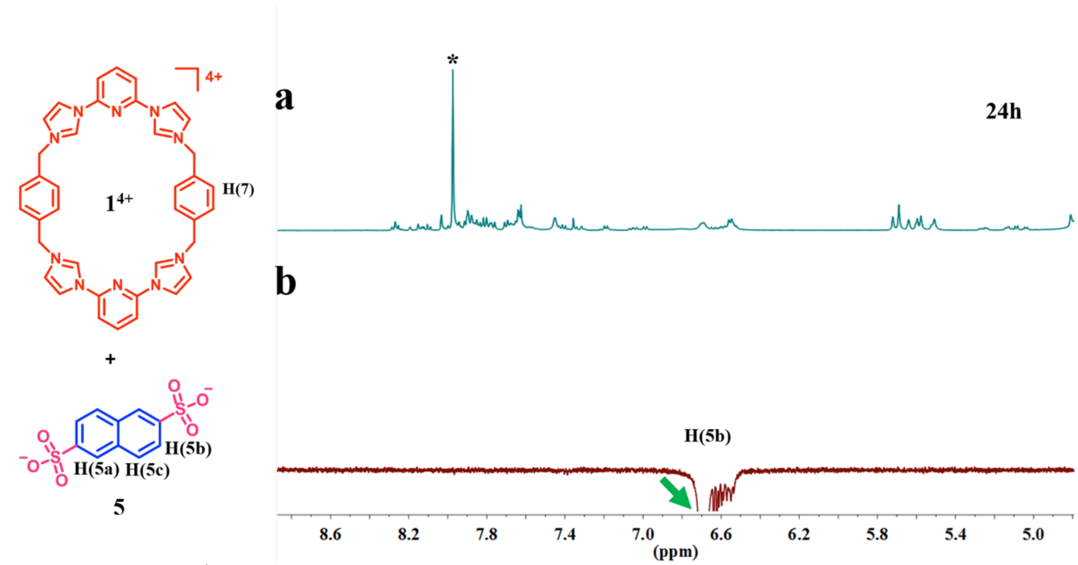

Figure S77. 600 MHz ^{1}H NMR and 1D NOE NMR spectra recorded in DMF-d_7 /D_2O/ND_3·D_2O (1/0.98/0.02, v/v/v) at 298K: (a) ^{1}H NMR of $\mathbf{1}^{4+}$·$4PF_6^-$ recorded in the presence of 1 molar equiv. of 2,6-naphthalenedisulfonic acid and 2 molar equiv. of TMA^+•OH^- at 24 h (reaction time) ([host] = [guest] = 5.00 × 10^{-3} M), (b) 1D NOE NMR spectra of $\mathbf{1}^{4+}$·$4PF_6^-$ in the presence of 1 molar equiv. of 2,6-naphthalenedisulfonic acid and 2 molar equiv. of TMA^+·OH^- at 24 h (reaction time) ([host] = [guest] = 5.00 × 10^{-3} M). The one proton signal of the solvent DMF-d_7 is labeled with a "*". The green arrow indicates irradiation at the frequency of H (5b) of 2,6-naphthalenedisulfonate (**5**).

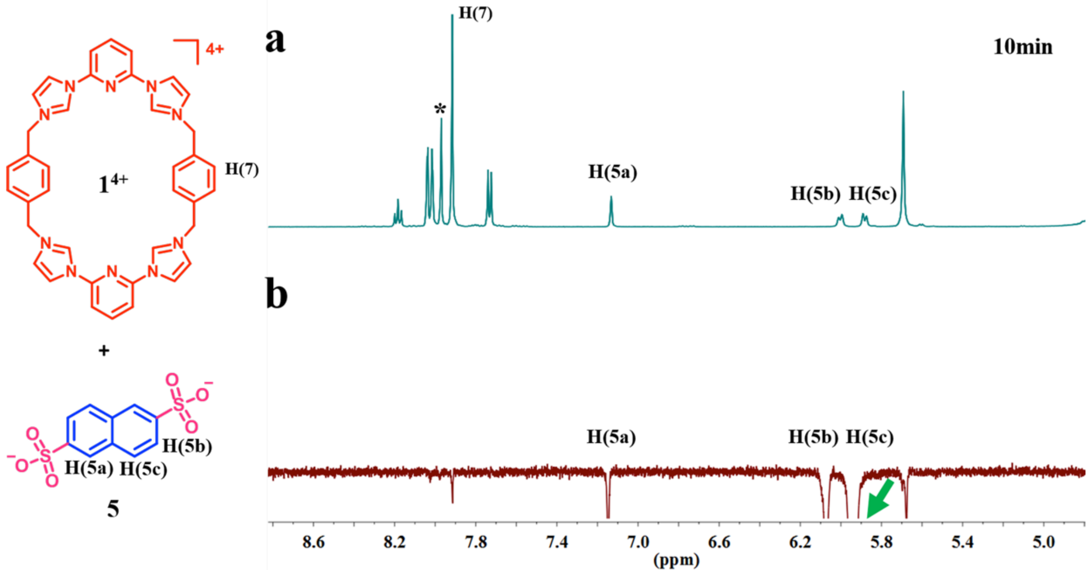

Figure S78. 600 MHz ^{1}H NMR and 1D NOE NMR spectra recorded in DMF-d_7 /D_2O/ND_3·D_2O (1/0.98/0.02, v/v/v) at 298K: (a) ^{1}H NMR of $\mathbf{1}^{4+}$·$4PF_6^-$ in the presence of 1 molar equiv. of 2,6-naphthalenedisulfonic acid and 2 molar equiv. of TMA^+•OH^- at 10min (reaction time) ([host] = [guest] = 5.00 × 10^{-3} M), (b) 1D NOE NMR spectrum of $\mathbf{1}^{4+}$·$4PF_6^-$ recorded in the presence of 1 molar equiv. of 2,6-naphthalenedisulfonic acid and 2 molar equiv. of TMA^+·OH^- at 10 min (reaction time) ([host] = [guest] = 5.00 × 10^{-3} M). The one proton signal of the solvent DMF-d_7 is labeled with a "*". The green arrow indicates irradiation at the frequency of H (5c) of 2,6-naphthalenedisulfonate (**5**).

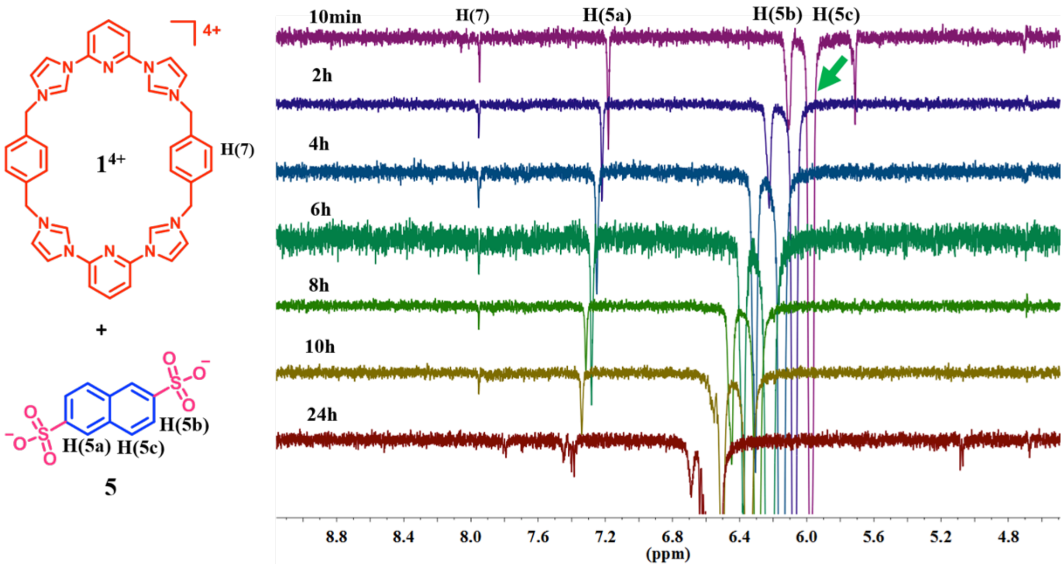

Figure S79. 1D NOE NMR spectra of $\mathbf{1}^{4+}\cdot 4PF_6^-$ recorded in the presence of 1 molar equiv. of 2,6-naphthalenedisulfonic acid and 2 molar equiv. of $TMA^+\bullet OH^-$ ([host] = [guest] = 5.00×10^{-3} M) in DMF-d_7 /D_2O/$ND_3\cdot D_2O$ (1/0.98/0.02, v/v/v) at 298K (600 MHz). The green arrows indicate irradiation at the frequency of H (5c) of 2,6-naphthalenedisulfonate (**5**). These findings are consistent with **5** dethreading from the pseudorotaxane structure [$\mathbf{1}^{4+}\bullet\mathbf{5}$] as the reaction proceeds.

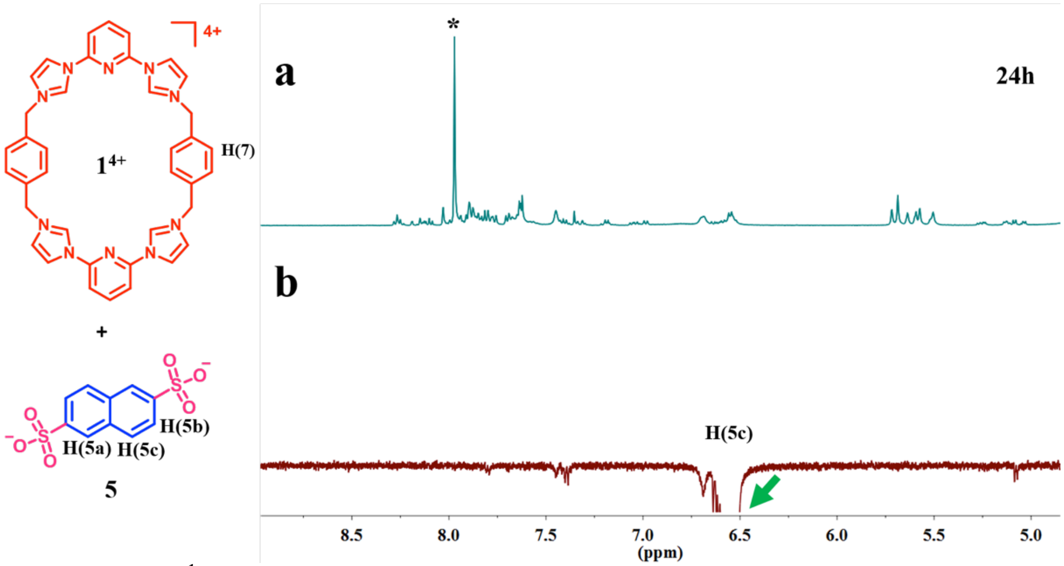

Figure S80. 600 MHz ^{1}H NMR and 1D NOE NMR spectra recorded in DMF-d_7 /D_2O/$ND_3\cdot D_2O$ (1/0.98/0.02, v/v/v) at 298K: (a) ^{1}H NMR of $\mathbf{1}^{4+}\cdot 4PF_6^-$ in the presence of 1 molar equiv. of 2,6-naphthalenedisulfonic acid and 2 molar equiv. of $TMA^+\cdot OH^-$ at 24 h (reaction time) ([host] = [guest] = 5.00×10^{-3} M), (b) 1D NOE NMR spectra of $\mathbf{1}^{4+}\bullet 4PF_6^-$ in the presence of 1 molar equiv. of 2,6-naphthalenedisulfonic acid and 2 molar equiv. of $TMA^+\cdot OH^-$ at 24 h (reaction time) ([host] = [guest] = 5.00×10^{-3} M). The one proton signal of the solvent DMF-d_7 is labeled with a "*". The green arrow indicates irradiation at the frequency of H (5c) of 2,6-naphthalenedisulfonate (**5**).

Section S8:** Comparative analysis of the conformation of [$\mathbf{2}^{2+}\cdot 2PF_6^-$] and [$\mathbf{1}^{4+}\cdot 4PF_6^-$], and the associated complexes formed with anionic guest **4**, **5**, or **6

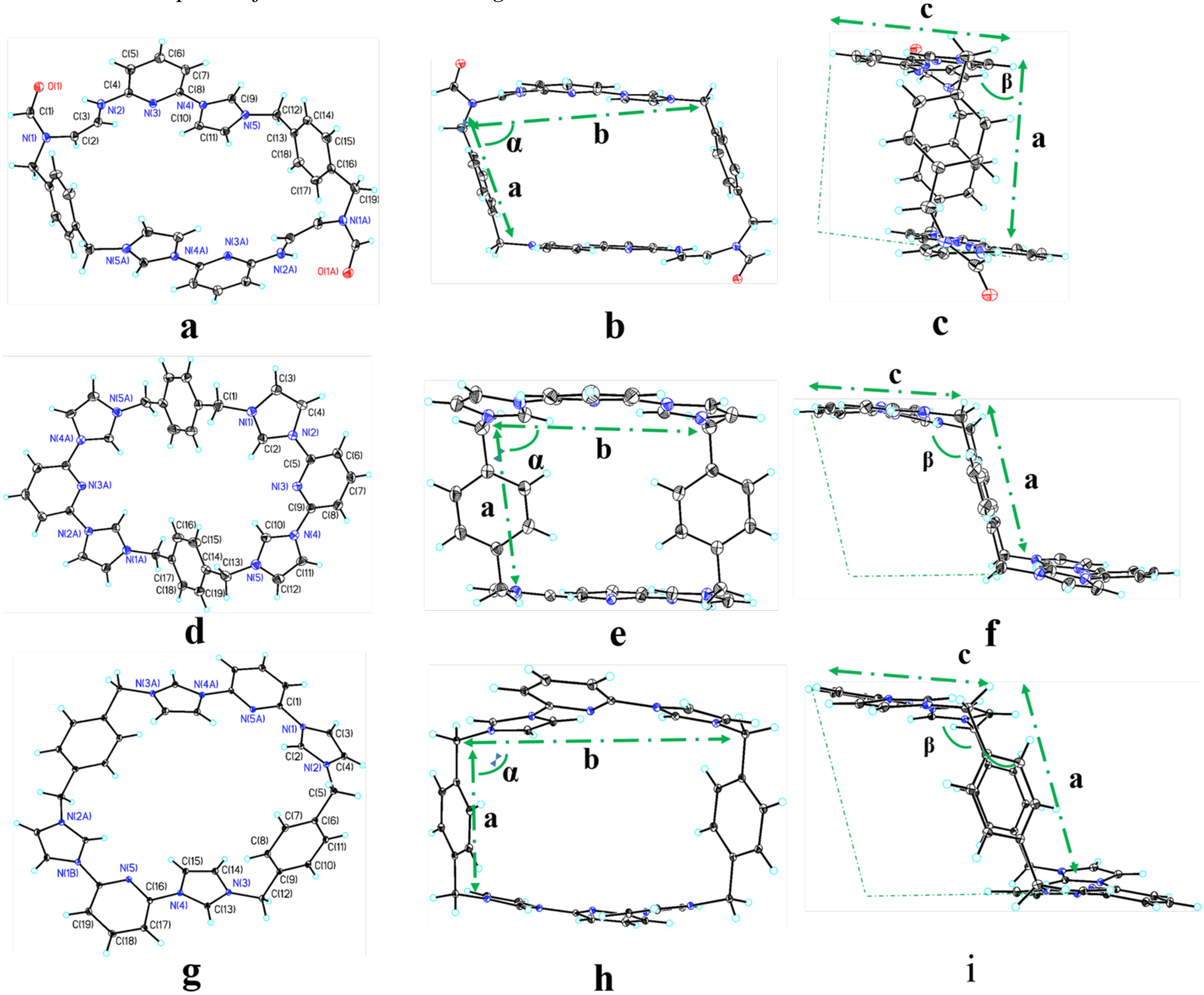

Figure S81. Comparison analysis of the conformations seen in "box-like" structures formed using macrocycle $\mathbf{2}^{2+}$ and $\mathbf{1}^{4+}$. (a) full view, (b) top and (c) side views of the "box-like" conformation of $\mathbf{2}^{2+}$ in [$\mathbf{2}^{2+}\cdot 2PF_6^-\cdot$4dioxane]. (d) full view, (e) top and (f) side views of the "complete-chair" conformation of $\mathbf{1}^{4+}$ in [$\mathbf{1}^{4+}\cdot 4PF_6^-\cdot$4DMF]. (g) full view, (h) top and (i) side views of the "partial-chair" conformation of $\mathbf{1}^{4+}$ in [$\mathbf{1}^{4+}\cdot 4PF_6^-\cdot 2CH_3CN$]. The important parameters for the cavity of $\mathbf{2}^{2+}$ are as follows: Selected distances [Å] a = 5.814, b =11.425, c =4.432; selected angles: α 73.36º, β 92.22º. Important parameters for the cavities formed from $\mathbf{1}^{4+}$ in [$\mathbf{1}^{4+}\cdot 4PF_6^-\cdot$4DMF] are as follows: Selected distances [Å] a = 5.824, b = 7.699, c = 6.112; selected angles: α 85.34º, β 110.0º.[12] Important parameters for the cavities formed from $\mathbf{1}^{4+}$ in [$\mathbf{1}^{4+}\cdot 4PF_6^-\cdot 2CH_3CN$] are as follows: Selected distances [Å] a = 5.818, b = 10.136, c = 4.856; selected angles: α 82.53º, β 122.82º.[11] It is noted that this molecular box $\mathbf{2}^{2+}$ has larger size than the box formed from $\mathbf{1}^{4+}$. With same, if not, very similar width, the latter has a smaller length (9.374 Å).

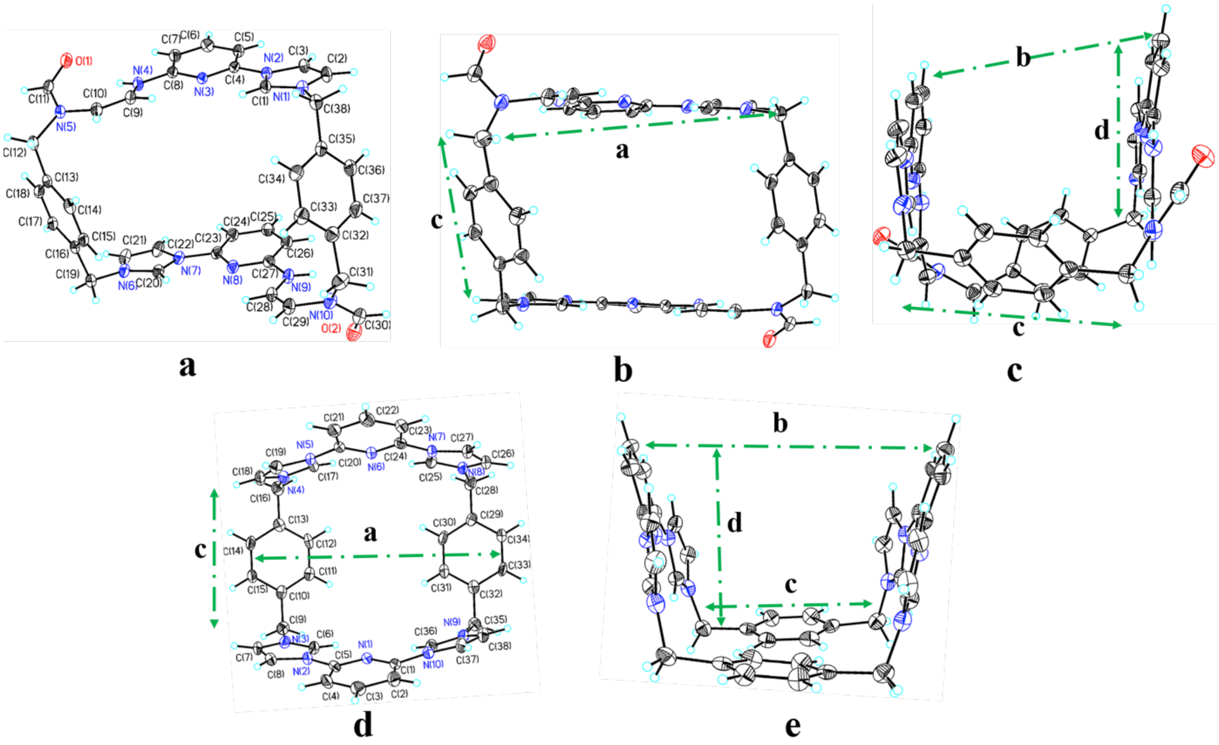

Figure S82. Comparison analysis of the conformations seen in "clip" structures formed using macrocycle **2**$^{2+}$ and **1**$^{4+}$. (a) Full view , (b) top and (c) side views of the "clip" conformation of **2**$^{2+}$ in [**2**$^{2+}$·2PF_6^-·CH_3CN·H_2O]. (d) Top and (e) side views of the "clip" conformation of **1**$^{4+}$ in [**1**$^{4+}$·4PF_6^-·CH_3CN]. Important parameters for the cavities of **2**$^{2+}$ are as follows: Selected distances [Å] a = 9.787, b = 7.485, c = 5.817, d = 5.532. Important parameters for the cavities of **1**$^{4+}$ in [**1**$^{4+}$·4PF_6^-·CH3CN] are: Selected distances [Å] a = 10.314, b = 9.370, c = 5.770, d=5.863.[11] It is found that the top width of **2**$^{2+}$ in its clip conformation is 7.487 Å, which is smaller than for **1**$^{4+}$ with a clip conformation of 9.370 Å.

Table S5. Summary of the limiting binding modes for the complexes formed between **2**$^{2+}$·2PF_6^-, **1**$^{4+}$·4PF_6^- and dianions (**4**, **5**, or **6**).

Guest	Binding mode with **1**$^{4+}$	Binding mode with **2**$^{2+}$
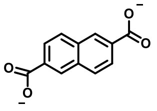 **4**	Pseudorotaxane	"Outside" mode
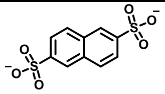 **5**	Pseudorotaxane	"Outside" mode

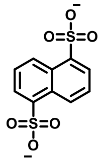<br>**6**	“Outside” mode	“Outside” mode

Table S6. Summary of the calculated association constants, K_a, for the complexes formed between $\mathbf{2}^{2+}\cdot 2PF_6^-$, $\mathbf{1}^{4+}\cdot 4PF_6^-$ and dianions (**4**, **5**, or **6**).

Guest	Stoichiometric ratio and Association constants (K_a) with $\mathbf{1}^{4+}$	Stoichiometric ratio and Association constants (K_a) with $\mathbf{2}^{2+}$
**4**	1:1 (H:G)<br>Ka = (3.5 ± 0.2) × 10^4 M^{-1}	1:1 (H:G)<br>Ka = (2.0 ± 0.1) × 10^2 M^{-1}
**5**	1:1 (H:G)<br>Ka = (1.6 ± 0.1) × 10^3 M^{-1}	1:1 (H:G)<br>Ka = (1.7 ± 0.2) × 10^2 M^{-1}
**6**	1:1 (H:G)<br>Ka = (1.0 ± 0.1) × 10^3 M^{-1}	1:1 (H:G)<br>Ka = (4.0 ± 0.5) × 10^2 M^{-1}

***Section S8:** References*.

(1) W. L. F. Armarego, C. L. L. Chai, Purification of Laboratory Chemicals (Fifth Edition), Elsevier Science: USA, **2003**.

(2) H. E. Gottlieb, V. Kotlyar, A. Nudelman, *J. Org. Chem.* **1997**, 62, 7512.

(3) A. Altomare, M. C. Burla, M. Camalli, G. L. Cascarano, C. Giacovazzo, A. Guagliardi, A. G. G. Moliterni, G. Polidori, R. Spagna, *J. Appl. Cryst.* **1999**, 32, 115.

(4) Sheldrick, G. M. SHELXL97. Program for the Refinement of Crystal Structures. University of Gottingen, Germany, **1994**.

(5) R. I. Cooper, R. O. Gould, S. Parsons, D. J. Watkin, *J. Applied. Cryst.* **2002**, 35, 168.

(6) L. J. Farrugia, *J. Applied. Cryst.* **1999**, 32, 837.

(7) G. M. Sheldrick, *Acta Cryst.* **2008**, A64**,** 112.

(8) P. Job, *Ann. Chim*. Paris, **1928**, 9**,** 113.

(9) Hyperquad2003: P. Gans, A. Sabatini, A. Vacca, *Talanta*, **1996**, 43, 1739.

(10) Ōki, M. *Applications of Dynamic NMR Spectroscopy to Organic Chemistry*; VCH, Weinheim, **1985**.

(11) H.–Y. Gong, B. M. Rambo, E. Karnas, V. M. Lynch, J. L. Sessler, *Nature Chem*. **2010**, 2, 406.

(12) H.–Y. Gong, B. M. Rambo, E. K. Karnas, V. M. Lynch, K. M. Keller, J. L. Sessler, *J. Am. Chem. Soc*. **2011**, 133**,** 1526.
